# TME-analyzer: a new interactive and dynamic image analysis tool that identified immune cell distances as predictors for survival of triple negative breast cancer patients

**DOI:** 10.1038/s44303-024-00022-6

**Published:** 2024-07-25

**Authors:** Hayri E. Balcioglu, Rebecca Wijers, Marcel Smid, Dora Hammerl, Anita M. Trapman-Jansen, Astrid Oostvogels, Mieke Timmermans, John W. M. Martens, Reno Debets

**Affiliations:** 1 Laboratory of Tumor Immunology, Department of Medical Oncology, Erasmus MC Cancer Institute, Rotterdam, The Netherlands; 2 Laboratory of Translational Cancer Genomics, Department of Medical Oncology, Erasmus MC Cancer Institute, Rotterdam, The Netherlands; 3 Present Address: Pan Cancer T, Discovery & Research, Rotterdam, The Netherlands

**Keywords:** Cancer imaging, Image processing, Microscopy, Imaging the immune system

## Abstract

Spatial distribution of intra-tumoral immune cell populations is considered a critical determinant of tumor evolution and response to therapy. The accurate and systemic search for contexture-based predictors would be accelerated by methods that allow interactive visualization and interrogation of tumor micro-environments (TME), independent of image acquisition platforms. To this end, we have developed the TME-Analyzer, a new image analysis tool, which we have benchmarked against 2 software tools regarding densities and networks of immune effector cells using multiplexed immune-fluorescent images of triple negative breast cancer (TNBC). With the TME-Analyzer we have identified a 10-parameter classifier, predominantly featuring cellular distances, that significantly predicted overall survival, and which was validated using multiplexed ion beam time of flight images from an independent cohort. In conclusion, the TME-Analyzer enabled accurate interactive analysis of the spatial immune phenotype from different imaging platforms as well as enhanced utility and aided the discovery of contextual predictors towards the survival of TNBC patients.

## Introduction

Spatial architecture of the tumor microenvironment (TME), particularly in relation to quantity, subsets, and distances among immune cell populations has prognostic and predictive value for multiple (sub)types of solid tumors^1–4^. The prognostic value of the immune contexture is best captured when multiple parameters are combined and inter-related, superseding the mere expression of single markers^5,6^. To enable multi-parameter spatial studies, novel multiplexed microscopy methods have been developed. Such methods make use of repetitive staining and imaging^7,8^, spectral deconvolution of overlapping fluorophores^9^, mass cytometry coupled to ablation of tissues^10^, or release of conjugated reporters^11^.

As different techniques have become widely available and complexity of images significantly increased, consistent and user-friendly analysis of such data, and its relationship to clinical outcomes, have become more and more challenging. See reviews^12–16^ for a comprehensive list on imaging and analysis techniques and their strengths and shortcomings. In short, publicly available tools, such as ImageJ^17^ and CellProfiler^18^, are widely used software tools for analysis, yet are not specifically geared towards high-dimensional images making their interfaces hard to navigate for non-expert users. Commercial alternatives may provide more specific tools, yet generally come with high licensing costs. The same challenges hold true for approaches that enable quantitation of processed images, which require analysis by expert personnel^16^, as well as for machine learning approaches, which require specific hardware/software and input from pathologists to ensure their development. Notably, these approaches often rely on closed software that does not provide the option to inspect and edit analysis and/or perform manual adjustments depending on the tissue or study question. Such inspection and editing options, however, are a necessity in case of bleed-through correction, inclusion of defined tissue areas and/or cell phenotyping according to structural features. In fact, the biggest roadblock in maximizing the potential of high-dimensional imaging has been the inability to customize settings to accurately address intra- and inter-tissue heterogeneity as highlighted in recent reviews^15,16,19^. Collectively, there is a need for a dedicated and user-friendly analysis tool that encompasses multiple image platforms.

To address the above shortcomings, we have developed TME-Analyzer: a Python-based, what-you-see-is-what-you-get (WYSIWYG), interactive graphical user interface (GUI). TME-Analyzer is compatible with fluorescent and other high-dimensional images that have a nuclear marker for cell detection. Specifically, this interface comes with integrated quantification of cellular and tissue phenotypes, their densities in defined compartments and their interspacing, as well as visualization and exportation of data. In this report, we introduce the TME-Analyzer and present its performance and applicability as a stand-alone tool for start-to-finish image analysis. First, we characterized the immune contexture from inflamed and non-inflamed triple-negative breast cancer (TNBC) tissue of 63 patients, with findings in good concordance with two benchmark software tools. Second, we built a prognostic classifier towards overall survival for this cohort of patients, and have validated the classifier in an independent cohort of TNBC MIBI-TOF (multiplexed ion beam imaging by time of flight) data^11^.

## Results

### TME-analyzer, an interactive and customizable image analysis tool

**Fig. 1:**
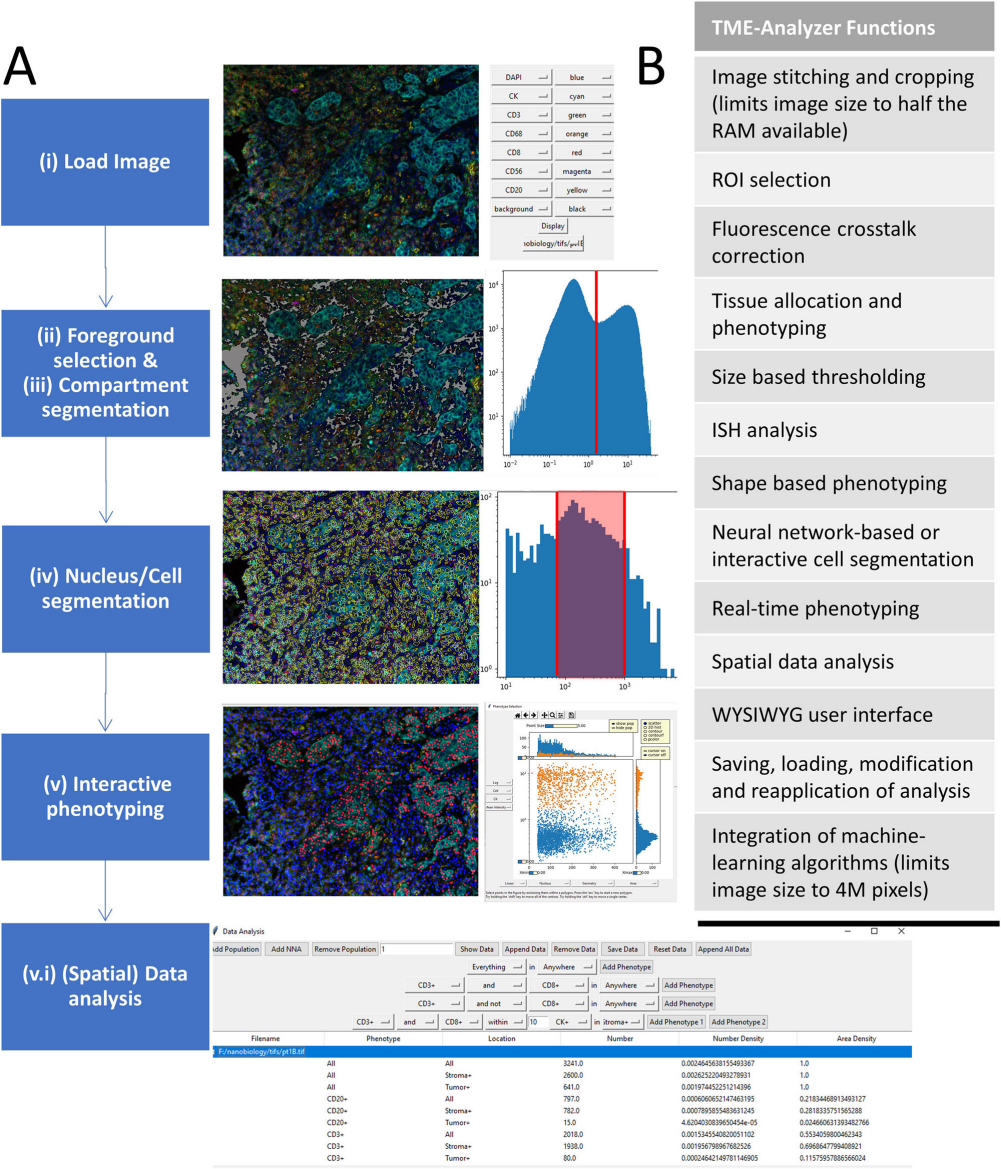
TME-analyzer’s workflow and functions. **A** Image analysis workflow, listing individual steps for obtaining data from a multichannel image (left), together with examples of corresponding image and data presentations according to the TME-Analyzer’s graphical interface (right). **B** List of functions of TME-Analyzer that are part of the presented workflow as well as additional modules to facilitate interrogations of tissue images.

TME-Analyzer was developed to address the high inter- and intra-patient heterogeneity, which is inherent to images of cancerous tissue particularly with regard to intensities, and to yield a tool that allows intuitive analysis in an easy and semi high-throughput manner. The image analysis workflow of TME-Analyzer is displayed and exemplified in Fig. 1A and consists of the following 6 steps: (i) image loading; (ii) foreground selection; (iii) compartment segmentation; (iv) nucleus/cell segmentation; (v) cell phenotyping; and (vi) data analysis and exportation. Following the first step, i.e., image loading, intensity histograms per channel (i.e., marker) are generated to enable foreground selection and compartment segmentation. During the latter two steps, these histograms are used to select thresholds and correct for uneven background signals. This is followed by nucleus segmentation, which is performed either manually using a watershed algorithm on nuclear signals assigned during compartment segmentation, or through a machine learning approach^20^. The segmented nuclei are then used as seeds for Voronoi cell segmentation^21^. Next, cell phenotyping is performed with flow cytometry-like gating, where selected cells are back-projected to the tissue image in real-time for visualization and adaptation of the phenotyping. Finally, in the last step, data analysis is performed, i.e., quantification of tissue areas, cellular numbers, densities, and distances among cells and tissues with defined phenotypes, using the integrated module of the TME-Analyzer software. The analysis outcome per image is saved as single-cell and tissue information (i.e., location, geometrical parameters such as area and perimeter, intensities, phenotyping), as well as the full analysis procedure for loading and reapplication. Once the analysis is set up for one image, it can then be applied to a cohort of images, with the option to adjust analysis parameters per individual image when needed. To compare quantifications between images, the data analysis for the whole cohort is assembled and exported as data tables for visualization. An overview of the functionalities of TME-Analyzer is shown in Fig. 1B.

**Fig. 2:**
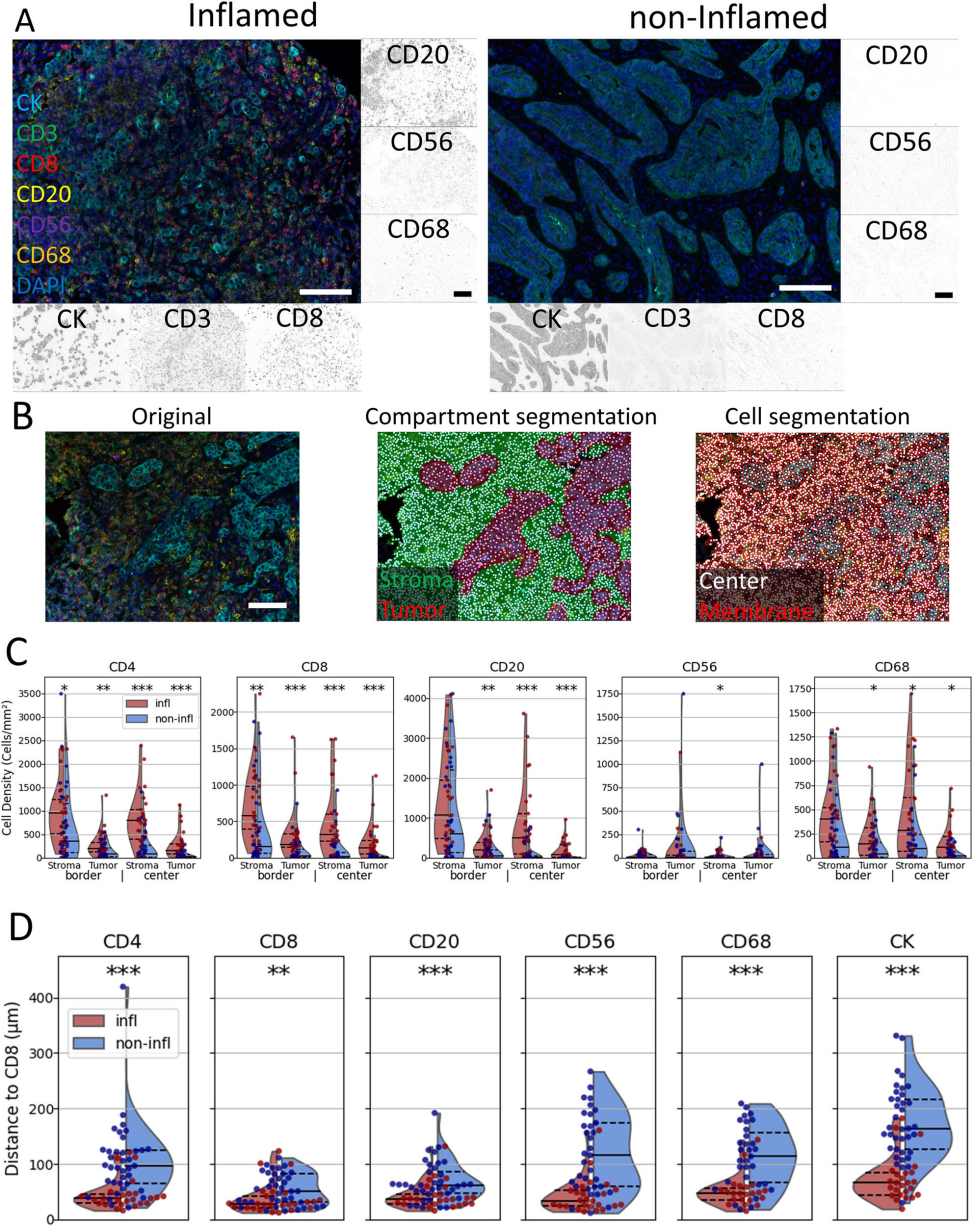
TME-analyzer captures densities and inter-cellular distances of immune effector cell populations in TNBC. **A** Representative multiplex images and images corresponding to individual channels of inflamed (left) and non-inflamed (right) tumors. **B** Representative image (left) with tissue segmentation (middle) showing tumor (red) and stroma (green) compartments and cell segmentation (right) showing cell center (white) and cell membrane (red) performed using TME-Analyzer (**C**). Violin and scatter plots of densities of different phenotypes of immune cells, together with median (solid black line) and 25% quartiles (dashed black lines), in stroma and tumor compartments of border and center regions in cells per mm^2^ for inflamed (red) and non-inflamed (blue) tumors (**D**). Violin and scatter plots of distances between different phenotypes of immune cells and CD8 T cells, together with median (solid black line) and 25% quartiles (dashed black lines), in all regions/compartments for inflamed and non-inflamed tumors in μm. Scale bars (**A**, **B**) are 100 μm. *p < 0.05, **p < 0.01, ***p < 0.001 according to Mann–Whitney *U* test comparing data from inflamed to non-inflamed tumors.

To put the TME-Analyzer to the test, we imaged tumor border and center regions with Multiplexed immunofluorescence (MxIF) in whole slide sections of 63 primary TNBC patients and assessed the presence of CD3, CD8, CD20, CD56, CD68 and pan-Cytokeratin (CK)-positive cells. Patients’ tumors were previously annotated as inflamed and non-inflamed according to numbers and locations of CD8 T cells^2^ (*n* = 25 and 38, for inflamed and non-inflamed, respectively), of which representative images are shown in Fig. 2A. Using TME-Analyzer, we re-defined tumor and stroma compartments per image and identified and phenotyped individual cells for positivity of markers in each compartment in border and center regions using our previous report^2^ as a reference (Fig. 2B, Supplementary Table 1). Analysis of densities of the different cellular phenotypes per patient (Supplementary Table 2) revealed a highest overall abundance of immune effector cells at the stroma compartment of the border region for both inflamed and non-inflamed tumor tissues, with CD20 cells having the highest density followed by CD4 and CD8 T cells (Fig. 2C). All immune cells showed higher abundances in inflamed versus non-inflamed tumors, particularly at the center-stroma. Significantly higher densities of CD8 and CD4 T cells were observed in inflamed tumors compared to non-inflamed tumors for all compartments (Fig. 2C). Furthermore, the average distance of individual cell phenotypes to the nearest CD8 T cell in all compartments was lower in inflamed when compared to non-inflamed tumor tissues (Fig. 2D). The quantification of all nearest-neighbor distances for individual tumor and stroma compartments is listed in Supplementary Table 2. Collectively, these outcomes demonstrate that the TME-Analyzer correctly captures the differential presence and spatial organization of immune effector cell populations in inflamed and non-inflamed TNBC.

### TME-analyzer shows high concordance regarding cellular densities and networks with benchmark software tools

**Fig. 3:**
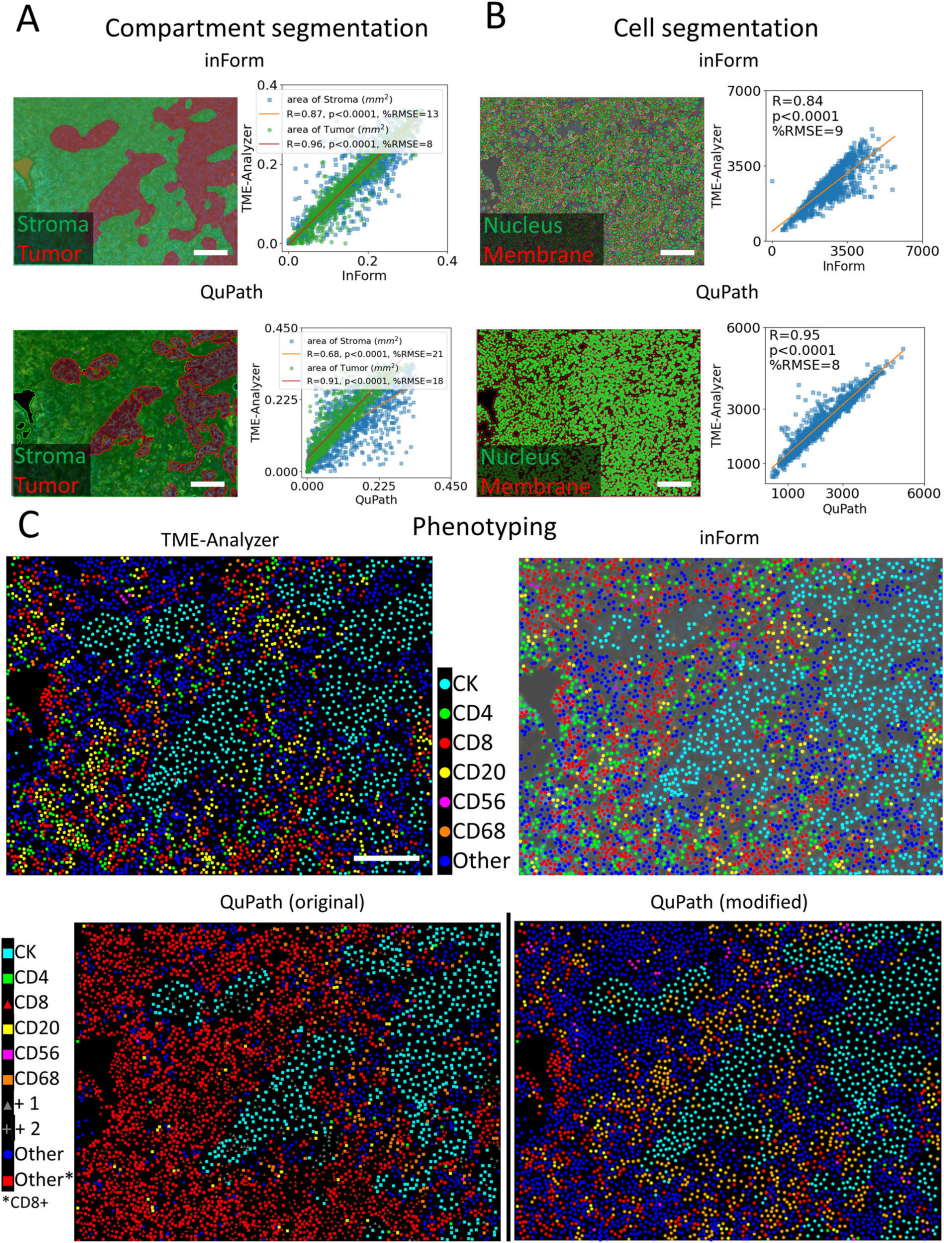
TME-analyzer is in high concordance with benchmark software tools regarding image analysis of TNBC. **A**, **B** Representative images of tissue (**A**, left) and cell segmentation (**B**, left) analyzed using inForm (top) and QuPath (bottom) and corresponding quantifications compared with TME-Analyzer (right) using scatter plots and Pearson correlations between TME-Analyzer and benchmark software tools for all images. **C** Representative images for phenotyped cells using TME-Analyzer (left, top), inForm (right, top) and QuPath (bottom) with original visualization from QuPath with its unique phenotype legend (left) and phenotype visualization modified for ease of comparison (right). In scatter plots, linear approximation with Pearson coefficient (R) and range normalized differences as percentage root mean square error (RMSE) are shown between TME-Analyzer and benchmark software tools. All correlation coefficients were significantly non-zero (*p* < 0.0001) according to the Wald test. Scale bars (**A**–**C**) are 100 μm.

**Fig. 4:**
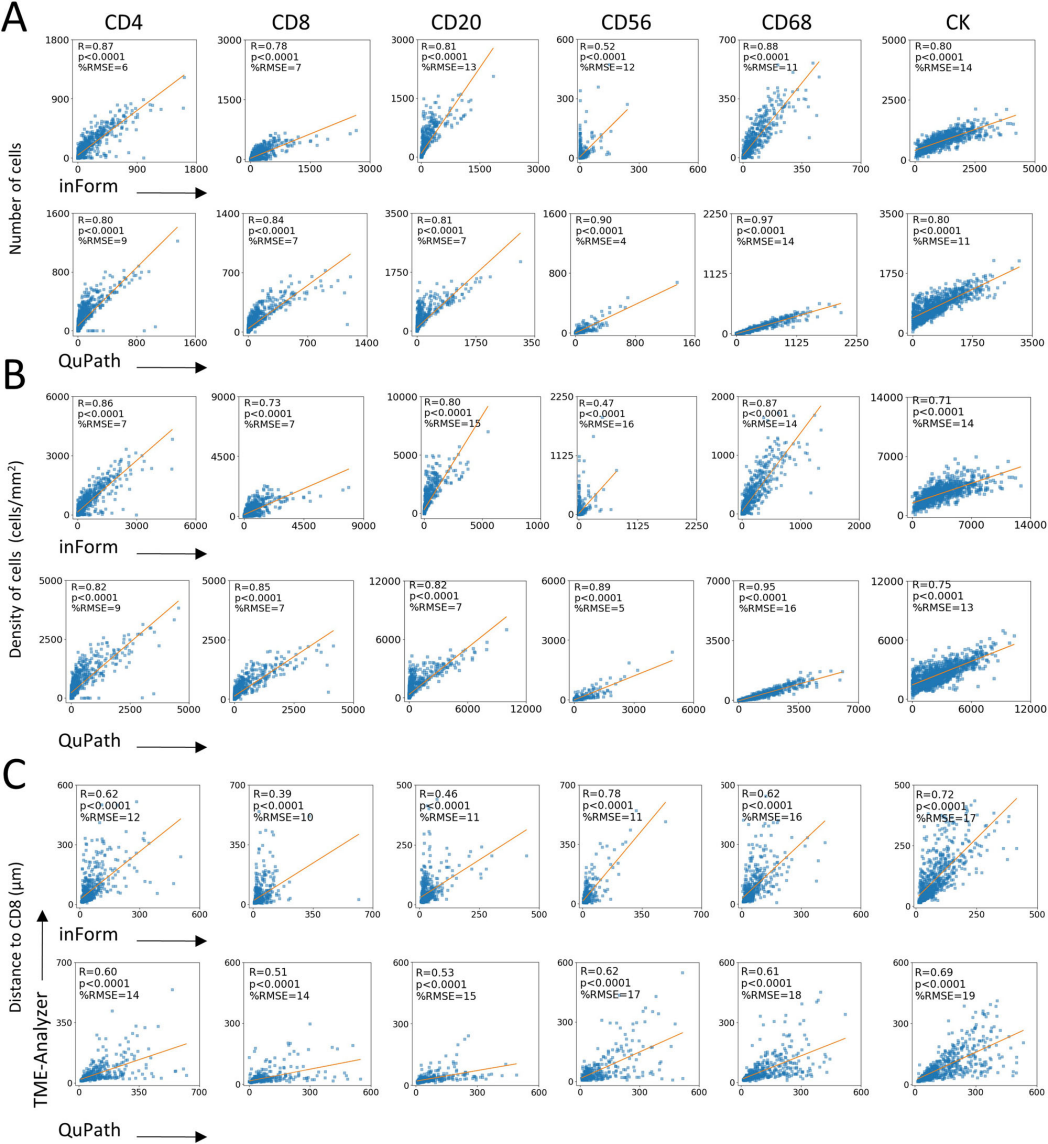
TME-analyzer is in high concordance with benchmark software tools regarding immune contexture of TNBC. **A**–**C** Scatter plots comparing analysis performed by TME-Analyzer (*y*-axis) vs inForm (*x*-axis, top) or QuPath (*x*-axis, bottom) for numbers (**A**) and densities (**B**) of phenotyped cells as well as their distances to CD8 T cells (**C**). In all scatter plots, linear approximations with Pearson coefficient (R) as well as range-normalized differences in percentage root mean square error (RMSE) are shown between TME-Analyzer and the two benchmark software tools. All correlation coefficients were significantly non-zero (*p* < 0.0001) according to the Wald test.

The outcomes of the TME-Analyzer were directly compared to the outcomes with inForm® (Akoya Biosciences) tissue analysis software^22^ (Supplementary Table 3, 4) and QuPath^23^ open-source software (Supplementary Table 4). InForm, on the one hand, is an established commercial MxIF analysis software^15^, which we also previously utilized towards analysis of immune contextures^1–3^. QuPath, on the other hand, is an established open-source image analysis software, which also supports multiplexed image analysis since 2020, version 0.2. Compartment segmentation (Fig. 3A), cell segmentation (Fig. 3B), and phenotyping (Fig. 3C), generated highly comparable results for TME-Analyzer when compared to inForm and QuPath. Importantly, an image-to-image comparison between the 3 software tools revealed that tissue areas and cell numbers demonstrate statistically significant concordance between TME-Analyzer and inForm or QuPath (Fig. 3A, B). In addition, the quantification of abundance of cellular phenotypes (Fig. 4A), their densities (Fig. 4B), and distances to CD8 T cells (Fig. 4C), also showed significant concordance and less than 20% root mean square error between TME-Analyzer and the benchmark software tools inForm and QuPath.

Expectedly, inForm and QuPath, similar to TME-Analyzer (Fig. 2), also captured differential densities of immune effector cells (Supplementary Fig. 1A) and their distances to CD8 T cells (Supplementary Fig. 1B) in inflamed versus non-inflamed tumor tissues. For example, with inForm and QuPath highest densities of immune effector cell populations in border-stroma of both inflamed and non-inflamed tumor tissues were observed (Supplementary Fig. 1A), as well as much lower densities in the center-stroma of the non-inflamed tumors and shorter distances to CD8 T cells in inflamed tumors (Supplementary Fig. 1B).

In conclusion, the TME-Analyzer was able to analyze immune contextures equally well as inForm or QuPath (Supplementary Table 4). Comparison of the tissue segmentation (Fig. 3, Supplementary Fig. 2) and cell phenotyping (Fig. 4, Supplementary Fig. 3) between software tools, resulted in similar percentage root mean square error between software tools (Fig. 4). Given this similarity, and our previously published analysis regarding this patient cohort with inForm, we zoomed in on comparing TME-Analyzer with inForm.

### TME-analyzer demonstrates improved accuracy of spatial phenotyping compared to inForm

When comparing TME-Analyzer to inForm regarding tissue segmentation, we did note that TME-Analyzer provided more accurate tissue segmentation (Fig. 3A, Supplementary Fig. 2) as well as CD56 phenotyping (Fig. 4A, Supplementary Fig. 4A, B). Additionally, due to differences in tissue autofluorescence, inForm required multiple algorithms for quantification, whereas an autofluorescence analytical correction with a minimal cut-off used in TME-Analyzer automatically addressed this challenge in the subset of images with high autofluorescence. TME-Analyzer also performed better with respect to tagging cells with single or multiple phenotypes, which is also illustrated along 3 lines of observations. First, in TME-Analyzer, but not inForm, cell phenotyping was performed independently per marker. For instance, using TME-Analyzer, but not inForm, CD3 + CD56+ double positive natural killer T cells can be detected despite their relative rarity (Supplementary Fig. 4C, D). This subpopulation was however not further studied due to its very low abundance. Second, when assessing the accuracy of cell phenotyping performed with TME-Analyzer (Supplementary Fig. 3), we observed that TME-Analyzer in contrast to inForm does not falsely annotate cells as positive for CK in case cells are in the vicinity of CK-positive regions (Supplementary Fig. 5). And third, TME-Analyzer classified CD3 + CD8+ cells, but not CD3 − CD8+ cells, as CD8 T cells, whereas inForm classified both CD3 + CD8+ cells as well as CD3 − CD8+ cells as CD8 T cells, even though the latter tool was trained with CD3 + CD8+ cells (Supplementary Fig. 6).

Taken together, while output from the software tools is highly comparable at capturing numbers and locations of immune effector cell populations, the TME-Analyzer, compared to inForm, provides practical advantages (faster and easier to navigate) and yields a more accurate outcome regarding cellular phenotypes.

### TME-analyzer facilitated the identification of a contextual classifier towards survival in TNBC

**Fig. 5:**
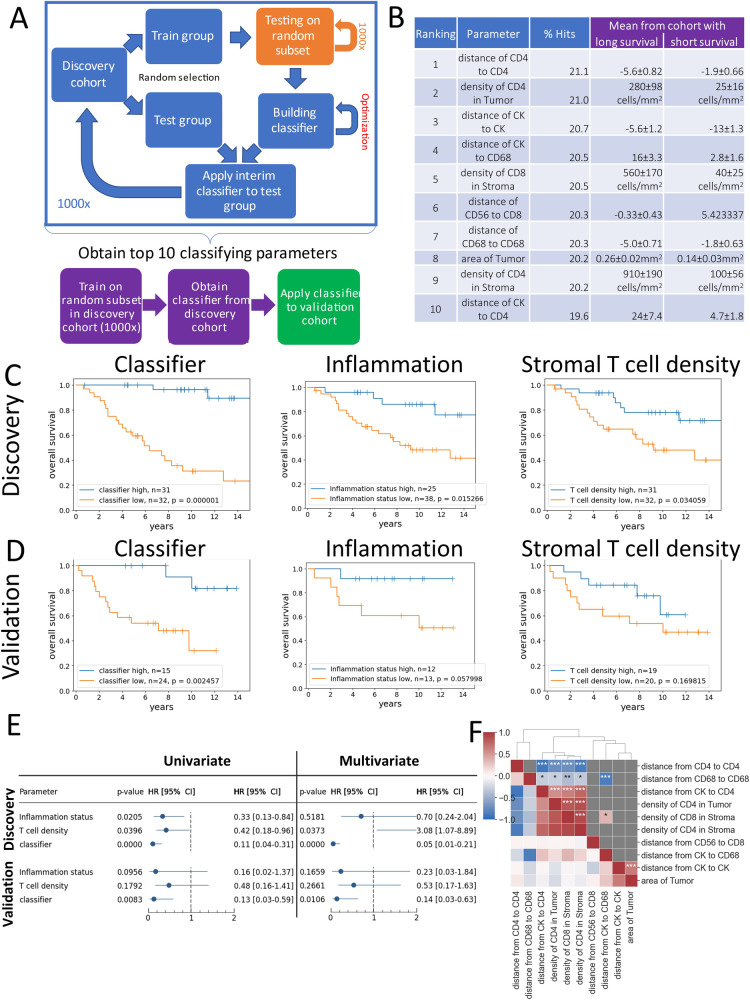
Discovery, validation and performance of a contextual classifier towards survival of TNBC patients. **A** Workflow to rank parameters according to prognostic performance using the discovery cohort (top), to build a classifier using a nested Monte-Carlo approach, and to test the classifier using the validation cohort (bottom). **B** Top-10 parameters ranked according to the number of hits in interim classifiers (blue) and their Monte-Carlo classifier means using the discovery cohort (purple). **C**, **D** Kaplan-Meier curves for survival of 2 groups of patients segregated using median split when applying the final classifier from B (left), inflammation status (middle) or stromal T cell density (right) using the discovery (**C**) and validation (**D**) cohorts with *p*-values calculated according to log-rank test. **E** Hazard ratios (HR) with 95% confidence interval (CI), also presented as a forest plot, and *p*-value obtained by applying univariate and multivariate COX analysis to classifier, inflammation status and stromal T cell density. **F** Spearman correlations of top-10 parameters with upper-triangular showing only significant correlations and dendrogram showing clustering based on the correlations. *p < 0.05, **p < 0.01, ***p < 0.001 according to log-rank test (**D**), non-parametric Cox analysis using Breslow’s method (**E**) and Spearman correlation (**F**).

To further put the TME-Analyzer to the test, we set out to identify immune contextual markers that relate to overall survival in TNBC. To this end, we have implemented a methodology to rank the parameters obtained using TME-Analyzer (Fig. 5A, top; Fig. 5B, blue), and built a classifier around the top 10 parameters (Fig. 5A, bottom; Fig. 5B, purple). Starting from a total of 438 different parameters from a discovery cohort of TNBC patients (Supplementary Table 2), only those parameters obtained from the tumor center were considered (*n* = 146 parameters) as this would enable proper validation (see “Materials and Methods” for description of discovery and validation cohorts). These 146 parameters were then further refined to reduce interdependency. Along this line, we noted that numbers, densities and intercellular distances per phenotype were highly correlated (Supplementary Fig. 7A, B), and have excluded numbers of cells from further analysis as this parameter is captured by combining density and area. Distances were quantified as *z*-scores to account for its dependence on cell density (see “Materials and Methods”). This resulted in a shortlist of 50 parameters that consisted of areas of tumor and stroma compartments, and densities and distance *z*-scores of 6 different cell phenotypes (Supplementary Fig. 7C, D). Using the nested Monte-Carlo approach, and re-iterative testing, these 50 parameters were ranked for their prognostic value, which ultimately yielded a classifier that consisted of the most discriminative set of 10 parameters (for details, see “Materials and Methods” and Fig. 5A, B).

The final classifier was able to maximally segregate the discovery set into 2 groups of 31 and 32 patients with differential prognosis, namely having a 5-year OS of 100 vs 58.1% and a 10-year OS of 94.4 vs 25.9% (Fig. 5C, left, log-rank *p*-value = 0.000001). We validated our classifier using an independent second patient cohort, to which end we used MIBI-TOF images from TNBC tumors, that were obtained with a set of 36 markers including the 7 markers we used^11^. To make use of this validation cohort, we have re-analyzed the MIBI-TOF images with the TME-Analyzer similarly as for the MxIF images, except for marker thresholds (for details, see “Materials and Methods” and Table 1). The re-analysis demonstrated that contextual phenotypes per image were in good agreement between the two analysis tools (Supplementary Fig. 8, Supplementary Table 5). The classifier grouped patients of the validation cohort again in 2 groups of 15 and 24 patients that had significantly different prognosis, namely a 5-year OS of 100 vs 52.2% and a 10-year OS of 90.9 vs 13.3% (Fig. 5D, left, log-rank *p*-value = 0.0025). Testing different classification thresholds by varying the number of top keys (Supplementary Table 6) suggested that a minimum number of six keys is required for accurate validation (Supplementary Table 7). When we specifically utilized the classifier towards the identification of patients with less than 5-years OS (*n* = 11) from patients with more than 10-years OS (*n* = 12) in the validation cohort, it had a sensitivity of 100% (*n* = 11) and specificity of 84.6% (11 out of 13).

**Table 1:** TME-Analyzer: steps and parameter settings for analysis of MxIF and MIBI-TOF images

	Discovery (MxIF)	Validation (MIBI-TOF)
i. Load image		
ii. Foreground detection	Background-correction with 1000-px	
Threshold	0.1 in all channels	0 in all channels
Remove artifacts	Background and Foreground areas smaller than 5000-px	Background and Foreground areas smaller than 250-px
iii. Compartment segmentation	CK image gaussian filter (size: 10-px), and minimum filter subtraction (size: 1000-px)	
Threshold	1	0.5
Remove small regions	Stroma and Tumor areas smaller than 5000-px	
iv. Cell segmentation	StarDist “2D_versatile_fluo”^20^ and Voronoi^21^	
v. Phenotyping	Background-correction with 1000-px	
CD8 threshold	0.4	0
CD20 threshold	0.7	0
CD3 threshold	0.3	0
CD68 threshold	1	0
CD56 threshold	0.7	0
CK threshold	0.1	0

Image analysis workflow (Fig. 1A) and parameters used with limited adaptations for images obtained using two different platforms.

Given T cell infiltration is highly associated with survival (see “Introduction”) and rank highly in our approach (Fig. 5B), we next wanted to test if T cell parameters can alone be prognostic. Interestingly, splitting patients into 2 groups according to inflammation status (see “Materials and Methods”) or stromal T cell stromal density (from TME-Analyzer) revealed statistically different survival in the discovery cohort (Fig. 5C, middle and right), but not the validation cohort (Fig. 5D, middle and right). Univariate COX analysis with inflammation, T cell density and the classifier revealed that while all three parameters were significantly associated for survival in the discovery cohort, only the classifier was significant in the validation cohort (Fig. 5E). Moreover, upon multivariate analysis, the inflammation status also lost its significance in the discovery cohort (Fig. 5E).

The 10 parameters that drive the classifier (Fig. 5B) comprise: tumor area; density of CD4 T cells in tumor and stroma, and that of CD8 T cells in stroma; as well as the CD4-CD4; CD8-CD56; CD68-CD68; CD4-CK; CD68-CK; and CK-CK distances. According to the Cox regression model, 6 of these parameters were significantly associated with survival in the discovery cohort, but none of these were significant in the validation cohort (Table 2). Regarding these 10 parameters, statistically significant positive correlations were observed among densities of CD4 T cells in tumor and stoma, CD8 T cells in stroma and longer CK-CD4 distances, which in turn were all negatively correlated with longer CD68-CD68 and CD4-CD4 distances (Fig. 5F). Furthermore, the CK-CD68 distances positively correlated with stromal CD8 T cell density, but negatively with the CD68-CD68 distances. From the 10 parameters, only the CD56-CD8 distances did not show any correlation with any other parameter (Fig. 5F).

**Table 2:** Contextual parameters with prognostic value for TNBC patients.

Parameter	Discovery		Validation	
	HR (95% CI)	*p*-value	HR (95% CI)	*p*-value
distance from CD20 to CD4 in Stroma in border	0.27 (0.11–0.64)	0.0029		
density of CD8 in Tumor	0.29 (0.12–0.67)	0.0039		
distance from CD8 to CK	0.31 (0.13–0.72)	0.0066		
**number of CD8 in Tumor in center**	0.31 (0.13–0.72)	0.0068	0.29 (0.09–0.92)	0.0349
density of CD8 in border	0.31 (0.13–0.72)	0.0068		
distance from CD20 to CD68 in Stroma	0.24 (0.08–0.67)	0.0069		
density of CD4 in Stroma	0.31 (0.13–0.73)	0.0075		
distance from CD4 to CD68 in Stroma	0.27 (0.10–0.72)	0.0088		
distance from CK to CK in center*	0.31 (0.13–0.75)	0.0090	0.87 (0.31–2.46)	0.7942
density of CD8 in Stroma	0.32 (0.14–0.76)	0.0093		
density of CD20 in Stroma in center	0.33 (0.14–0.76)	0.0093	0.39 (0.13–1.16)	0.0898
distance from CD4 to CD68 in Stroma in border	0.28 (0.10–0.74)	0.0105		
distance from CK to CD4 in center*	0.33 (0.14–0.78)	0.0110	0.55 (0.19–1.61)	0.2726
distance from CD20 to CD20 in Stroma in border	2.96 (1.28–6.87)	0.0115		
number of CD8 in Stroma	0.35 (0.15–0.80)	0.0128		
distance from CD4 to CD4 in center*	2.95 (1.26–6.93)	0.0129	2.61 (0.89–7.67)	0.0803
density of CD8 in Tumor in border	0.35 (0.15–0.80)	0.0132		
**density of CD20 in Tumor in center**	0.34 (0.15–0.80)	0.0133	0.25 (0.08–0.78)	0.0171
distance from CD4 to CD4 in Tumor in center	2.92 (1.25–6.82)	0.0134	1.36 (0.49–3.77)	0.5504
**number of CD20 in Tumor in center**	0.35 (0.15–0.81)	0.0141	0.26 (0.08–0.82)	0.0219
density of CK in Stroma in border	2.90 (1.22–6.87)	0.0155		
number of CD8 in Tumor in border	0.36 (0.16–0.83)	0.0164		
distance from CD20 to CD8	0.36 (0.16–0.83)	0.0166		
number of CD8	0.37 (0.16–0.84)	0.0173		
distance from CD20 to CD68	0.30 (0.11–0.81)	0.0182		
distance from CK to CD8 in Tumor	0.34 (0.14–0.84)	0.0189		
distance from CK to CD8 in center	0.32 (0.12–0.84)	0.0201	1.00 (0.36–2.78)	0.9951
distance from CD56 to CD4 in Tumor in center	4.64 (1.27–16.93)	0.0202	0.95 (0.32–2.78)	0.9232
number of CD8 in border	0.38 (0.17–0.86)	0.0207		
density of CD8 in Stroma in border	0.38 (0.17–0.86)	0.0207		
distance from CD8 to CD8 in border	2.66 (1.16–6.11)	0.0212		
distance from CD20 to CD68 in Stroma in border	0.32 (0.12–0.85)	0.0224		
distance from CD56 to CD4 in center	3.40 (1.19–9.75)	0.0225	1.34 (0.49–3.71)	0.5714
number of CD4	0.38 (0.17–0.88)	0.0228		
density of CD4 in border	0.38 (0.17–0.88)	0.0228		
density of CD4	0.38 (0.17–0.88)	0.0228		
distance from CD4 to CD20	0.36 (0.15–0.87)	0.0231		
distance from CD4 to CD20 in Stroma	0.37 (0.16–0.87)	0.0232		
**density of CK in Stroma in center**	2.69 (1.14–6.33)	0.0238	3.25 (1.03–10.23)	0.0444
number of CD8 in Stroma in border	0.39 (0.17–0.88)	0.0238		
density of CD8	0.39 (0.17–0.88)	0.0240		
number of CD20	0.39 (0.18–0.89)	0.0242		
density of CD20	0.39 (0.18–0.89)	0.0242		
number of CD20 in Stroma	0.39 (0.18–0.89)	0.0242		
number of CD20 in center	0.39 (0.17–0.89)	0.0244	0.39 (0.13–1.16)	0.0898
distance from CD20 to CD4 in Stroma	0.39 (0.17–0.89)	0.0254		
distance from CD8 to CD8	2.50 (1.11–5.63)	0.0266		
number of CD8 in center	0.40 (0.17–0.91)	0.0283	0.38 (0.13–1.10)	0.0744
distance from CK to CD68 in Tumor	0.31 (0.11–0.89)	0.0296		
density of CD8 in Tumor in center	0.40 (0.18–0.91)	0.0298	0.62 (0.22–1.75)	0.3686
density of CD4 in Stroma in center*	0.41 (0.18–0.92)	0.0317	0.42 (0.14–1.25)	0.1203
distance from CD8 to CD68 in Stroma in center	0.27 (0.08–0.90)	0.0324	0.39 (0.12–1.22)	0.1063
distance from CK to CD68	0.35 (0.14–0.92)	0.0332		
distance from CK to CD68 in Tumor in center	0.29 (0.09–0.91)	0.0345	0.53 (0.19–1.49)	0.2289
number of CK in Stroma in center	2.50 (1.07–5.84)	0.0348	2.18 (0.74–6.38)	0.1567
number of CD20 in Stroma in border	0.42 (0.19–0.94)	0.0355		
number of CD4 in center	0.42 (0.18–0.95)	0.0368	0.41 (0.14–1.20)	0.1046
density of CD4 in center	0.42 (0.18–0.95)	0.0368	0.39 (0.13–1.16)	0.0898
distance from CD8 to CD20	0.40 (0.17–0.95)	0.0370		
distance from CK to CD68 in center*	0.34 (0.12–0.95)	0.0393	0.50 (0.17–1.47)	0.2092
distance from CD20 to CD8 in Stroma in border	0.43 (0.19–0.96)	0.0404		
distance from CD20 to CK in border	0.42 (0.19–0.97)	0.0410		
number of CD20 in border	0.43 (0.19–0.97)	0.0421		
distance from CK to CD68 in border	0.37 (0.14–0.97)	0.0433		
distance from CD20 to CD20	2.33 (1.02–5.32)	0.0438		
distance from CD68 to CD68 in center*	2.89 (1.03–8.12)	0.0442	1.65 (0.59–4.63)	0.3441
distance from CD20 to CD20 in border	2.33 (1.02–5.32)	0.0450		
distance from CD68 to CD68 in Tumor in center	3.12 (1.01–9.68)	0.0489	0.63 (0.22–1.77)	0.3776

Complete list of parameters (*n* = 68) quantified using TME-Analyzer that are statistically associated with overall survival according to the categorical Cox’s proportional hazard model with median split using the discovery cohort. Hazard ratio (HR) with 95% confidence intervals (95% CI) and statistical significances in discovery cohort for all parameters, and in validation cohort for center parameters are shown. Parameters that were significant in both cohorts are shown in bold.

*indicates parameters that were top ranked according to the nested approach.

Taking these parameters and inter-relationships together, it is noteworthy that smaller tumor compartments, higher densities of CD4 and CD8 T cells, and shorter distances among CD4 T cells, between CD8 and CD56 cells and among CD68 cells were all positively associated with longer survival, whereas shorter distances between CK cells and CD68 cells, CD4 T cells, or other CK cells were negatively associated with longer survival.

## Discussion

In the current study, we have developed the TME-Analyzer, a novel tool that captures intra- and inter-tissue heterogeneity with individualized and flow cytometry-like analysis. With TME-Analyzer, we have demonstrated easy retrieval of contextures of immune effector cells in TNBC tissues from multiplexed immunofluorescent images, including cell densities and inter-cellular distances in border and center regions of either tumor or stroma compartments. We benchmarked the TME-Analyzer against two established software tools, namely inForm and QuPath, and showed high concordance regarding tissue segmentation and cell phenotyping, with enhanced accuracy and utility. Subsequently, starting from hundreds of contexture-based parameters extracted with TME-Analyzer, we have built a 10-parameter classifier for survival of TNBC patients using multiplexed immunofluorescence images, which we validated using mass spectrometry images. This 10-parameter classifier pointed to the diverse applicability of the TME-Analyzer, and also revealed the impact of inter-immune cell distances towards survival of TNBC patients as well as its outperformance of recognized prognostic parameters, such as inflammation and stromal T cell densities.

Tumors and their microscopic images are highly heterogeneous due to contextual variations in tissues and their regions, clinical variantions among patients, as well as technical variations in staining and imaging across laboratories. In order to extract data from various images in a uniform and high-throughput manner, we developed a real-time interactive analysis with a WYSIWYG interface. TME-Analyzer proved to be an easy-to-use, fast and reproducible analysis tool (Fig. 1B, Table 1), which enables exportation, sharing and standardization of analysis. With its modular analysis approach, it particularly provides versatility regarding extraction of image data from different platforms. In contrast to commercial benchmark software inForm, TME-Analyzer was more robust, i.e., requiring less algorithms for the same analysis, and efficient, i.e., able to analyze more images in the same time frame with less effort. In support of the above, TME-Analyzer uses a single algorithm rather than different algorithms for images with higher background signal, where the latter (as is the case for inForm) may yield incorrect image quantifications (Supplementary Table 4).

The utility of TME-Analyzer was also favorable when compared to QuPath. This was due to two unique characteristics of TME-Analyzer. First, in TME-Analyzer, the analysis is saved for each image whereas for QuPath the analysis is saved per project. The former enables easy application of the analysis of a single image to a multitude of images, and easy modification of analysis parameters, as needed, per image. Consequently, TME-Analyzer but not QuPath analysis performed with MxIF images can easily be extended to MIBI-TOF images with minimal adjustments. Second, TME-Analyzer has a built-in image filtering module, while QuPath comes with a set of pre-defined filters only for pixel classification (e.g. tumor and stroma compartments), but not for cell-classification (see also Supplementary Table 4). Filtering for cell classification, however, is essential to compensate for microscopy artifacts prior to cell/nucleus intensity-based phenotyping. In TME-Analyzer this filtering module is used to perform size-based background correction, which enables the usage of a single algorithm with a single set of threshold values for all images. While random forest-based cell classification in QuPath yielded comparable results (Fig. 4), the machine learning analysis may remain difficult to interpret and modify.

Furthermore, when analyzing MIBI-TOF images, we observed that the quantification of cell abundance, density and interspacing according to the TME-Analyzer was in agreement with DeepCell segmentation and other algorithms originally used to analyze the MIBI-TOF data set^11^. DeepCell has been specifically trained for the MIBI-TOF dataset that was used for validation purposes, and as a consequence performs better than StarDist in case of this dataset. StarDist, which is integrated in TME-Analyzer, has been specifically trained for immunofluorescence images^20^. While the shape of the segmented nuclei is clearly different between both methods, the number of cells and their relative distribution among cell phenotypes remain highly concordant. The latter observation is in agreement with recent work where random patch-based interrogation of tumor microenvironments was able to extract clinical relevance without any information of cell locations^24^. Indeed, we were able to demonstrate that TME-Analyzer performs well in analyzing images coming from either immunofluorescence or MIBI-TOF platforms. Moreover, TME-Analyzer is a modular tool enabling the option, when preferred, to incorporate any segmentation method or segmentations themselves from other methods. In addition to the TNBC analysis presented here, TME-Analyzer can interrogate multispectral images obtained for various different tumor pathologies, e.g., head and neck squamous-cell carcinoma^25^, metastatic urothelial carcinoma^3,26^, and glioma (non-published data).

The composition of the micro-environment is of clinical value for various tumor types, including TNBC. For instance, the clinical value of the presence and location of tumor-infiltrating lymphocytes (TILs) has been well established for TNBC^2,27–29^. With the TME-Analyzer, we indeed observed higher densities of B cells, CD4 T cells, CD8 T cells and tumor associated macrophages (TAMs) in the center of inflamed but not non-inflamed tumors, being in line with previous reports using Mx-IF and IHC imaging^2,30^. Additionally, and as expected, phenotyped cells showed shorter distances to CD8 T cells in inflamed vs non-inflamed tumors.

Besides the benchmarking of TME-Analyzer, we assessed whether TME-Analyzer was able to discriminate between patients with short- and long-term survival. To test this, we first identified maximally uncorrelated parameters and ranked them based on their prognostic potential. We then obtained a classifier using the top ten parameters. This approach applied to the validation cohort was indeed able to distinguish between patients with short- and long-term survivor. Interestingly, when testing different classification stringencies, we observed that the inclusion of the top six parameters or more could be validated. Additionally, 10 parameter classifiers obtained from a subset of the discovery cohort patients (16, 32 or 47 patients instead of full 63 patient cohort) could still be validated (data not shown). These two findings further demonstrate the robustness of our approach and justifying the selection of the top 10 parameters. Here, the parameter deconvolution to identify maximally uncorrelated parameters was a crucial step that has not been generally applied to multiplex images. While this approach has analogies in radiomics analysis^31^, dimensionality reduction in multiplex imaging data has been towards cell phenotyping and patient identification^11,32,33^, and not parameter decorrelation. Our classification approach also outperformed patient segregation based on T cell spatial phenotype from the pathological reports and T cell density quantified by TME-Analyzer, which are known prognosticators for TNBC^2,11,34^. When applied to an independent cohort, only the classifier retained its prognostic value. Furthermore, the classifier was also able to classify patients with survival less than 5 years with a sensitivity of 100% and a specificity of 85%. This was despite the completely different tissue processing and microscopy methods used in the two cohorts. While machine learning classifiers like random forest might further improve the performance, the averaging approach as followed in our study already yielded high accuracy. Our approach, in contrast to other classifiers, requires less computational time and allowed for easy interpretation of parameter values. The image analysis performed with TME-Analyzer with only slight adjustments between cohorts not only showed agreement with dedicated analysis, but also allowed identification of prognostic parameters. This demonstrates the robustness of TME-Analyzer and validates it as a versatile tool for accurately extracting information from tumor microenvironments.

While our aim was to establish the TME-Analyzer as a tool for extracting immune contextures from images, one of the outcomes of our approach was the ranking of these parameters. The presented approach appeared more sensitive than the conventional Cox analysis, as according to Cox analysis only 6 out of 10 parameters were associated with survival, and only for the discovery cohort (Table 2). One argument potentially explaining the outperformance of our approach compared to other approaches, such as the Cox model, is the ability of our approach to overcome sampling bias. The Cox model depends on the composition of patients in a given cohort, whereas in the presented Monte Carlo approach small subsets of patients were re-sampled for each round of repetition. While this allows for limited sample size per round, repetition of this selection 1000 times enables enhanced statistical power. In the current example, given that there are about 3 × 10^12^ different 12-patient subsets (constituting shorter and longer survivors per round) of a 63-patient cohort, the 1000 subsets we analyzed during the 1000 repetitions effectively represent independent patient cohorts. Of the top 10 ranked contextual parameters observed for longer survivors, 1 parameter was large tumor area, 3 related to high densities of T cell subsets in different compartments, 3 to shorter distances among CD4 T cells, among TAMs and between NK and CD8 T cells, and 3 to longer distances between tumor cells and either TAMs, CD4 T cells, and other tumor cells (Fig. 5B). Notably, the majority of the parameters relate to intercellular distances, which underlines that besides the mere presence and location of CD8 T cells, intercellular distances among immune effector cells and/or tumor cells impact survival of TNBC patients.

Interestingly, larger areas of tumor correlated with longer distances among tumor cells, which in turn may be related to reduced tumor cell aggregation, less small islands of tumor cells or tumor cells that are smaller in size, resulting in longer inter-cellular distances among tumor cells in long-lived when compared to short-lived patients. Furthermore, many prognostic parameters were strongly and positively interrelated, namely densities of CD4 T cells in tumor and stroma, density of CD8 T cells in stroma and distance from tumor cells to CD4 T cells. These parameters were negatively interrelated with distance among CD4 T cells and among TAMs. These findings extend earlier reports that point to the prognostic value of numbers and spatial orientation of these immune effector cells in TNBC^2,11,34–37^. Strikingly, most of the parameters from the classifier (6 out of 10) relate to cellular distances. In fact, for longer survivors, shorter distances were observed among TAMs and CD4 T cells, as well as between NK and CD8 T cells. The shorter distances may point to the presence of immune cell clusters that contain CD4 and CD8 T cells, TAMs and NK cells. It is of interest that particular CD4 T cell helper subsets might be critical components of such immune cell clusters, and may determine tumor evolution and responsiveness to immune checkpoint inhibitors. This would be in line with previous reports where we showed the presence of T follicular helper (T_fh_) or T helper type-1 (T_h1_) cells in clusters found in either metastatic urothelial cancer or oral cavity cancer and their prognostic and predictive value, respectively^3,25^. Within these clusters, the presence of immune effector cells, such as NK cell and CD8 T cells, may ultimately facilitate anti-tumor responses^38–41^. Shorter distances observed among TAMs in longer survivors may suggest the presence of M1-like TAMs that may further support the anti-tumor activity of T cells^42,43^. Together, our findings in long-lived patients are in line with the presence of immune cell clusters reminiscent of tertiary lymphoid structures that have been reported to aid anti-tumor T cell responses. Specifically, the presence of tertiary lymphoid structures where immune cells cluster has been related to better clinical outcome in various solid tumors^44^, including TNBC^11^, ovarian cancer^45^, lung cancer^46^, metastatic urothelial cancer^3^ and oral cavity cancer^1,25^.

The lower abundance and reduced clustering of immune effector cells in short-lived TNBC patients may point to active suppression of an anti-tumor T cell response. The non-inflamed TNBC can be subdivided into excluded and ignored phenotypes, each with unique features, regarding the involvement of extracellular matrix components and myeloid cells, respectively^2^. Tumor cell pathways, as for instance VEGF/TGFβ and WNT pathways^2,47–49^, may direct such features, and together with smaller tumor islands, dominate the suppressive TME in TNBC in shorter survivors. The beneficial self-clustering of macrophages, as discussed above for long-lived patients, might be dominated by tumor suppressive M1-like macrophages, the detrimental clustering of tumor cells and TAMs as observed for short-lived patients may be dominated by tumor promoting M2-like macrophages^2,50–53^. In fact, recent reports demonstrated that an M2-like gene signature^2^ and a higher TAM to T cell ratio^30^ are associated with worse prognosis, while not finding a direct relationship between abundance of TAMs and survival^2,30^, and argue that further studies are needed to define the exact composition of TAMs in relation to their contextual prognostic value in TNBC. Similarly, detrimental clustering of tumor cells and CD4 T cells as observed for short-lived patients could be due to CD4 T cells being predominantly composed of regulatory T cells (T_reg_) that are able to keep the activity of CD8 T cells in check^54,55^. The higher abundance of M2-like TAMs and/or Tregs would be in line with a micro-milieu in poor survivors that facilitates polarization of myeloid and T cells towards a suppressive phenotype.

One limitation of our study is the likely difference between the discovery and validation cohorts when it comes to stage and treatment of TNBC. While the tissues from the discovery cohort came from treatment-naïve TNBC patients, tissues from the validation cohort also came from patients with TNBC recurrence following treatment. Additionally, following sampling, patients from both cohorts were subjected to different follow-up treatments. Therefore, the testing of the contextual classifier in larger TNBC patient cohorts, taking into account newer treatment modalities, such as anti-PD1 and sacituzumab govetican, would aid the translation of our findings towards clinical usage at the level of patient selection. Such clinical application would also benefit from further reduction in the number of markers studied to make it compatible with for instance a more standard immunofluorescence setup of 4 markers. Another limitation is the lack of characterization of TAM and CD4 T cell subsets. Further studies may include markers of such subsets, which could enhance our understanding and the development of possible interventions. A final limitation relates to the TME-Analyzer software, which is an.exe file that can be run on Windows computers, where images are read in their entirety into the RAM. The users with different operating systems can make use of the provided Python code instead, whereas its usage with large images and/or limited RAM is advised to be implemented with tiled analysis. Here, in addition to the available RAM limiting the size of the image that can be analyzed, an additional constraint lies with the StarDist module, which can handle images of sizes up to approximately 4 M pixels. It should be noted that the image analysis field is highly dynamic with a continuous development of software tools both commercial (e.g., HALO, Indica Labs, USA) and open-source (e.g., the community support of QuPath). Moreover, in addition to random forest, artificial neural network and K nearest neighbor based machine learning cell phenotyping algorithms often incorporated into new software tools, high accuracy has also recently been reported for convoluted neural network based phenotyping from multiplex images^56^.

In conclusion, the TME-Analyzer captures the heterogeneity of the immune microenvironment of patient’s tumors with a new WYSIWYG interface and real-time interactive analysis. This tool enables extraction and analysis of data from multiple platforms in a uniform, high-throughput and interactive manner, and showed high concordance with two benchmark software tools. TME-Analyzer demonstrated enhanced accuracy as well as utility regarding immune phenotyping. Notably, TME-Analyzer demonstrated its value in building a contextual classifier for survival of TNBC patients. Besides showing the diverse applicability of this software, our findings revealed the impact of inter-immune cell distances towards survival of TNBC patients as well as its outperformance of recognized prognostic parameters, such as inflammation and stromal T cell densities. TME-Analyzer and the clinical analysis we present here can be easily and quickly applied to large cohorts of patients irrespective of imaging modality used, making it a powerful tool for studying TME spatial architecture.

## Methods

### Cohorts of patients

#### Testing and discovery cohort

MxIF images of a historic cohort of 63 primary TNBC patients^2^ with node-negative tumors were used to test the TME-Analyzer as well as to identify a prognostic classifier. Complete clinicopathological records were available with >10 year follow up (see^2^ for clinical details).

#### Validation cohort

MIBI-TOF images and clinical data from a cohort of 41 TNBC patients^11^ were used as an independent cohort to confirm the performance of the prognostic classifier. The images were obtained from a tumor tissue microarray with cores obtained from center regions, thus excluding border regions. As for one of the patients 2 cores were included in the dataset (#15 and #22), the latter core was excluded from analysis, whereas for another patient clinical information was not provided (#38), and this patient was also excluded from our analysis, resulting in an actual cohort of 39 patients. A TIL score was also reported for a subset of this patient cohort (*n* = 25, see^11^).

### Multiplexed immunofluorescence staining and scanning

Formalin-fixed paraffin-embedded resection materials of discovery cohort samples were multiplexed stained for immune effector cells and imaged at border and center regions. MxIF was performed with OPAL reagents (Akoya Biosciences) using whole tissue sections as reported before^2^. In brief, stainings included multiple cycles of antigen retrieval followed by cooling, blocking, and consecutive staining with primary antibodies, HRP-polymer and Opal fluorophores. These cycles were repeated until all markers were stained. Finally, nuclei were stained with DAPI. Whole section scans were obtained using VECTRA 3.0 (Akoya Biosciences) with at least 8 stamps (i.e., regions of interest) that were set in non-necrotic areas at the tumor border and center (see^2^ for details). Spectral unmixing was performed using inForm (v2.4.1). The following antibodies were used in order of staining with the indicated titres; 1. CD56 (MRQ-42, Sanbio, 1:500) – OPAL620; 2. CD3 (SP7, Sigma, 1:350) – OPAL520; 3. CD20 (L26, Sanbio, 1:1000) – OPAL650; 4. CD8 (C8/144b, Sanbio, 1:250) – OPAL570; 5. CD68 (KP-1, Sanbio, 1:250) – OPAL540; 6. Cytokeratin-Pan (AE1/AE3, Thermofisher, 1:200) – OPAL690; 7. DAPI.

### Image analysis with TME-analyzer

TME-Analyzer software is deposited at www.tme-facility.com/tme-analyzer and the source code is available at https://github.com/ErasmusMC-Bioinformatics/TME_Analyzer. Analysis with TME- Analyzer was performed in the following steps: (i) Scanned MxIF images were loaded onto the TME-Analyzer (exemplified in Fig. 1A.i). (ii) Images were subjected to foreground detection (Fig. 1A.ii), where foreground regions consisted of areas with signals that are higher than 0.1 in any channel following background correction. The background correction was a result of subtracting the image that was uniformly filtered with a size of 1000-px from the original image. To accurately detect foreground in this step, small gaps were subsequently removed using a “gap-filling” algorithm, to which end small isolated regions (areas < 5000 px) were assigned to the enclosing background or foreground. (iii) Tumor and stroma compartments were defined (Fig. 1A.iii), where signals higher and lower than 1.0 in smoothened and background-corrected cytokeratin images were assigned to tumor and stroma, respectively. Smoothing was performed using a gaussian blur filter (size: 10-px), and subsequently background correction and gap-filling were performed on the smoothened image as described in step ii. (iv) Nucleus**/**cell segmentation was obtained using the well-established methods StarDist and Voronoi. StarDist uses an artificial network to determine cell nuclei as star-convex polygons^20^, whereas Voronoi segmentation assigns regions to the closest seed locations^21^. In this step, TME-Analyzer first detects single nuclei through the StarDist algorithm “2D_versatile_fluo”^20^ using the DAPI channel, after which it extracts individual cell areas from the foreground image through Voronoi segmentation according to the center locations of single nuclei as seeds (Fig. 1A.iv). (v) Cells were assigned as marker-positive based on the mean fluorescence of the marker(s) of interest obtained from the background-corrected image from step ii. Specifically, cells were assigned as marker-positive in the regions of interest, when cells had a mean intensity higher than 0.1, 0.3, 0.4, 0.7, 0.7, and 1.0 for the CK, CD3, CD8, CD20, CD56 and CD68 channels, respectively (Fig. 1A.v). Table 1 lists all analysis parameters, which were obtained through manual analysis of representative images of 10 patients. The analysis using TME-Analyzer with parameters set as described above took <30 s per 8-channel image of 1340 × 1004 pixels on a device with Intel(R) Core(TM) i9–10850K CPU @ 3.60 GHz 3.50 GHz CPU and 16 Gb RAM, without GPU usage.

Image analysis of MIBI-TOF images was similar to that of MxIF images (see Table 1 for analysis parameters used for both image platforms). Particular differences were as follows. For foreground detection, images required signals higher than 0 in any channel after background correction. For removing tissue artifacts based on size, isolated regions <250 pixels were assigned to enclosing background or foreground. For the definition of tumor and stroma compartments, the methodology was identical as for MxIF, signals with mean CK intensity higher than 0.5 and area larger than 5000 pixels assigned to tumor, and regions with signal <0.5 or area <5000 px assigned to stroma. Single nuclei, single cells, and cells positive for a marker were detected as described above. Cells were assigned as marker-positive in the regions of interest, when cells had a mean marker intensity higher than 0.

Finally, phenotyped cells from MxIF or MIBI-TOF images were individually assigned to either tumor or stroma compartments based on the localization of the center of their nuclei, and annotated as follows: tumor cells: CK +; CD4 T cells: CD3 + CD8−; CD8 T cells: CD3 + CD8 +; B cells: CD20 +; NK cells: CD56+; Macrophages: CD68+, where marker positivity was assessed per individual cell in an inclusive manner for analytical purposes.

### Image analysis with benchmark software tools

#### inForm

The inForm analysis has been explained in detail before^1,2^. In brief, MxIF images were analyzed with trainable algorithms using the inForm software version 2.4.1 (Akoya) through compartment segmentation, cell segmentation and cell phenotyping. Algorithm training for compartment segmentation was based on CK and DAPI signals, and resulted in: tumor segments (CK-positive, DAPI-positive); stroma segments (CK-negative, DAPI-positive); and non-tissue segments (CK-negative, DAPI-negative). Training of the software for detection and phenotyping of marker-positive cells was performed according to manual assessment of fluorescent expression patterns and cell morphology. The cells were assigned to one of the following phenotypes: tumor cells: CK + ; CD4 T cells: CD3 + CD8-; CD8 T cells: CD8 + ; B cells: CD20 + ; NK cells: CD56 + ; Macrophages: CD68 + ; others (no clear signature).

#### QuPath

For the QuPath analysis, all images were analyzed using a single algorithm generated from 10 images using the QuPath softwave version 0.5.1^23^. In brief, images (*n* = 10) were selected from different patients and stitched together as a training image (through QuPath option Classify -> Training images -> Create training image), and subsequent analysis was carried on this image with machine learning approaches to obtain and define values for the analysis parameters. Compartment segmentation was performed by manually annotating 20+ regions, each, for tumor, stroma and non-tissue areas and training an artificial neural network on Gaussian features at 2 μm/pixel resolution with minimum object and hole size both set to 200 μm^2^. Cell segmentation was performed using the StarDist^20^ extension using the “dsb2018_heavy_augment” model. Subsequently, cell phenotyping was performed per channel by annotating at least 200 cells that were positive (with the exception of CD56 where 121 cells were annotated, as this was the total number of CD56+ cells that were detected), and 200 that were negative for the marker of interest. Furthermore, cell phenotyping was completed by training random trees machine learning model for all parameters based on the intensity of the marker of interest. The trained classifiers were then applied to all 1030 images and downstream data analysis was performed using Python (v3.11.8). Here, phenotyped cells were assigned to compartments and annotated identical to the TME-Analyzer analysis; i.e. assigned to either tumor or stroma compartments based on the localization of the center of their nuclei, and annotated as: tumor cells: CK+; CD4 T cells: CD3+ CD8−; CD8 T cells: CD3 + CD8+; B cells: CD20+; NK cells: CD56+; Macrophages: CD68+, where marker positivity was assessed per individual cell in an inclusive manner for analytical purposes.

### Inflammation status of TNBC

Patients in the discovery cohort (*n* = 63) were split into 2 groups; inflamed (*n* = 25) and non-inflamed (*n* = 38) based on median CD8 T cell density at border and center as reported previously^2^. Briefly, a TNBC sample was annotated as inflamed if there were >200 cells/mm^2^ CD8 T cells at the border region, and the ratio between border and center regions was <10. TNBC samples that failed to achieve these criteria were characterized as non-inflamed. For patients in the validation cohort, the TIL-score reported for 25 patients was used^11^.

### Quantification of numbers of immune effector cells, their densities and intercellular distances

The determination of numbers and densities of phenotyped immune cells as well as distances between these cells in any tissue compartment or region were performed using Python (v3.8.10). Phenotyped cells assigned to individual regions based on the location of their nuclei were enumerated and their densities were calculated by dividing the number of cells with a certain phenotype by the total area of a given region. Distances were calculated as Euclidian distances and converted to z-scores to account for its dependence on cell density, with negative values indicating a higher chance of clustering of cells compared to random chance, as previously described^11^. In brief, for each image, distances were calculated for all pairs of cellular phenotypes. From this matrix, the minimal non-zero distance from one phenotype of cells to another (i.e., nearest neighbor distance), in a given tissue compartment or region, was calculated, and averaged for all pairs of cellular phenotypes. To normalize the data, the number that corresponds to the cells with the first phenotype was selected at random within the tissue compartment or region of interest, and this was repeated for the second phenotype (unless it was a “self-distance”, e.g. B cell to B cell, in which case, the same random selection was kept for both phenotypes). These random selections and averaging of the minimal non-zero distance between these random phenotypes were repeated 1000 times, and the mean minimal distance as well as its standard deviation was recorded. The distance z-score was calculated from the actual cellular minimal distance by subtracting the mean and dividing by the standard deviation of the minimal distance obtained from the random repetition. Positive (and negative) distance z-scores indicated larger (and smaller) distances between given cell types compared to random distances, where the values were measured in standard deviations (with absolute values exceeding 2 indicating statistical significance).

### Building contextual classifiers

#### Ranking of parameters according to their prognostic value

To obtain a set of independent parameters that can be tested on the validation cohort, only center images were used for the classifier to enable proper confirmation using our validation cohort^11^ (consisting only of images taken at tumor core, see above). 50 center parameters, namely: densities of immune cell populations in tumor and stroma compartments (*n* = 12); areas of these compartments (*n* = 2); and distance *z*-scores between phenotypes in whole tissue (*n* = 36), were ranked with a re-iterative nested Monte Carlo approach as described below.

The outer loop was the building and testing of classifiers (see Fig. 5A, blue frame). First, we have split the patient dataset (*n* = 63) randomly into a train (*n* = 31) and test (*n* = 32) set in such a manner that patients in both sets had similar survival rates, i.e., patients with shorter and longer than median survival were equally split between the 2 sets and survival was not significantly different between the sets (*p* > 0.05 according to log-rank test). Second, we have ranked the 50 parameters using the train set. To this end, interim classifiers were constructed starting from the 50 parameters by re-iterating the following 4 steps (these constitute the inner loop, see Fig. 5A, orange box): (1) random selection of 12 patients; (2) splitting these 12 patients for each parameter into 2 groups of 6 patients according to the median value of parameters; (3) testing statistical significance of survival differences between the 2 patient groups per parameter according to log-rank tests; and finally (4) upon significance, recording the mean values per parameter for the shorter and longer survival groups. After 1000 repetitions, the means of statistically significant parameters were calculated for shorter and longer survival groups. Third, we ranked the parameters based on the degree of separation (*p*-value) between the survival groups obtained in the train set. Per parameter, the train set was split into “better” and “worse” prognosis groups according to the parameter average in a given patient being closer to the “better” or “worse” mean parameter value. Fourth, we obtained interim classifiers, with which patients were assigned either into bad or good prognosis groups according to the majority of parameters being closer to the recorded means of shorter or longer survivors, respectively. These interim classifiers were fine-tuned until statistically significant performance was reached in the train set, by re-iteratively excluding the worst performing parameters. Fifth, the surviving interim classifier (i.e., the resulting set of parameters and their recorded means) was tested in the test set (*n* = 32). In the case when statistical significance was also observed for the test set, then the set of parameters that defined the classifier were considered to have a first hit. The above 5 layers are repeated 1000 times (the interim classifiers and their outcome are provided as supplementary data), and the total number of hits of all 50 parameters is recorded. Lastly, the 50 parameters were ranked based on the total number of hits in the test set, and the top-10 parameters were taken along for building the final classifier as explained below.

#### Constructing the classifier

Using a number of top parameters, classifiers were built using data from all 63 patients of the discovery cohort following the inner-loop procedure described above (with 4 steps). These steps were again repeated 1000 times, and subsequently parameters were identified that showed a statistically significant difference according to log-rank test in survival upon median-split, which constituted the final classifiers. For these parameters the mean values were recorded and enabled the splitting of the patient cohorts according to values being either closer to the shorter or longer survival means per parameter. The classifier that consists of the top-10 parameters was further investigated (shown in Fig. 5B), whereas Kaplan-Meier curves of the other classifiers are available as supplementary data.

#### Inflammation status, TIL score and stromal T cell density

Patients in the discovery cohort (*n* = 63) were also split into 2 groups according to inflammation status (inflamed: *n* = 25; and non-inflamed: *n* = 38; see above for details). In addition, patients in the validation cohort (*n* = 39) were split into 2 groups according to TIL score as this score is reported for 25 out of 39 patients of this cohort. A median split was applied to this TIL score (high: *n* = 12; low: *n* = 13). Thirdly, patients from both cohorts were split into 2 groups according to median of combined densities of CD4 and CD8 T cells in the stroma compartment of the center region (high: *n* = 31 for discovery and *n* = 19 for validation; low: *n* = 32 for discovery and *n* = 20 for validation).

### Statistics

For cellular and spatial parameters, the concordance between TME-Analyzer and published analysis^2,11^ was assessed with linear relation (scipy.stats.linregress, *v* = 1.10.1), Pearson correlation and Wald Test with t-distribution of the test statistic. The correlations between various cell metrics, calculated using output from TME-Analyzer, were assessed with Spearman correlation (scipy.stats.spearmanr). Comparisons between inflamed and non-inflamed quantifications were carried using Mann–Whitney *U* test (scipy.stats.mannwhitneyu).

For clinical parameters, lifelines (v0.27.8) package in Python was used to determine the relationship between cell metrics and overall survival (OS). Univariate and multivariate Cox analysis was performed non-parametrically using Breslow’s method. Kaplan–Meier curves were used to visualize the survival of individual groups. Survival differences between 2 groups of patients (split according to classifier, T cell infiltration, inflammation) were quantified with log-rank tests. *P*-values < 0.05 were considered significant.

## Supplementary information

Supplementary material

interim classifiers

Sample images, analysis tutorial and a sample analysis

Supplementary table 1

Supplementary table 2

Supplementary table 3

Supplementary table 5

Supplementary table 6

Supplementary table 7

varying parameter classifier performance

## Data Availability

Processed imaging data of individual images and patients of the discovery and validation cohorts are presented in Supplementary Tables 1–3 and 5. 8 sample images, analysis tutorial and an analysis file from the discovery cohort is available with this manuscript, and the software comes with the option of loading the analysis parameters for the discovery cohort. No new imaging was carried on in this manuscript. Any further requests for raw imaging data should be addressed to the corresponding authors of respective manuscripts^2,11^. Software is available at www.tme-facility.com/tme-analyzer. Python source code is available at https://github.com/ErasmusMC-Bioinformatics/TME_Analyzer.
