# Supplementary material for: TME-analyzer: a new interactive and dynamic image analysis tool that identified immune cell distances as predictors for survival of triple negative breast cancer patients
Source: Npj Imaging. 2024 Jul 25;2:21. doi: 10.1038/s44303-024-00022-6 (PMC12118654; doi:10.1038/s44303-024-00022-6)
Supplement: Supplementary file 1 — Supplementary material [file 44303_2024_22_MOESM1_ESM.pdf]

Supplementary material

TME-Analyzer: a new interactive and dynamic image analysis tool that identified immune cell distances as predictors for survival of triple negative breast cancer patients

Hayri E Balcioglu[1*], Rebecca Wijers[1], Marcel Smid[2], Dora Hammerl[1], Anita M Trapman-Jansen[2], Astrid Oostvogels[1], Mieke Timmermans[2], John WM Martens[2], Reno Debets[1*]
Laboratories of [1]Tumor Immunology and [2]Translational Cancer Genomics, Department of Medical Oncology, Erasmus MC Cancer Institute, Rotterdam, The Netherlands
[*]*correspondence: Hayri E Balcioglu, h.balcioglu@erasmusmc.nl; Reno Debets, j.debets@erasmusmc.nl*

Supplementary table 1. TME-Analyzer's contextual outcomes of images for discovery cohort.

Areas of stroma and tumor compartments; as well as numbers, densities, intercellular distances, and distance z-scores of CD4, CD8, CD20, CD56, CD68 and CK in these compartments quantified with TME-Analyzer, and presented per image for the discovery cohort.

Supplementary table 2. TME-Analyzer's contextual outcomes averaged per patient for discovery cohort.

Areas of stroma and tumor compartments; as well as numbers, densities, intercellular distances, and distance z-scores of CD4, CD8, CD20, CD56, CD68 and CK in these compartments quantified with TME-Analyzer, and averaged per patient across border, center and all regions for the discovery cohort.

Supplementary table 3. inForm's contextual outcomes of images for discovery cohort.

Areas of stroma and tumor compartments; as well as numbers, densities and intercellular distances of CD4, CD8, CD20, CD56, CD68 and CK in these compartments quantified with inForm, and presented per image for the discovery cohort.

Supplementary table 4. Algorithms and enhanced utility of TME-Analyzer in comparison to the benchmark software tools.

Side-by-side comparison between TME-Analyzer, inForm and QuPath, indicating algorithms used for different steps of analysis, and a short list of unique characteristics of all platforms.

Supplementary table 5. TME-Analyzer's contextual outcomes of images for validation cohort.

Areas of stroma and tumor compartments; as well as numbers, densities, intercellular distances, and distance z-scores of CD4, CD8, CD20, CD56, CD68 and CK in these compartments quantified with TME-Analyzer, and presented per image for the validation cohort.

Supplementary table 6. Listing of the 50 tested parameters and their classifier values

Complete ranking of tested parameters (n=50) with their percentage occurrence according to nested approach and their Monte-Carlo classifier means and standard deviations obtained in the discovery cohort.

Supplementary table 7. Performance of Monte-Carlo classifiers that were built with varying number of parameters

The performance results of any top number of parameters presented in Supplementary table 6 in the discovery and validation cohorts with p-values of survival comparison obtained using log-rank test.

Deposited data:

Composition and performance of interim classifiers

.zip folder containing; a .csv file listing the classifier parameters; a .txt file listing the patient data used for test-train groups and the classifier performance; and a .png file plotting the Kaplan-Meier curves of patient groups per classifier for 1,000 interim classifiers.

Performance of Monte-Carlo classifiers of top parameters

.zip folder presenting the Kaplan Meier curves for different classifications for discovery and validation cohorts of the classifiers presented in Supplementary table 7.

Sample images, analysis tutorial and a sample analysis

.zip folder containing 8 sample images from the discovery cohort, analysis tutorial, and a sample analysis.

All Supplementary tables, with the exception of Supplementary table 4 (below), are provided as separate excel files.

Supplementary table 4. Algorithms and enhanced utility of TME-Analyzer in comparison to the benchmark software tools.

	**TME-Analyzer**	**inForm**	**QuPath**
**Algorithms:**			
Compartment segmentation	Manual	Machine learning	Machine learning or manual
Cell segmentation	Machine learning or manual	Machine learning	Machine learning or manual
Phenotyping	Flow-like interactive gating	Machine learning	Machine learning or manual
Data analysis	Integrated	Requires R	Integrated
Image unmixing	Channel-crosstalk	Spectral	Not supported for fluorescence
**Unique utilities:**			
Contrast adjustment for visualization		√	√
Image filtering for cell phenotyping	√		
Interactive, and flow-like size control for tissue, cell and foreground	√		
Real-time and shape-based phenotyping of tissues and cells	√		√
Built in (spatial) data analysis	√		√
Saving of analysis parameters	Per image	Per project	Per project
Modification of analysis parameters	√		
In software scripting and ImageJ incorporation			√
Processing time per image	30 seconds	4 minutes	10 seconds

Supplementary figures

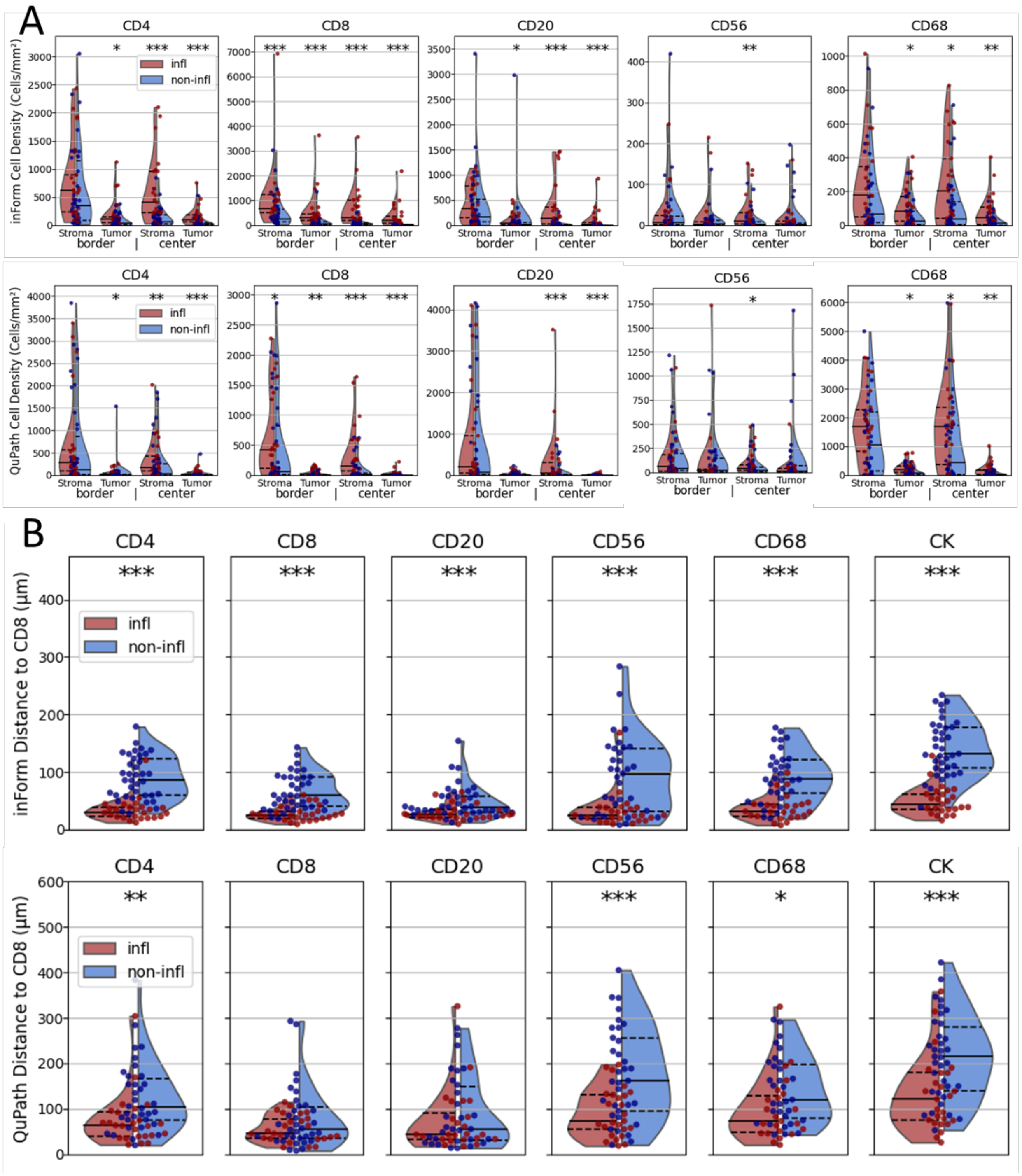

Supplementary figure 1. Annotation of inflamed and non-inflamed TNBC according to benchmark software tools. A. Violin and scatter plots of densities of different phenotypes of immune cells, together with median (solid black line) and 25% quartiles (dashed black lines), in stroma and tumor compartments of border and center regions in cells per mm^2 quantified using inForm (top) and QuPath (bottom) for inflamed (red) and non-inflamed (blue) tumors. **B.** Violin

and scatter plots of distances between different phenotypes of immune cells and CD8 T cells, together with median (solid black line) and 25% quartiles (dashed black lines), in all regions/compartments in µm quantified using inForm (top) and QuPath(bottom) for inflamed (red) and non-inflamed (blue) tumors. *: $p<0.05$, **: $p<0.01$, ***:$p<0.001$ according to Mann-Whitney U test comparing data from inflamed to non-inflamed tumors.

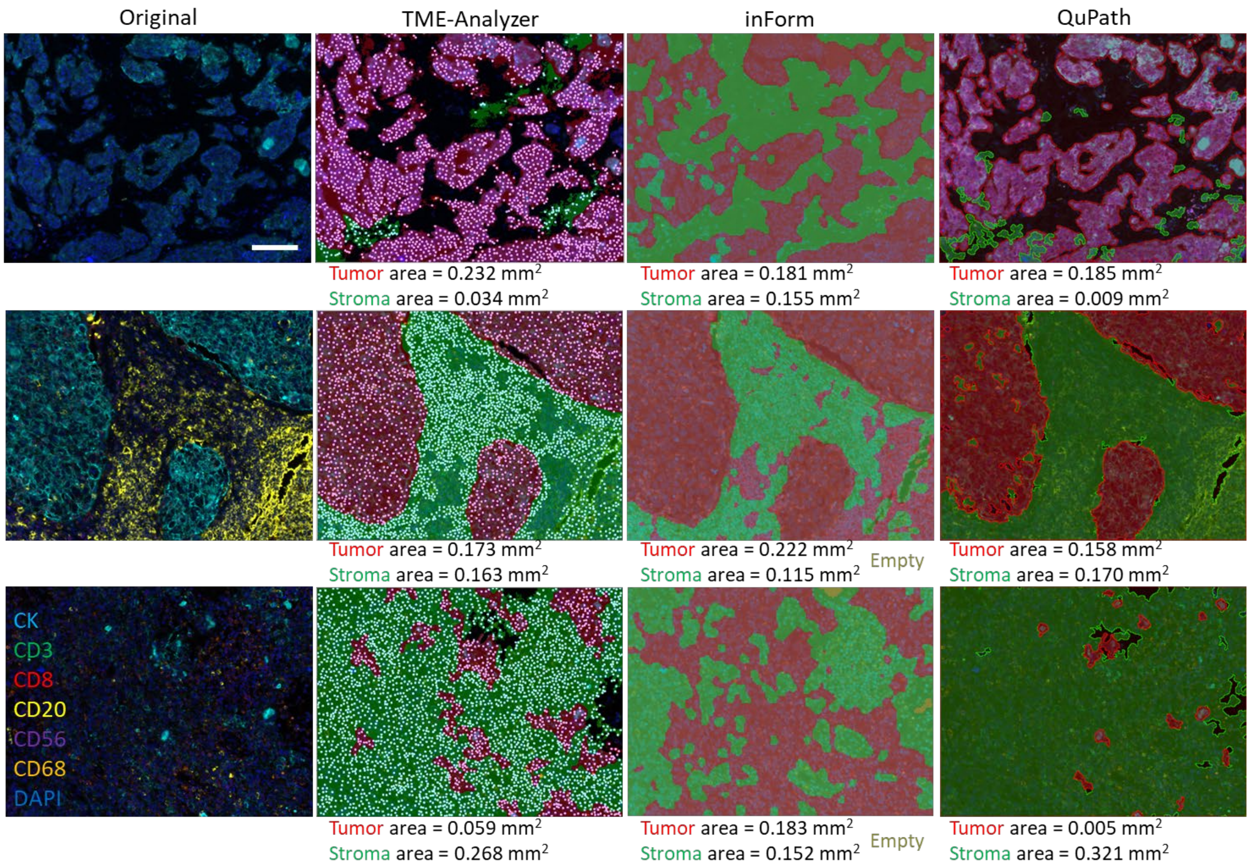

Supplementary figure 2. TME-Analyzer tissue segmentation shows improved accuracy when compared to inForm software. Representative images (1st column) and tumor (red) and stroma (green) tissue segmentation with TME-Analyzer (2nd column), inForm (3rd column) and QuPath (4th column) for images of high discordance between TME-Analyzer and inForm. Scale bar is 100 µm.

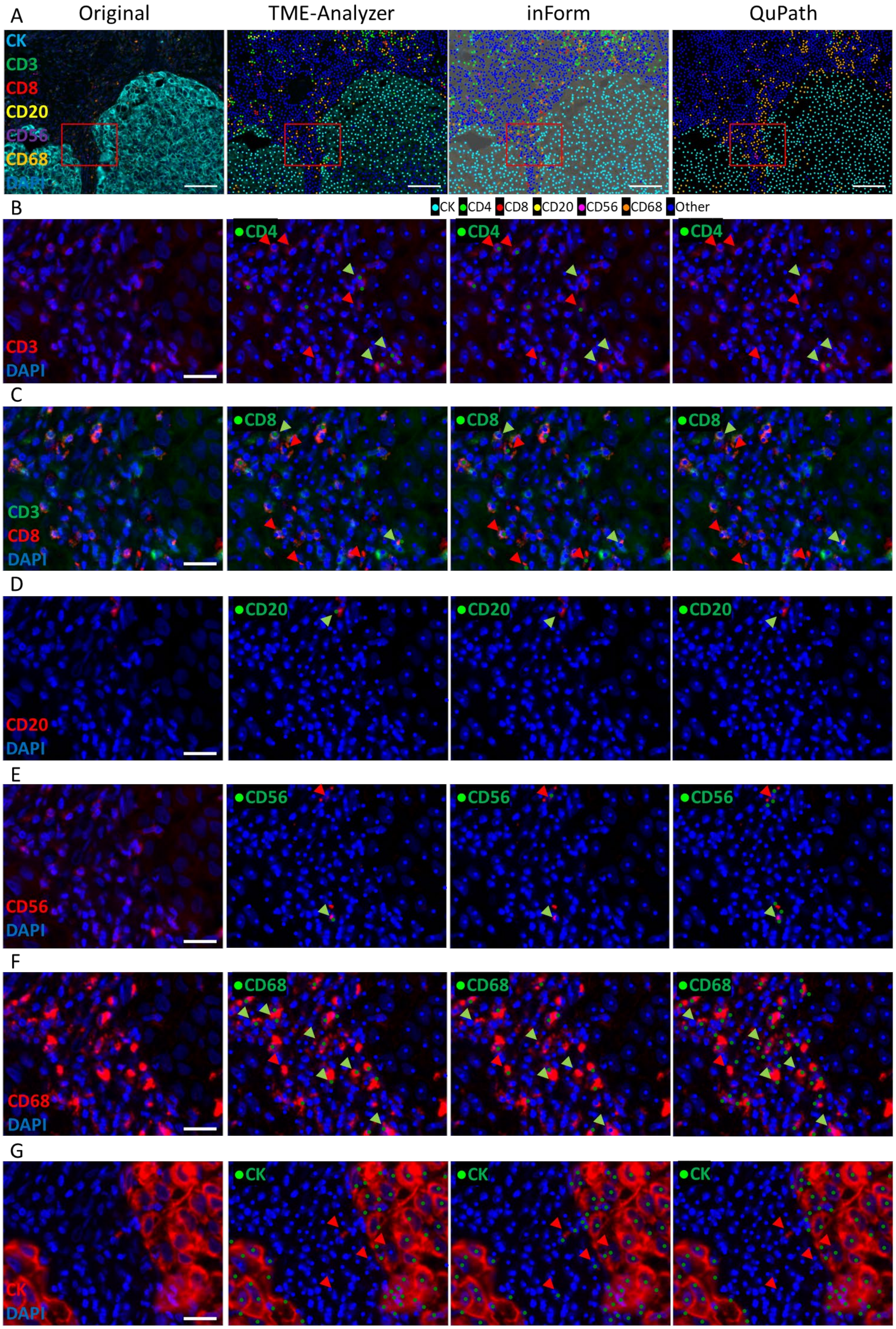
A
Original
TME-Analyzer
inForm
QuPath
CK
CD3
CD8
CD20
CD56
CD68
DAPI
B
CK
CD4
CD8
CD20
CD56
CD68
Other
CD4
CD3
DAPI
C
CD8
CD3
CD8
DAPI
D
CD20
CD20
DAPI
E
CD56
CD56
DAPI
F
CD68
CD68
DAPI
G
CK
CK
DAPI

Supplementary figure 3. Representative images of cell phenotyping from TME-Analyzer and the benchmark software tools. (A). Representative multiplexed image (1st column) and corresponding phenotyping with TME-Analzer (2nd column), inForm (3rd column) and QuPath (4th column). (**B-G**) zoom in of the red box in A with multichannel image (1st column) showing CD3 (B, red; C, green), CD8 (C, red), CD20 (D, red), CD56 (E, red), CD68 (F, red), CK (G, red) together with DAPI (B-G, blue) and phenotyping outcome of TME-Analyzer (2nd column), inForm (3rd column) and QuPath (4th column) for CD4 (B), CD8 (C), CD20 (D), CD56 (E), CD68 (F) and CK (G) shown with green dots. Red arrowheads indicate cells phenotyped according to inForm but not TME-analyzer, and green arrow heads indicate cells phenotyped by TME-Analyzer and not inForm. Scale bars are A: 100 µm, B-G: 25 µm.

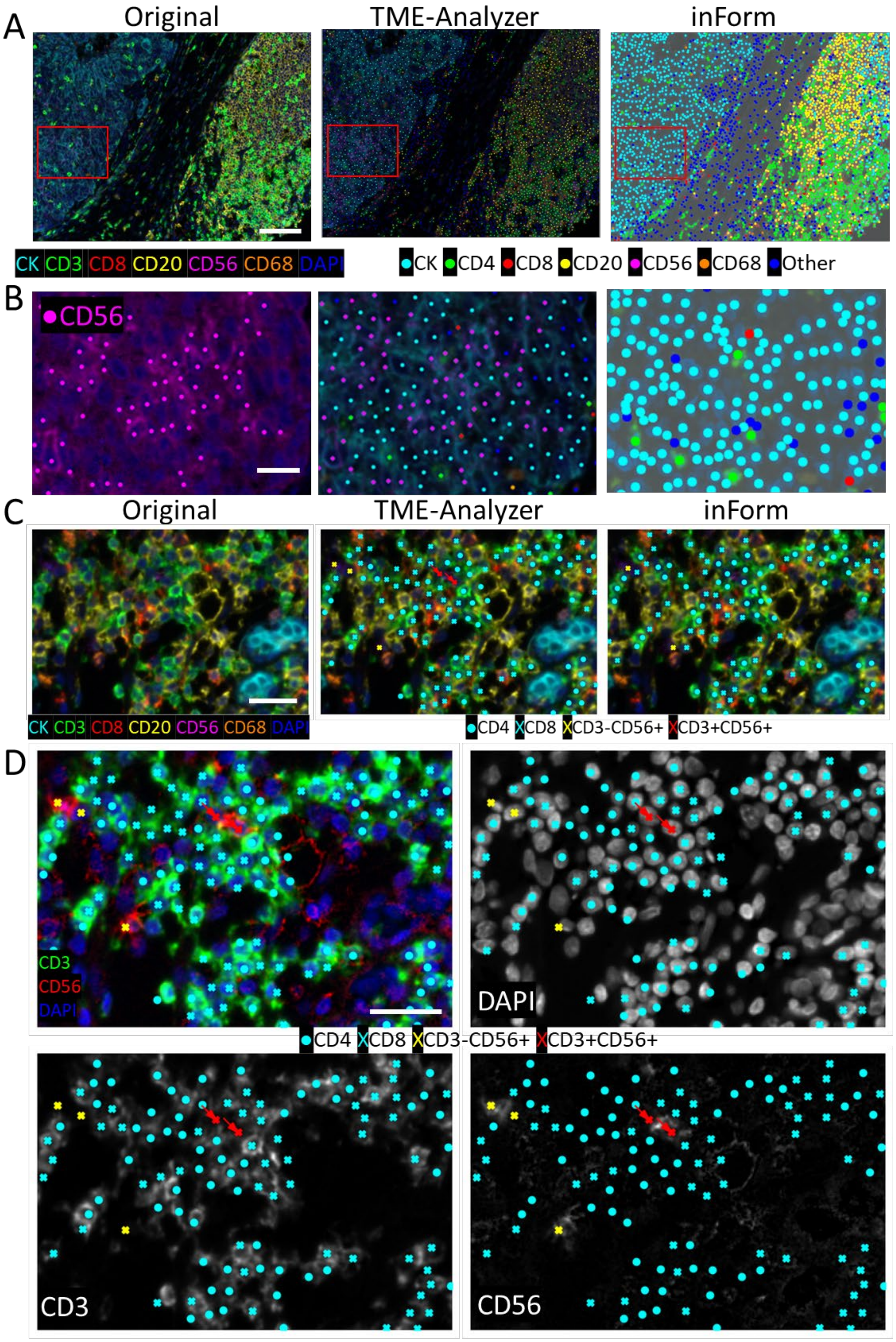

A
Original
TME-Analyzer
inForm
CK CD3 CD8 CD20 CD56 CD68 DAPI
CK CD4 CD8 CD20 CD56 CD68 Other
B
CD56
C
Original
TME-Analyzer
inForm
CK CD3 CD8 CD20 CD56 CD68 DAPI
CD4 X CD8 X CD3-CD56+ X CD3+CD56+
D
CD3
CD56
DAPI
DAPI
CD4 X CD8 X CD3-CD56+ X CD3+CD56+
CD3
CD56

Supplementary figure 4. TME-Analyzer shows improved accuracy regarding CD56 phenotyping when compared to inForm software. A,B. Representative multiplexed image (A, left) and zoom in of the red box showing only CD56 and DAPI channels together with TME-Analyzer CD56 phenotyping (B, left). Phenotyping performed with TME-Analyzer (A,B, middle) and inForm (A,B right). Note the absence of any CD56 phenotyping with inForm analysis. **C.** Representative multiplexed image (left) overlaid with its analysis using TME-Analyzer (middle) and inForm (right) highlighting cells phenotyped for CD4 (cyan circle), CD8 (cyan 'x'), CD3-CD56+ (yellow 'x') and CD3+CD56+ (red 'x'). Red arrows indicate the CD3+CD56+ cells identified with TME-Analyzer, which were identified as CD4 cells according to inForm. **(D)** RGB image showing CD3 (green) CD56 (red) and DAPI (blue) channels (top left), and monochrome images showing DAPI (top right), CD3 (bottom left) and CD56 (bottom right) channels together with the same phenotype representations as C. Scale bars are; A, 100 μm, B-D, 25 μm.

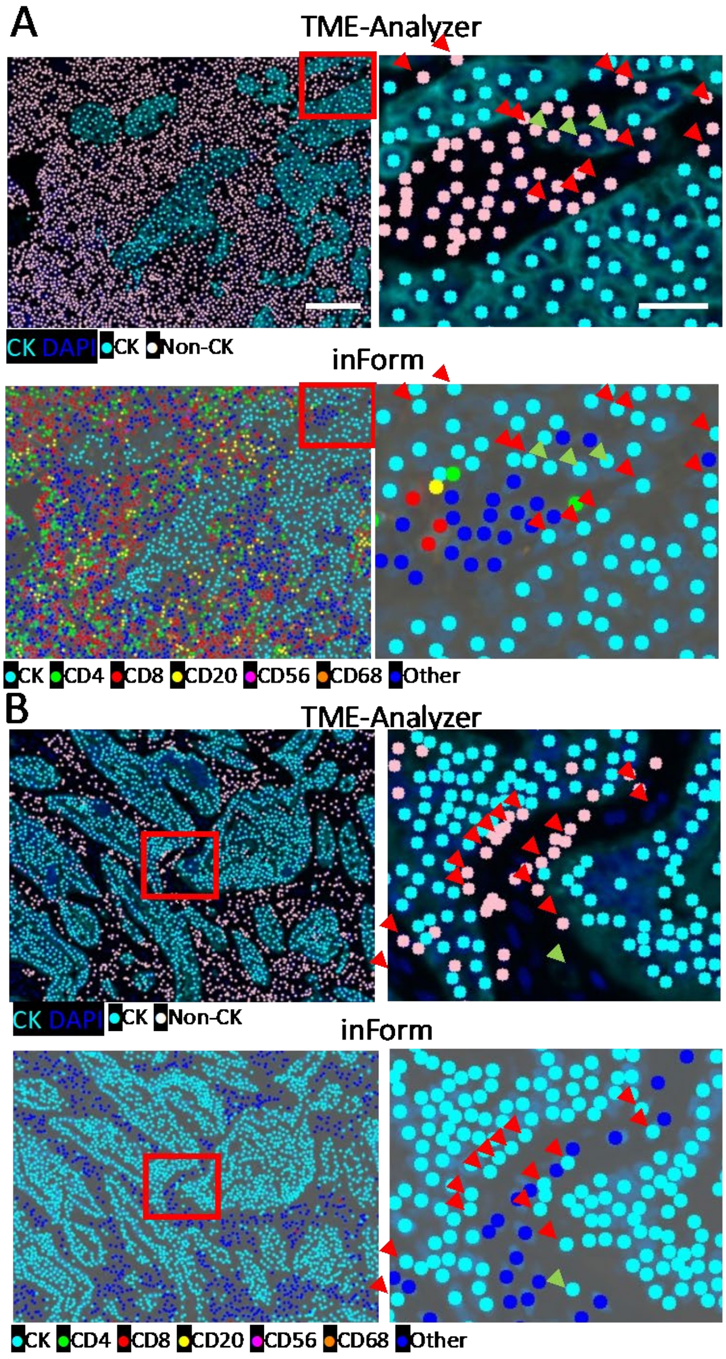

Supplementary figure 5. TME-Analyzer shows improved accuracy regarding tumor cell phenotyping when compared to inForm software. Representative images of inflamed (**A**) and non-inflamed (**B**) tumors showing cell cytokeratin positivity (cyan, left), with the zoom-in of the area indicated with the red box (right) for TME-Analyzer (top) and inForm (bottom). Arrows highlight disagreements in CK+ phenotyping between inForm and TME-Analyzer, where red and green arrows indicate cells wrongly and correctly phenotyped as CK+ by inForm but not by TME-Analyzer, respectively.

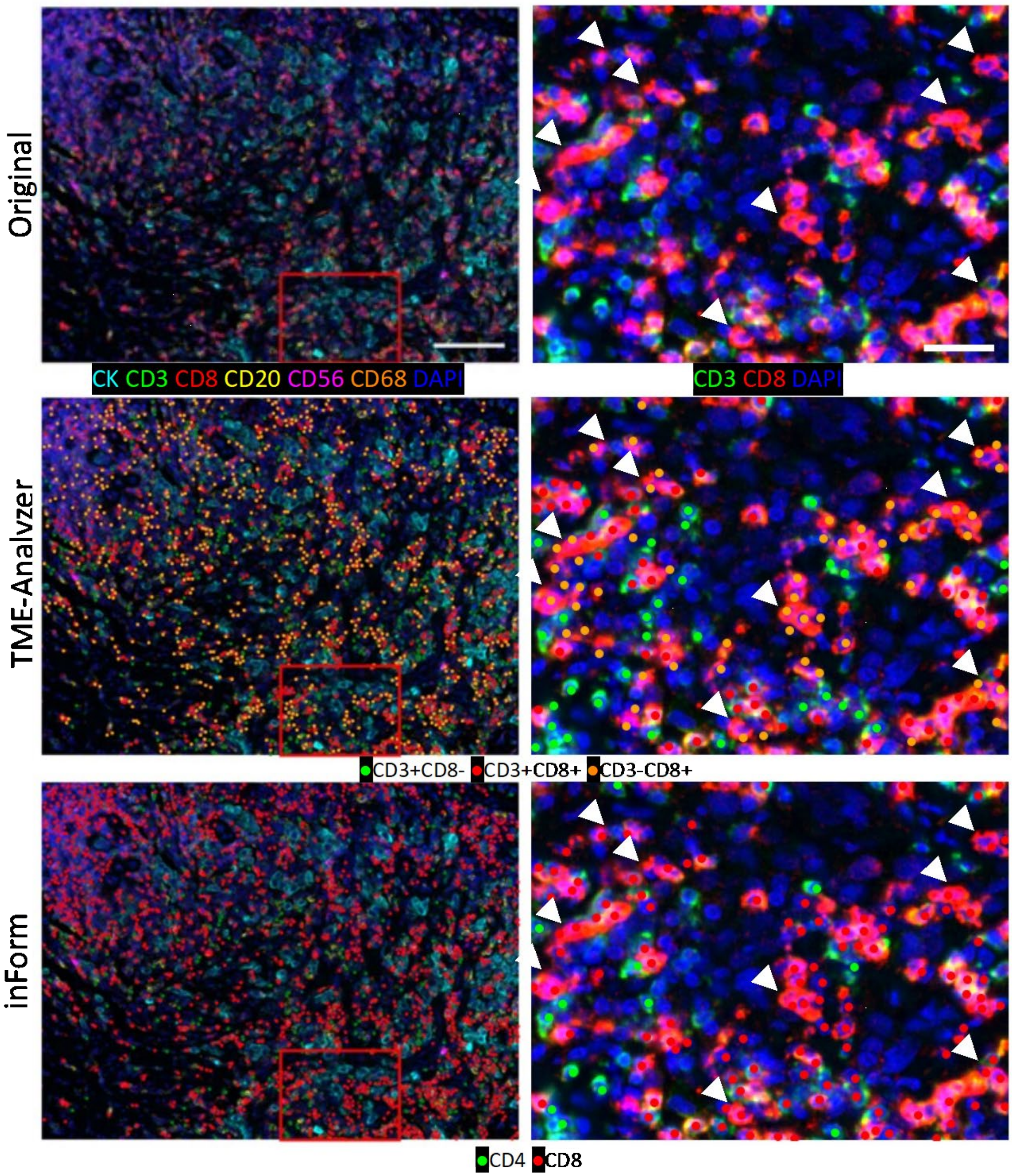

Supplementary figure 6. TME-Analyzer captures CD8 T cells according to CD3+CD8+ but not CD3-CD8+ phenotyping. Representative multiplexed image (top, left) and zoom in of the red box (top, right) showing only CD3, CD8 and DAPI channels. Phenotyping performed with TME-Analyzer (middle row) and inForm (bottom row). While TME-Analyzer could distinguish the CD3 marker positivity of CD8 T cells, in inForm, cells negative for CD3 were also

phenotyped as CD8 T cells (indicated with white arrows). Scale bars are; left: 100 µm, right 25 µm.

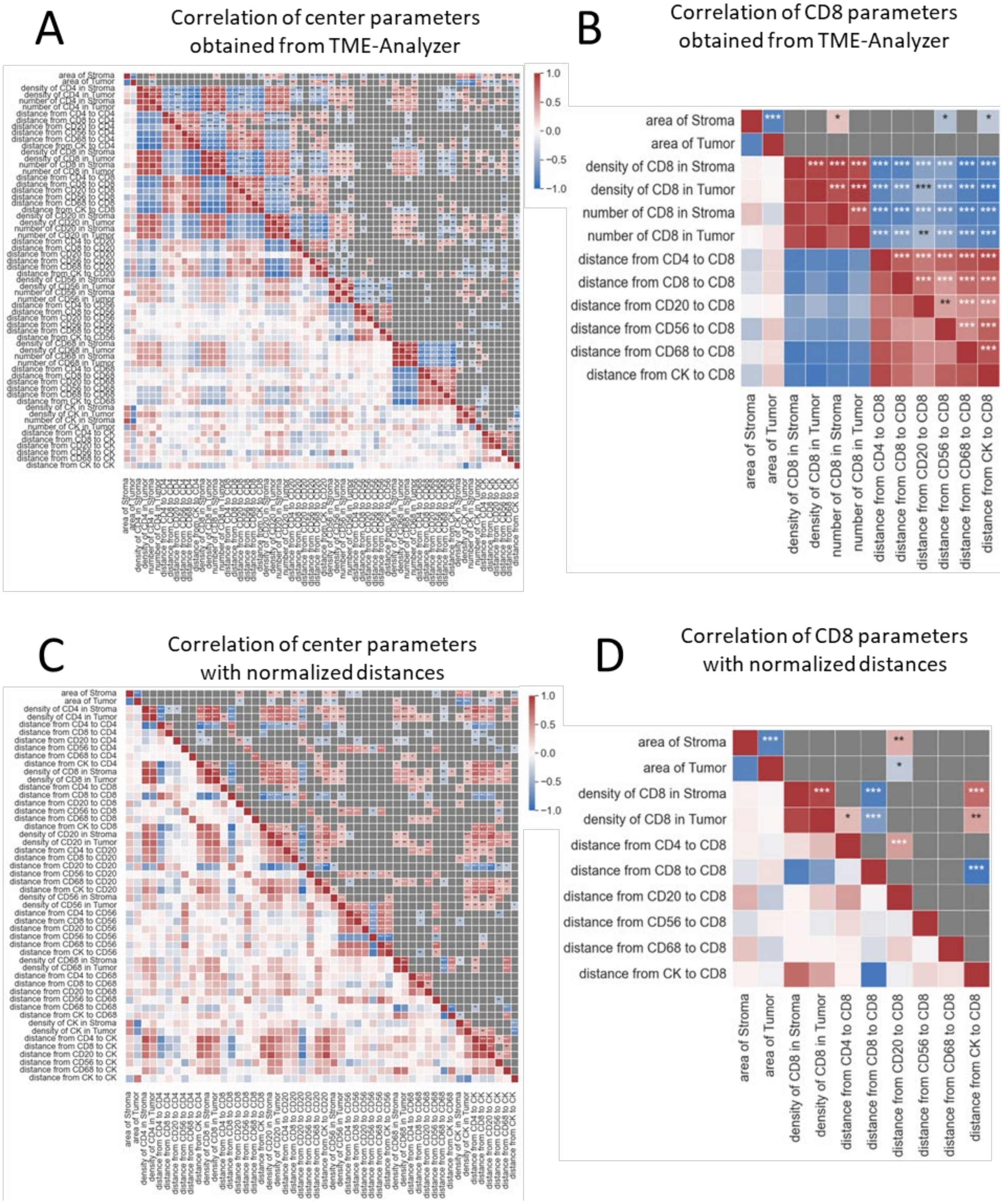

Supplementary figure 7. Normalization of inter-cellular distances according to cellular abundances. A. Correlation matrix of areas, numbers and densities of phenotyped cells in tumor and stroma compartments, and cell-cell distances at center regions (62 parameters). **B.** A sub-selection of the parameters from A (n=12) that were related to CD8 T cells. **C.** Tumor and Stroma areas, densities of phenotyped cells, and normalized cell-cell distances, which were used as input (50 parameters) to search for a contextual classifier. **D.** Sub-selection from the parameters of C (n=10) that were related to CD8 T cells.

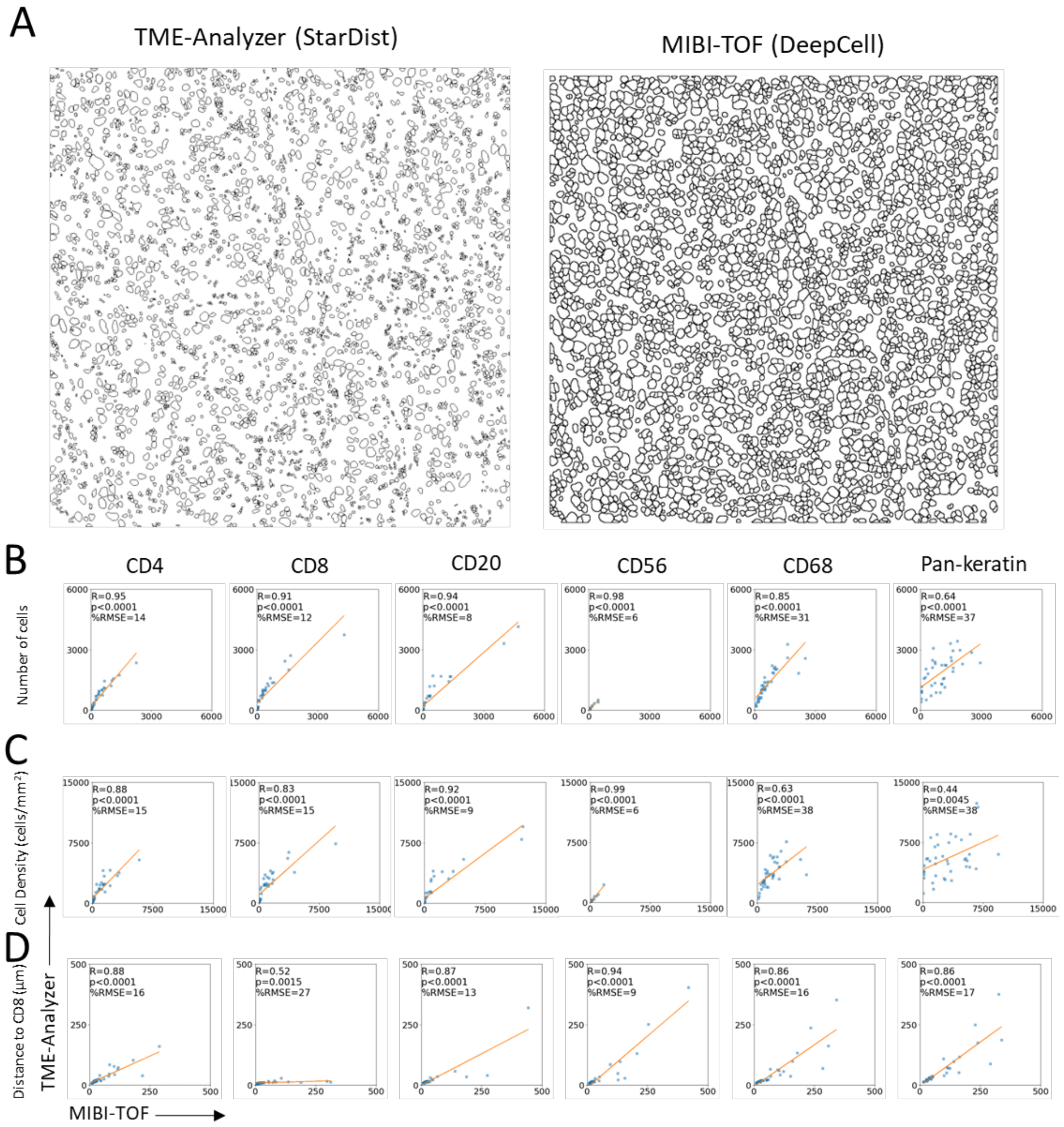

Supplementary figure 8. TME-Analyzer enables analysis of MIBI-TOF images. A. Representative nucleus segmentation images from TME-Analyzer (left) and DeepCell based MIBI-TOF segmentation (right). **B-D.** Scatter plots comparing TME-Analyzer (y-axis) vs MIBI-TOF based on DeepCell segmentation (x-axis) for numbers (B), densities (C) and distances to CD8 T cells of differently phenotyped cells (D) according to linear approximation with Pearson coefficient (R). In scatter plots, range normalized differences between TME-Analyzer and MIBI-TOF quantification is shown as percentage root mean square error (RMSE). All slopes were significantly non-zero (p<0.01) according to Wald test.
