# Supplementary figures and images for: TME-analyzer: a new interactive and dynamic image analysis tool that identified immune cell distances as predictors for survival of triple negative breast cancer patients (part 1 of 2)
Source: Npj Imaging. 2024 Jul 25;2:21. doi: 10.1038/s44303-024-00022-6 (PMC12118654; doi:10.1038/s44303-024-00022-6)

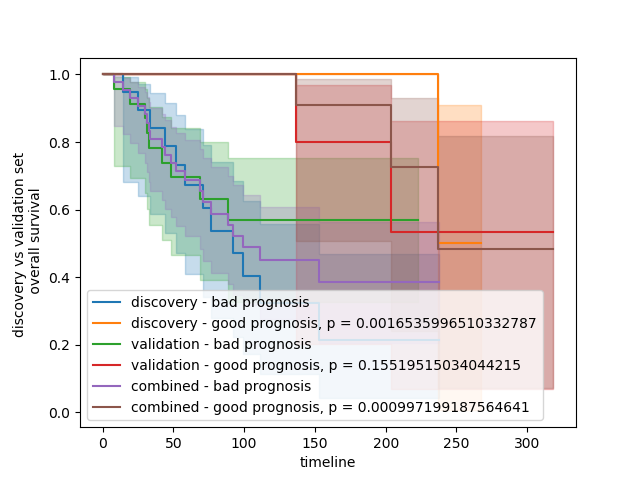

Supplement: Supplementary file 2 — interim classifiers [file 44303_2024_22_MOESM2_ESM.zip › classifier_0001.png]

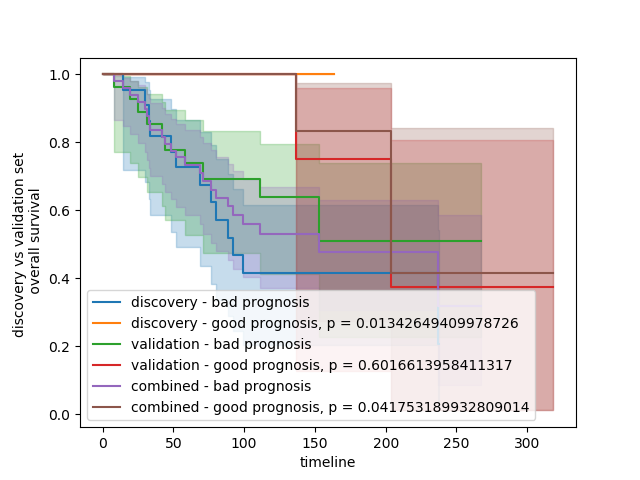

Supplement: Supplementary file 2 — interim classifiers [file 44303_2024_22_MOESM2_ESM.zip › classifier_0002.png]

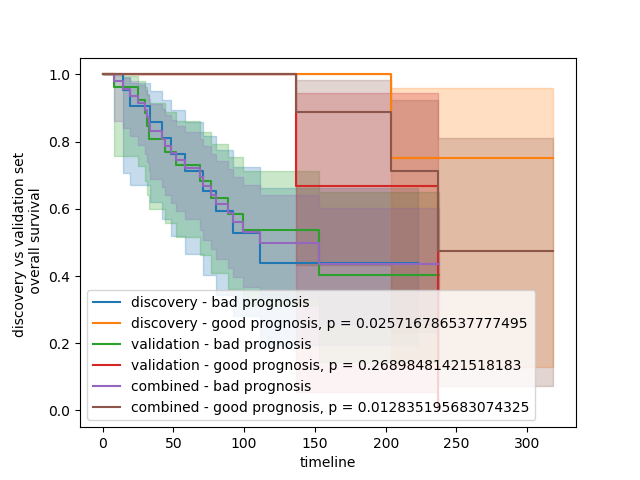

Supplement: Supplementary file 2 — interim classifiers [file 44303_2024_22_MOESM2_ESM.zip › classifier_0003.png]

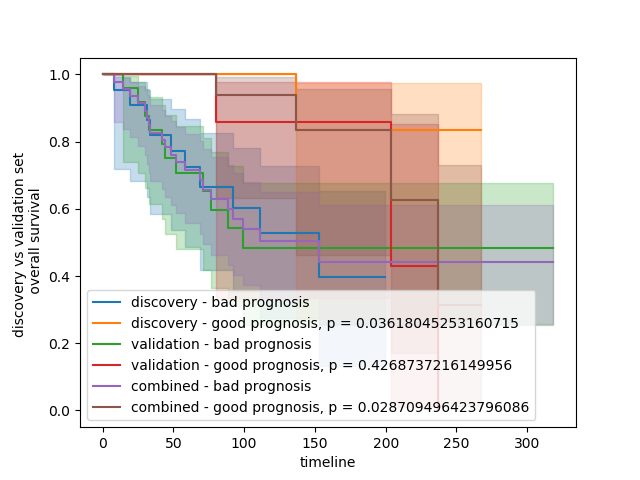

Supplement: Supplementary file 2 — interim classifiers [file 44303_2024_22_MOESM2_ESM.zip › classifier_0004.png]

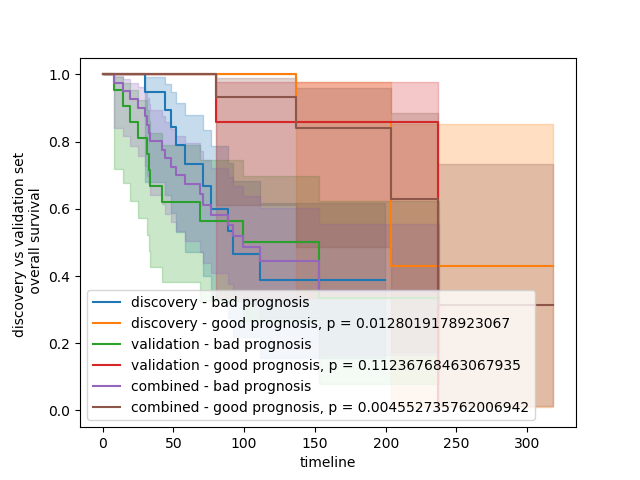

Supplement: Supplementary file 2 — interim classifiers [file 44303_2024_22_MOESM2_ESM.zip › classifier_0005.png]

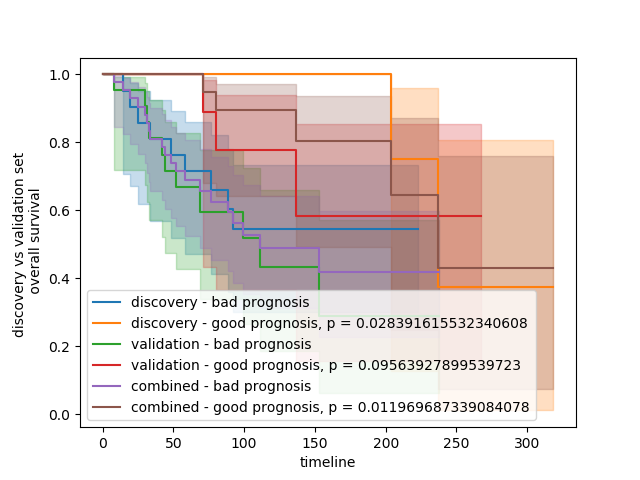

Supplement: Supplementary file 2 — interim classifiers [file 44303_2024_22_MOESM2_ESM.zip › classifier_0006.png]

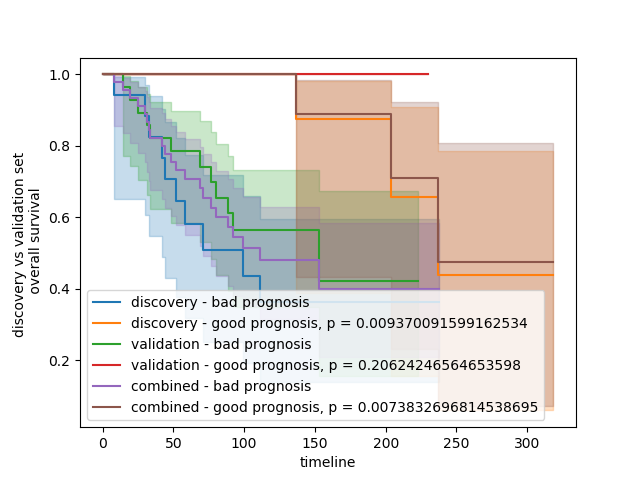

Supplement: Supplementary file 2 — interim classifiers [file 44303_2024_22_MOESM2_ESM.zip › classifier_0007.png]

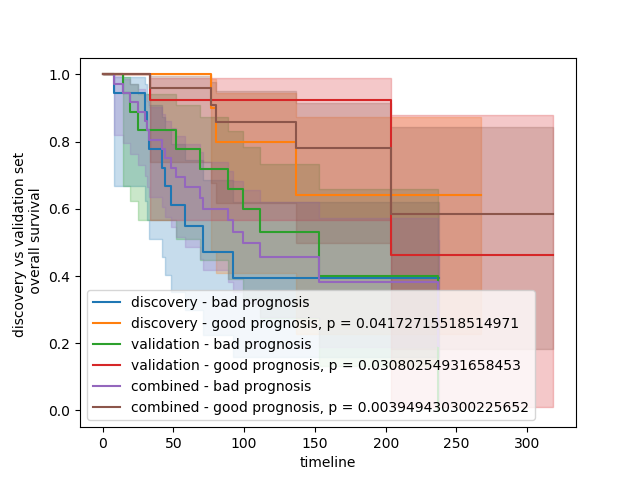

Supplement: Supplementary file 2 — interim classifiers [file 44303_2024_22_MOESM2_ESM.zip › classifier_0008.png]

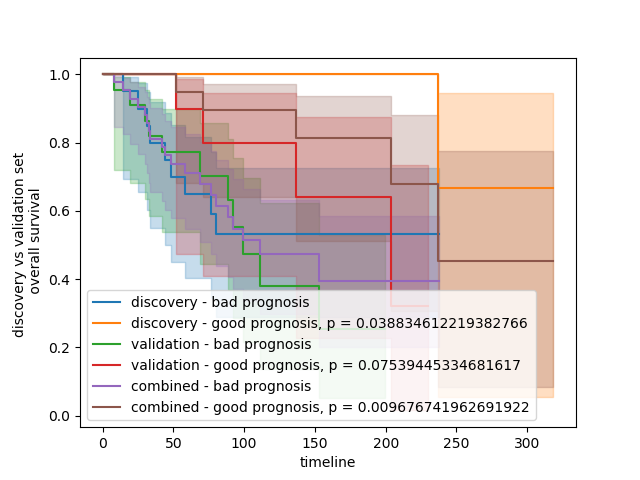

Supplement: Supplementary file 2 — interim classifiers [file 44303_2024_22_MOESM2_ESM.zip › classifier_0009.png]

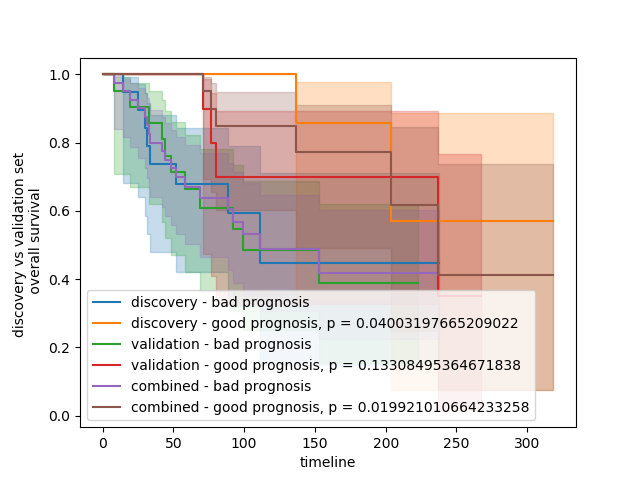

Supplement: Supplementary file 2 — interim classifiers [file 44303_2024_22_MOESM2_ESM.zip › classifier_0010.png]

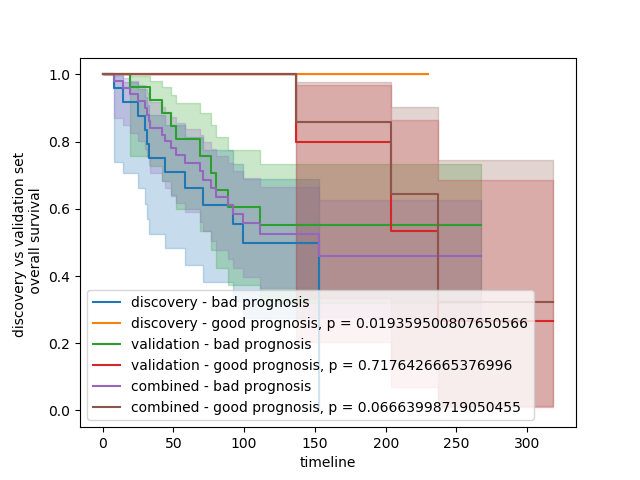

Supplement: Supplementary file 2 — interim classifiers [file 44303_2024_22_MOESM2_ESM.zip › classifier_0011.png]

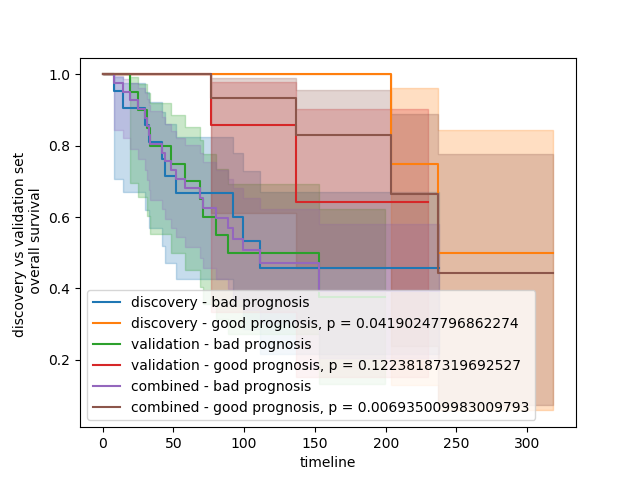

Supplement: Supplementary file 2 — interim classifiers [file 44303_2024_22_MOESM2_ESM.zip › classifier_0012.png]

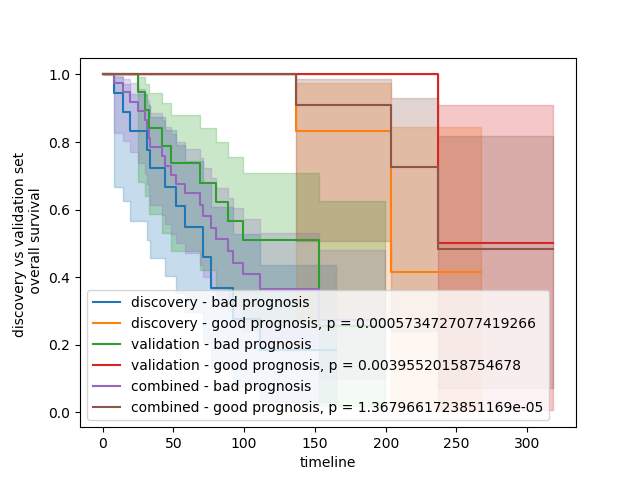

Supplement: Supplementary file 2 — interim classifiers [file 44303_2024_22_MOESM2_ESM.zip › classifier_0013.png]

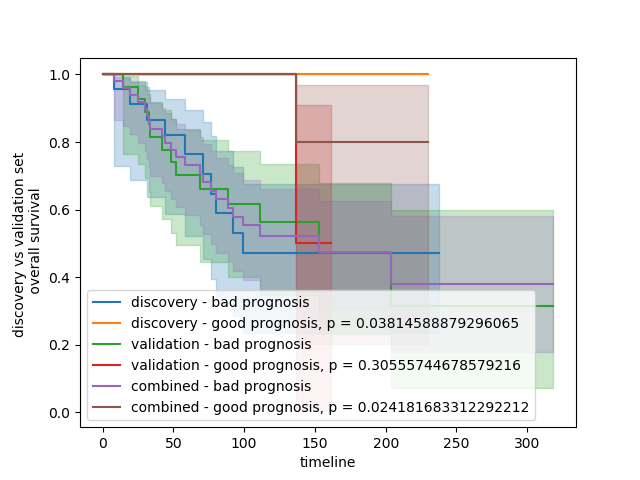

Supplement: Supplementary file 2 — interim classifiers [file 44303_2024_22_MOESM2_ESM.zip › classifier_0014.png]

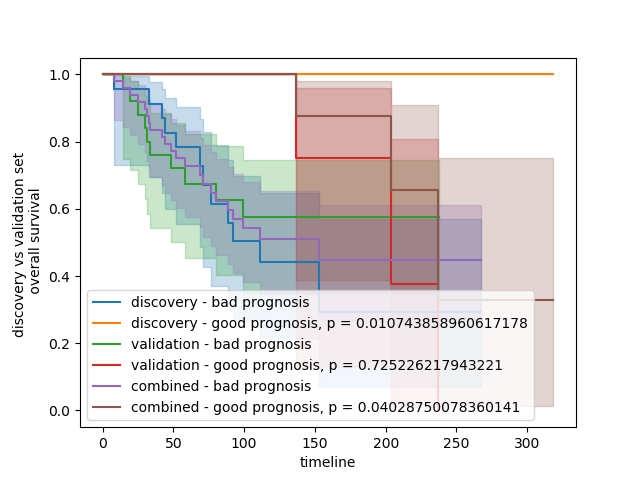

Supplement: Supplementary file 2 — interim classifiers [file 44303_2024_22_MOESM2_ESM.zip › classifier_0015.png]

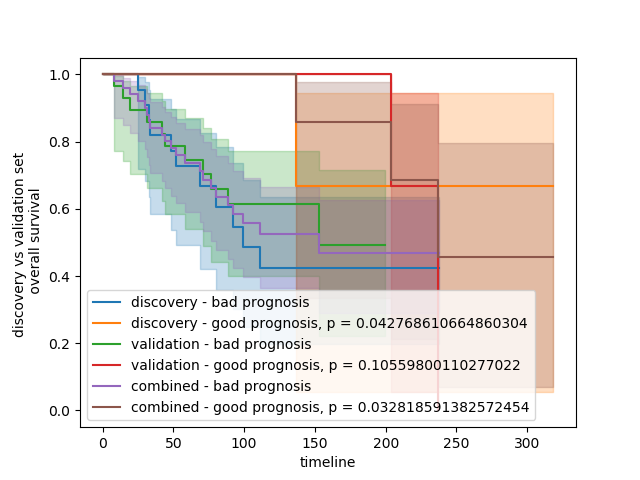

Supplement: Supplementary file 2 — interim classifiers [file 44303_2024_22_MOESM2_ESM.zip › classifier_0016.png]

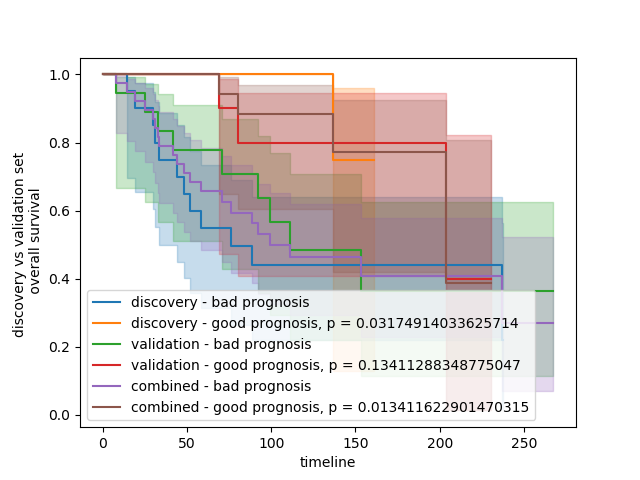

Supplement: Supplementary file 2 — interim classifiers [file 44303_2024_22_MOESM2_ESM.zip › classifier_0017.png]

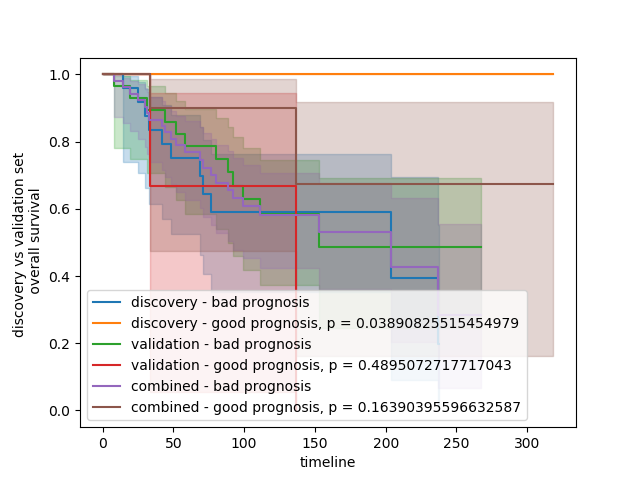

Supplement: Supplementary file 2 — interim classifiers [file 44303_2024_22_MOESM2_ESM.zip › classifier_0018.png]

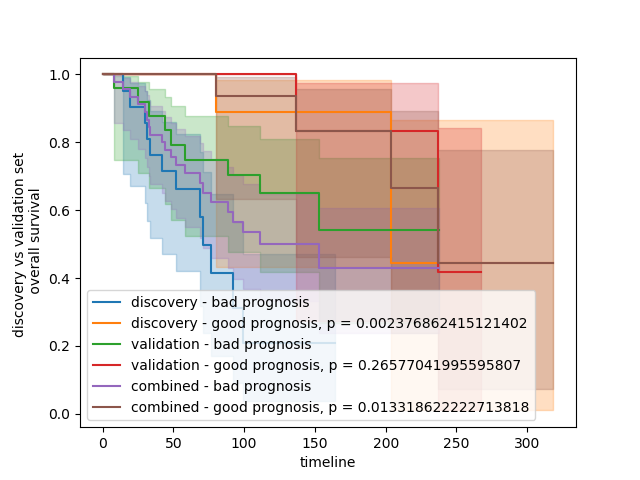

Supplement: Supplementary file 2 — interim classifiers [file 44303_2024_22_MOESM2_ESM.zip › classifier_0019.png]

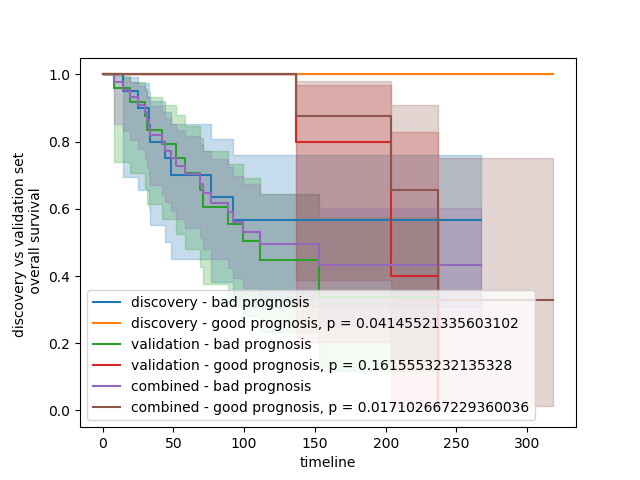

Supplement: Supplementary file 2 — interim classifiers [file 44303_2024_22_MOESM2_ESM.zip › classifier_0020.png]

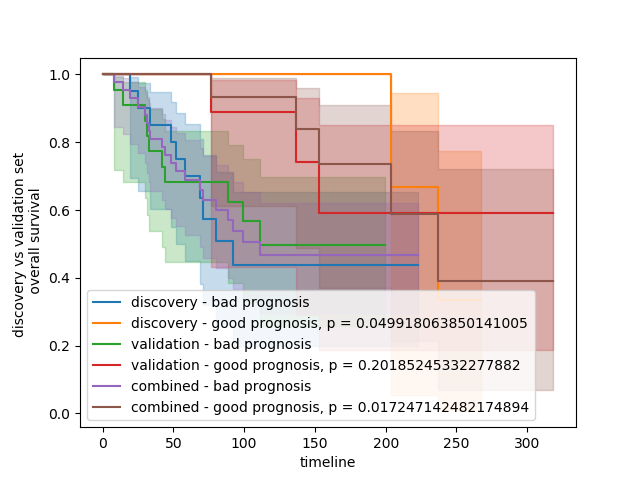

Supplement: Supplementary file 2 — interim classifiers [file 44303_2024_22_MOESM2_ESM.zip › classifier_0021.png]

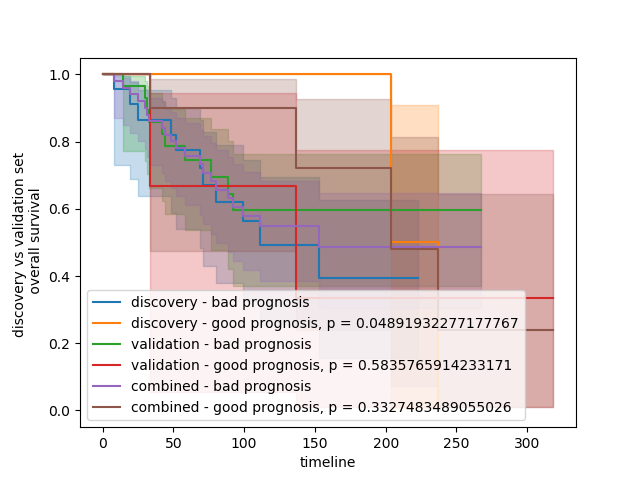

Supplement: Supplementary file 2 — interim classifiers [file 44303_2024_22_MOESM2_ESM.zip › classifier_0022.png]

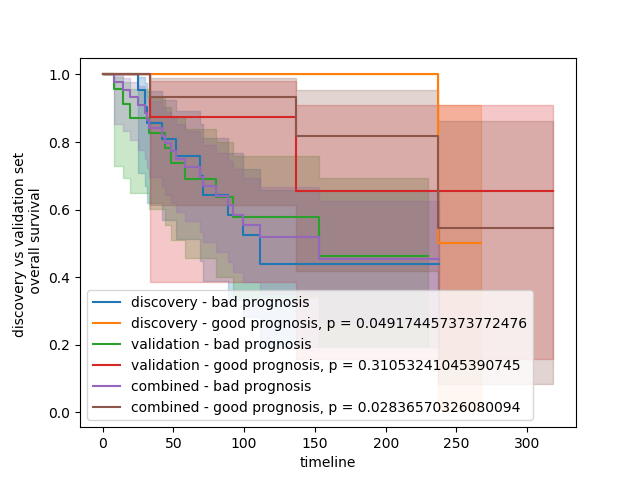

Supplement: Supplementary file 2 — interim classifiers [file 44303_2024_22_MOESM2_ESM.zip › classifier_0023.png]

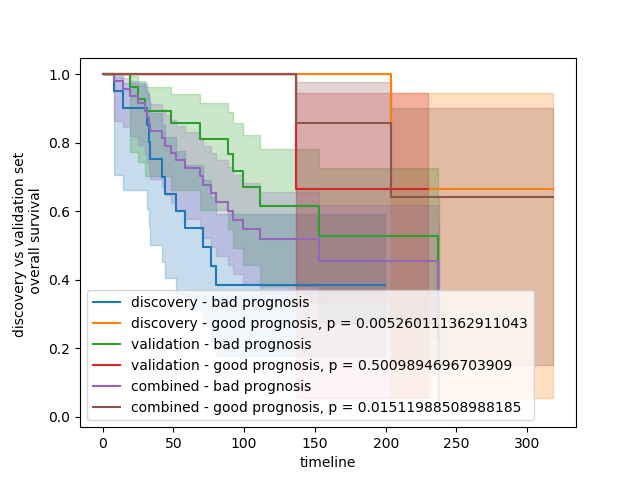

Supplement: Supplementary file 2 — interim classifiers [file 44303_2024_22_MOESM2_ESM.zip › classifier_0024.png]

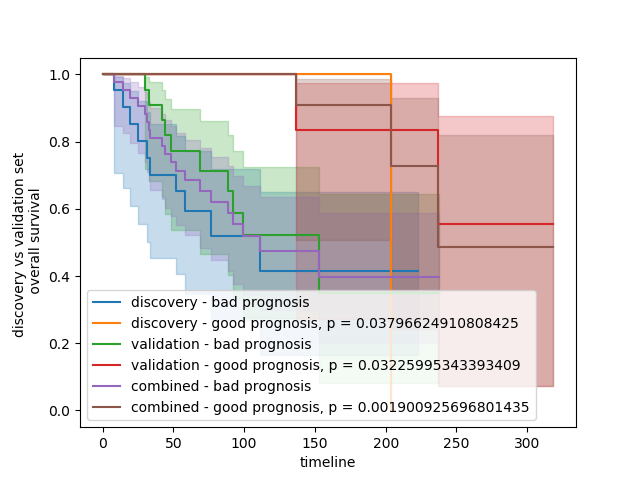

Supplement: Supplementary file 2 — interim classifiers [file 44303_2024_22_MOESM2_ESM.zip › classifier_0025.png]

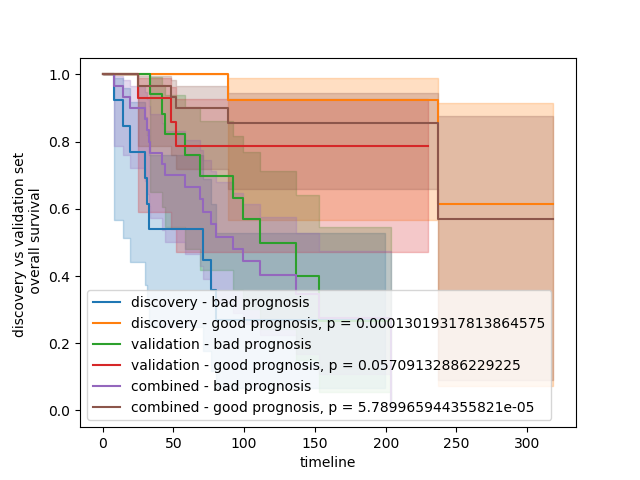

Supplement: Supplementary file 2 — interim classifiers [file 44303_2024_22_MOESM2_ESM.zip › classifier_0026.png]

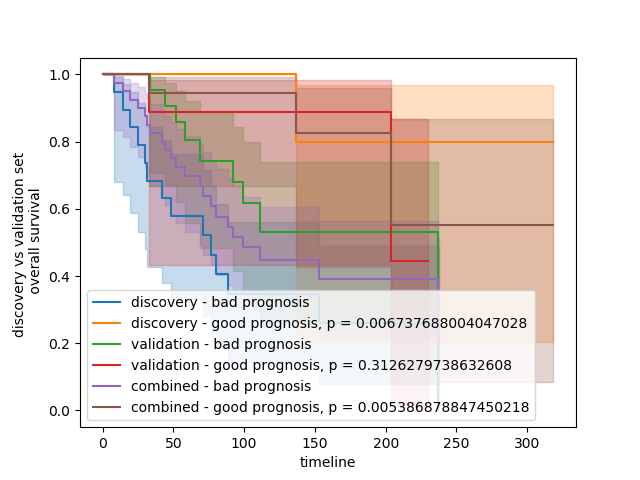

Supplement: Supplementary file 2 — interim classifiers [file 44303_2024_22_MOESM2_ESM.zip › classifier_0027.png]

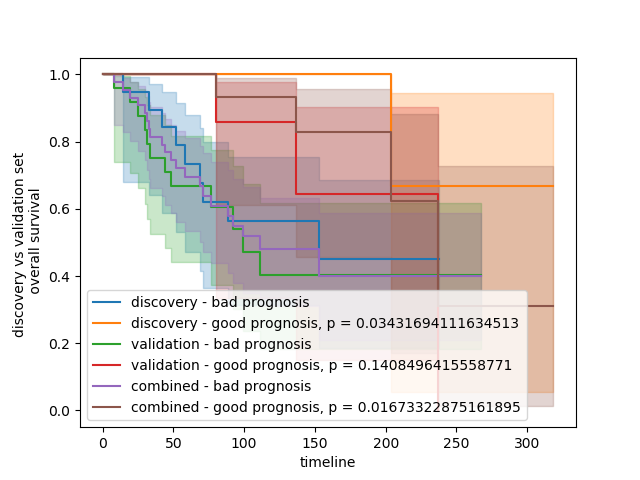

Supplement: Supplementary file 2 — interim classifiers [file 44303_2024_22_MOESM2_ESM.zip › classifier_0028.png]

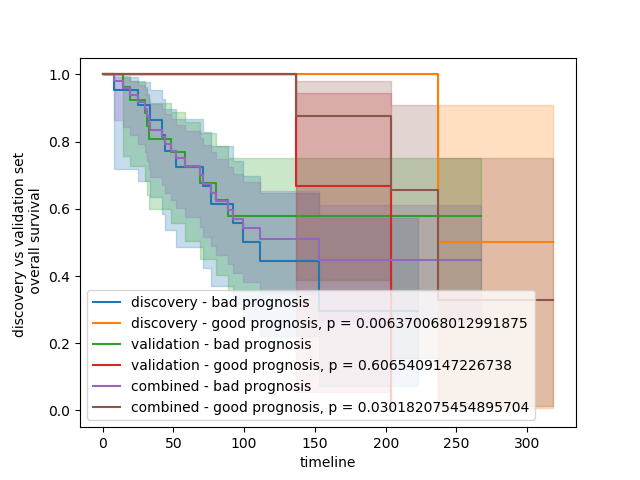

Supplement: Supplementary file 2 — interim classifiers [file 44303_2024_22_MOESM2_ESM.zip › classifier_0029.png]

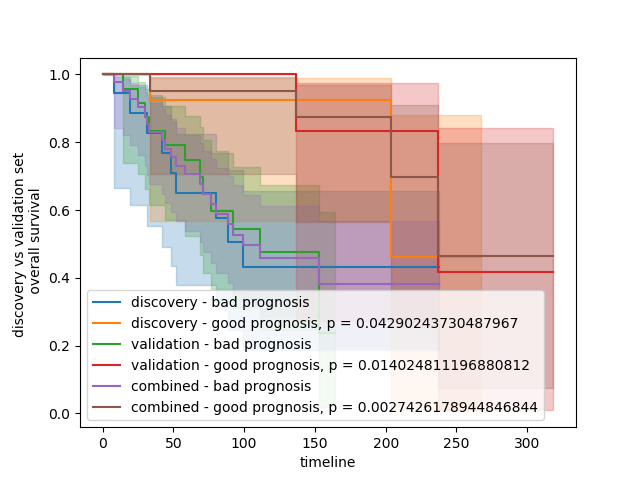

Supplement: Supplementary file 2 — interim classifiers [file 44303_2024_22_MOESM2_ESM.zip › classifier_0030.png]

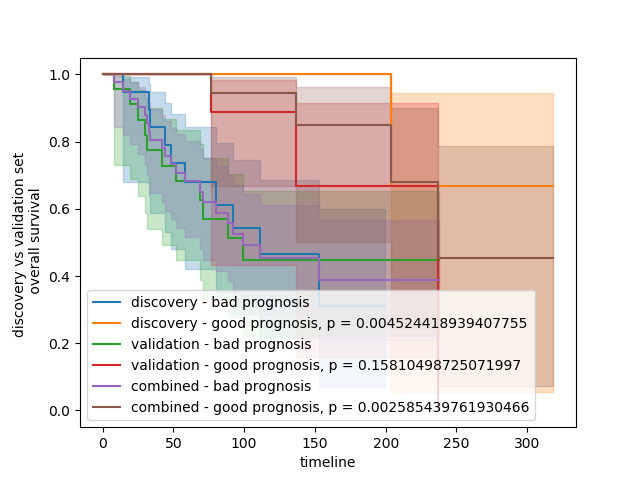

Supplement: Supplementary file 2 — interim classifiers [file 44303_2024_22_MOESM2_ESM.zip › classifier_0031.png]

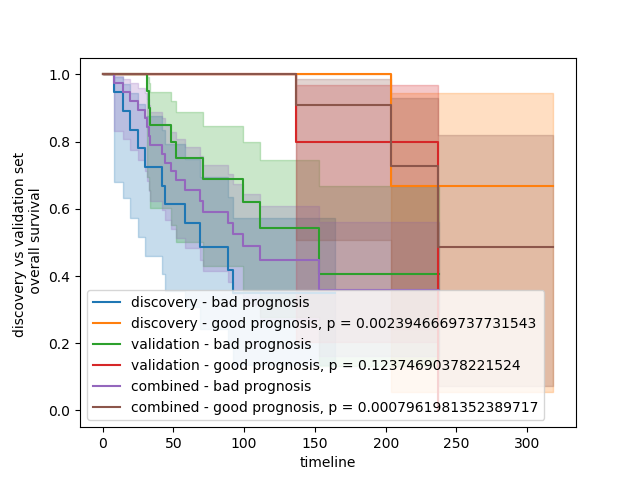

Supplement: Supplementary file 2 — interim classifiers [file 44303_2024_22_MOESM2_ESM.zip › classifier_0032.png]

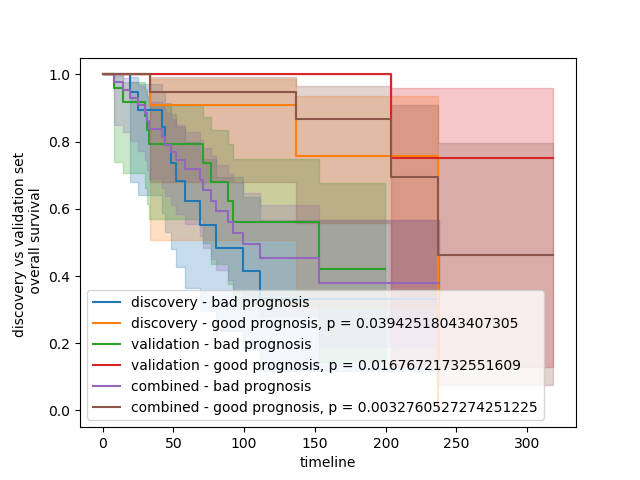

Supplement: Supplementary file 2 — interim classifiers [file 44303_2024_22_MOESM2_ESM.zip › classifier_0033.png]

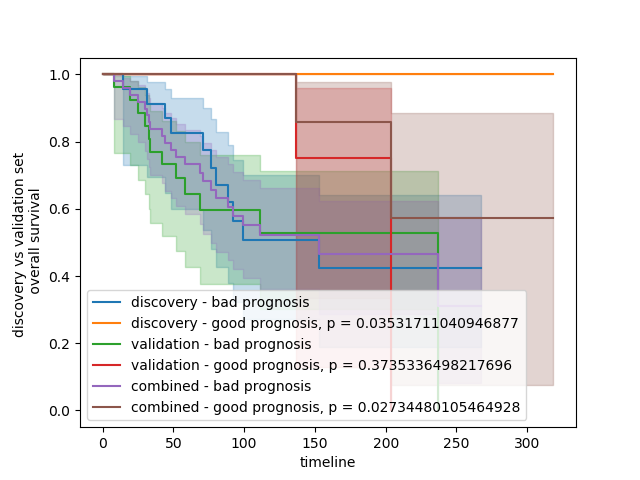

Supplement: Supplementary file 2 — interim classifiers [file 44303_2024_22_MOESM2_ESM.zip › classifier_0034.png]

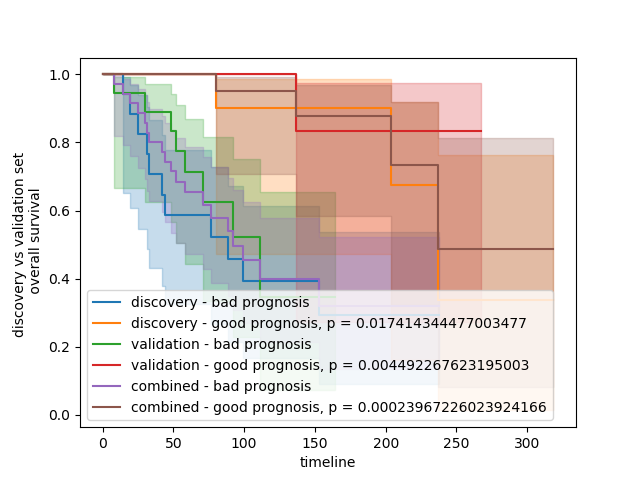

Supplement: Supplementary file 2 — interim classifiers [file 44303_2024_22_MOESM2_ESM.zip › classifier_0035.png]

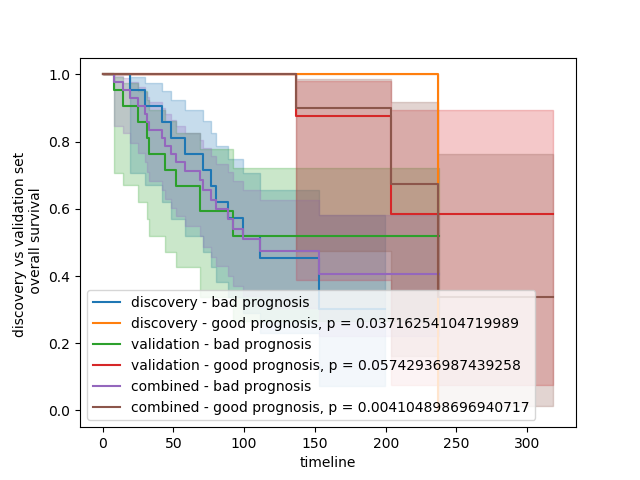

Supplement: Supplementary file 2 — interim classifiers [file 44303_2024_22_MOESM2_ESM.zip › classifier_0036.png]

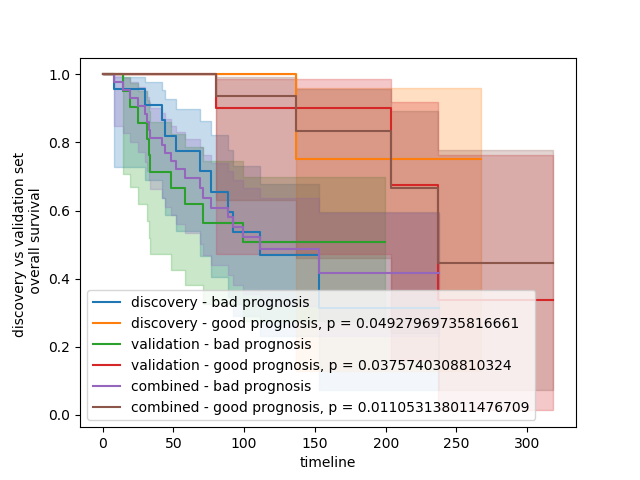

Supplement: Supplementary file 2 — interim classifiers [file 44303_2024_22_MOESM2_ESM.zip › classifier_0037.png]

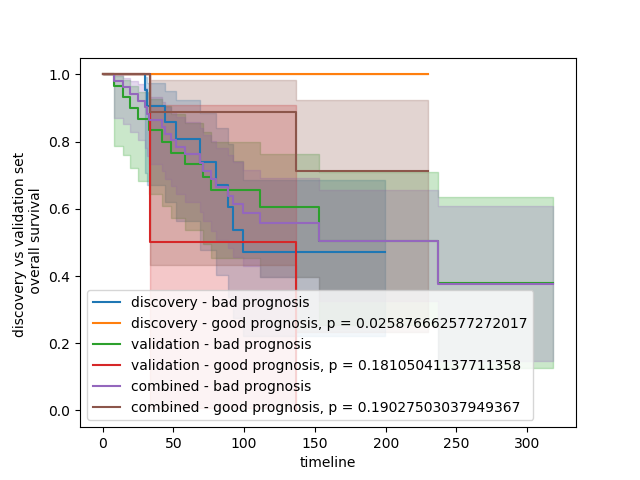

Supplement: Supplementary file 2 — interim classifiers [file 44303_2024_22_MOESM2_ESM.zip › classifier_0038.png]

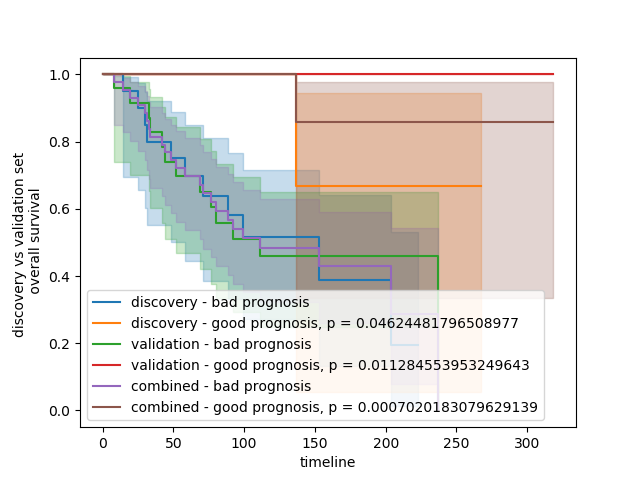

Supplement: Supplementary file 2 — interim classifiers [file 44303_2024_22_MOESM2_ESM.zip › classifier_0039.png]

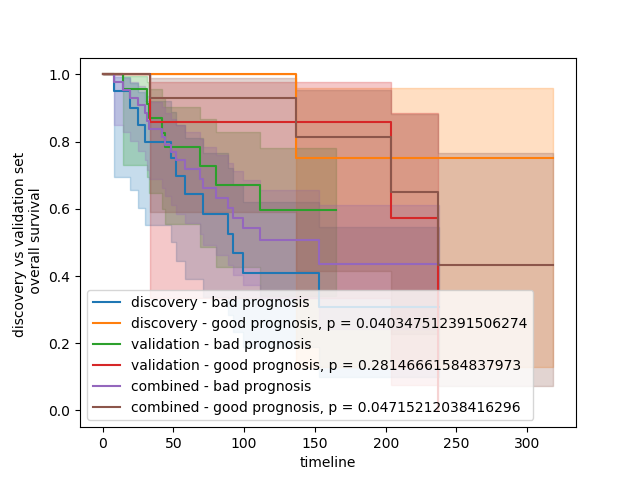

Supplement: Supplementary file 2 — interim classifiers [file 44303_2024_22_MOESM2_ESM.zip › classifier_0040.png]

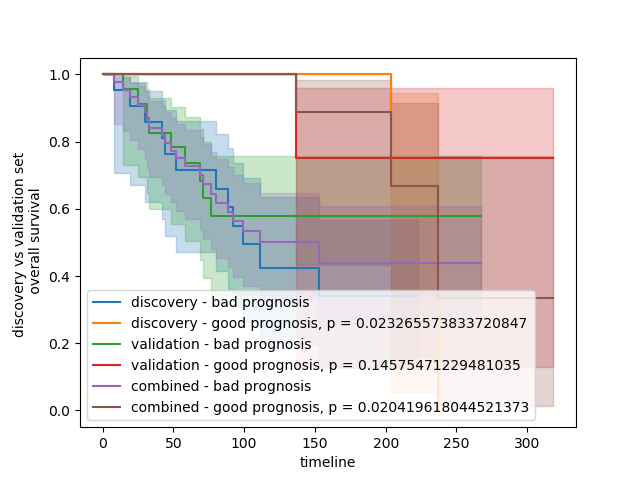

Supplement: Supplementary file 2 — interim classifiers [file 44303_2024_22_MOESM2_ESM.zip › classifier_0041.png]

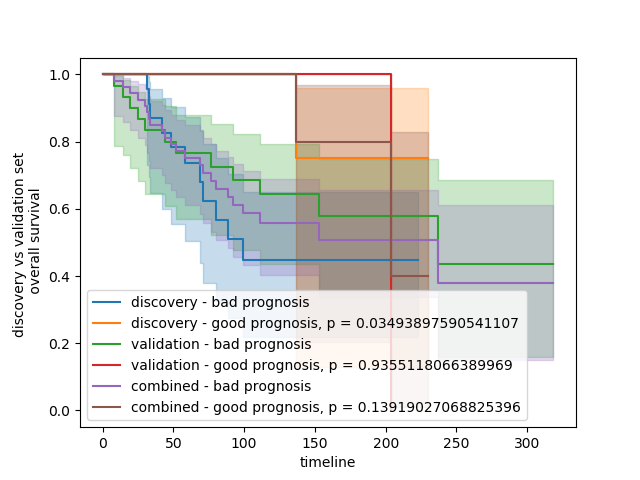

Supplement: Supplementary file 2 — interim classifiers [file 44303_2024_22_MOESM2_ESM.zip › classifier_0042.png]

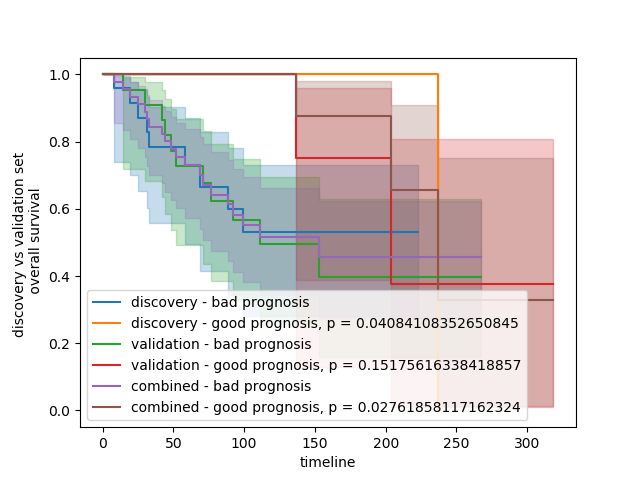

Supplement: Supplementary file 2 — interim classifiers [file 44303_2024_22_MOESM2_ESM.zip › classifier_0043.png]

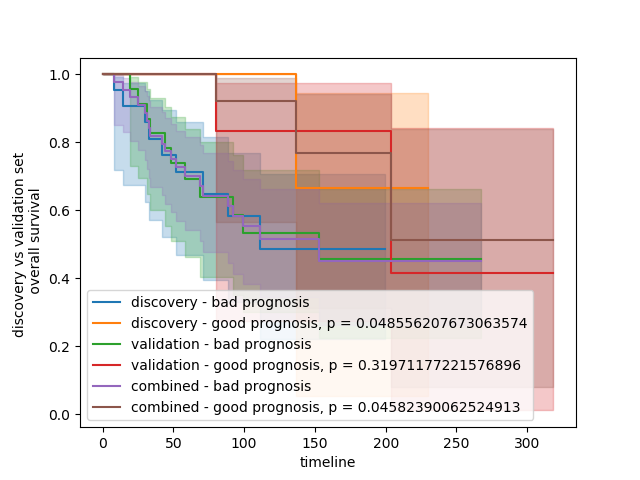

Supplement: Supplementary file 2 — interim classifiers [file 44303_2024_22_MOESM2_ESM.zip › classifier_0044.png]

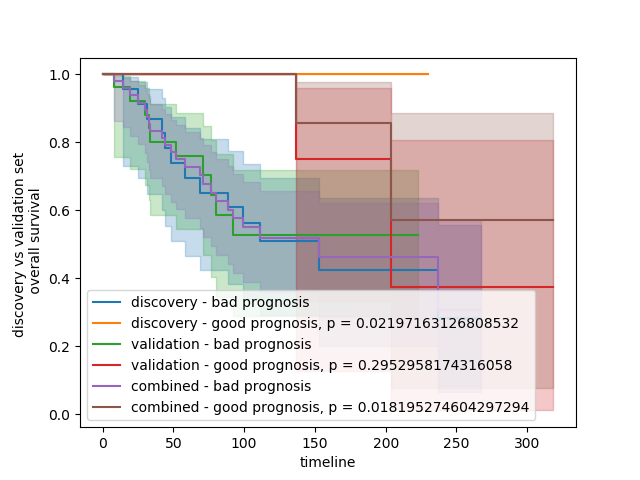

Supplement: Supplementary file 2 — interim classifiers [file 44303_2024_22_MOESM2_ESM.zip › classifier_0045.png]

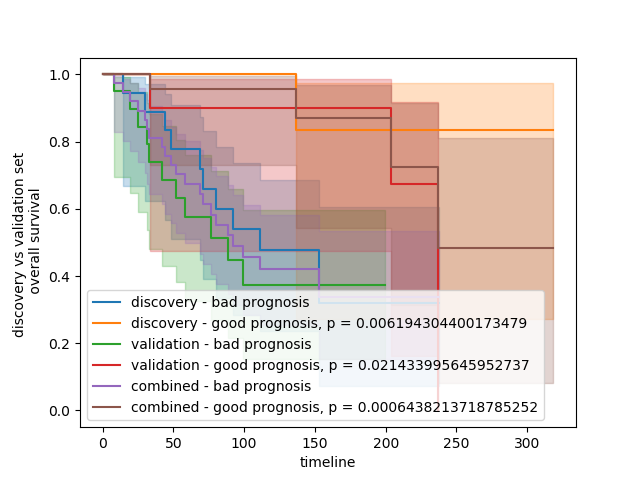

Supplement: Supplementary file 2 — interim classifiers [file 44303_2024_22_MOESM2_ESM.zip › classifier_0046.png]

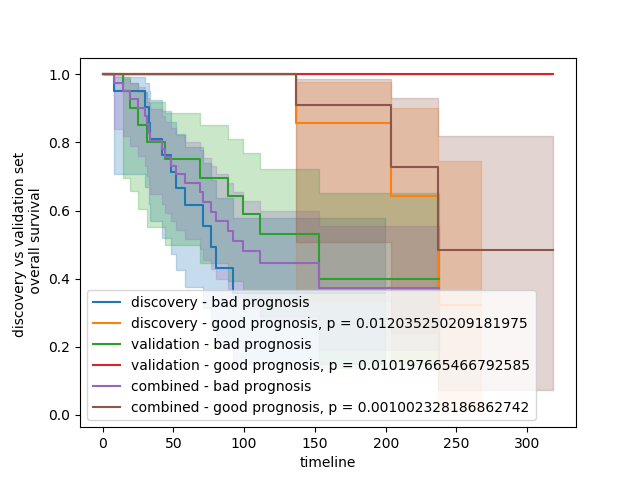

Supplement: Supplementary file 2 — interim classifiers [file 44303_2024_22_MOESM2_ESM.zip › classifier_0047.png]

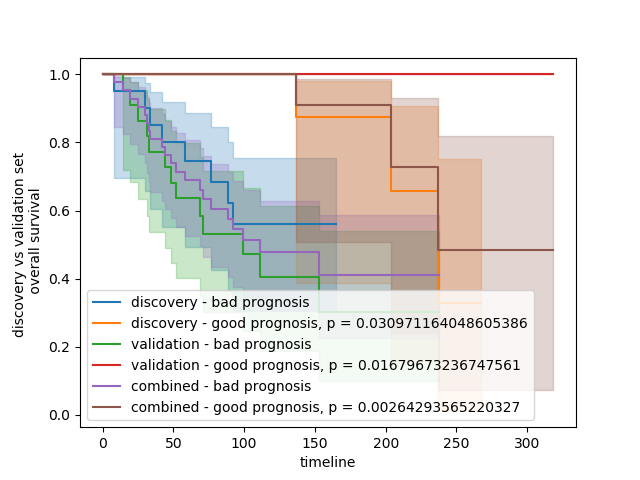

Supplement: Supplementary file 2 — interim classifiers [file 44303_2024_22_MOESM2_ESM.zip › classifier_0048.png]

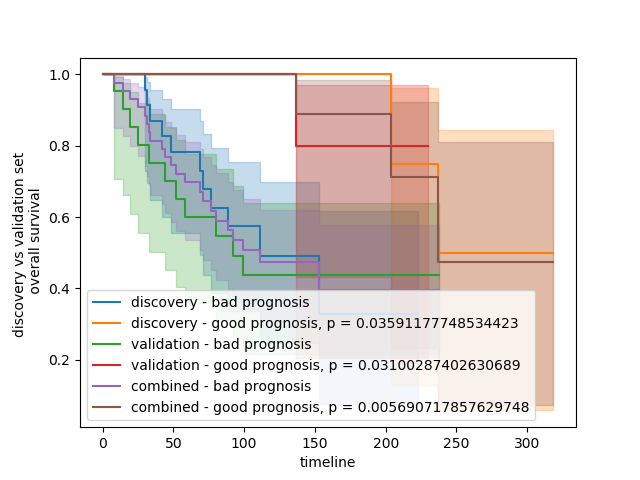

Supplement: Supplementary file 2 — interim classifiers [file 44303_2024_22_MOESM2_ESM.zip › classifier_0049.png]

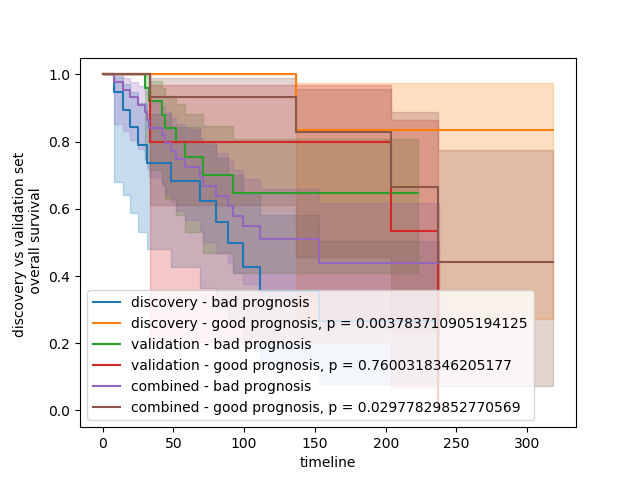

Supplement: Supplementary file 2 — interim classifiers [file 44303_2024_22_MOESM2_ESM.zip › classifier_0050.png]

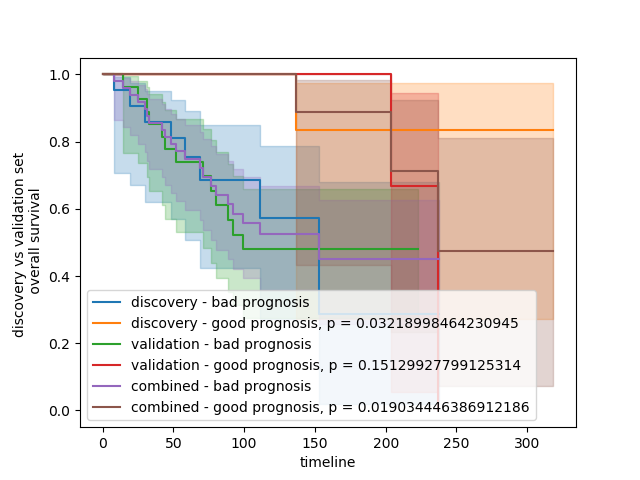

Supplement: Supplementary file 2 — interim classifiers [file 44303_2024_22_MOESM2_ESM.zip › classifier_0051.png]

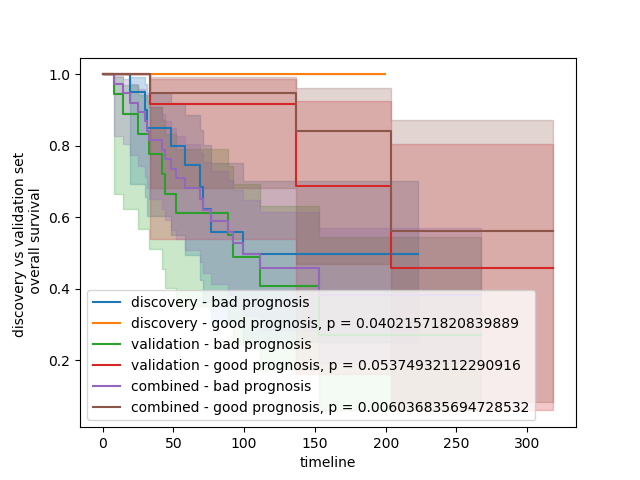

Supplement: Supplementary file 2 — interim classifiers [file 44303_2024_22_MOESM2_ESM.zip › classifier_0052.png]

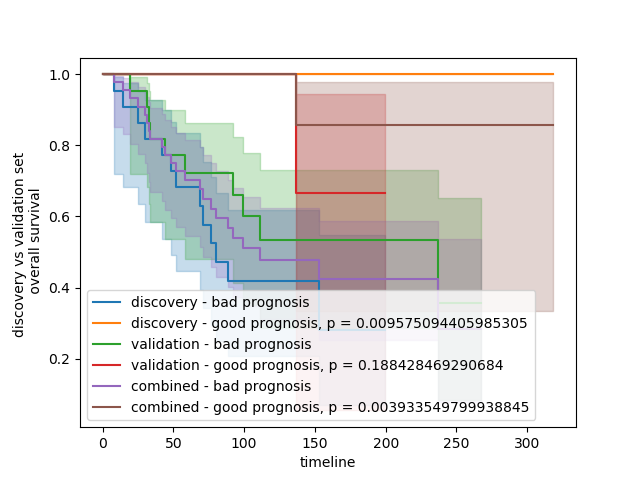

Supplement: Supplementary file 2 — interim classifiers [file 44303_2024_22_MOESM2_ESM.zip › classifier_0053.png]

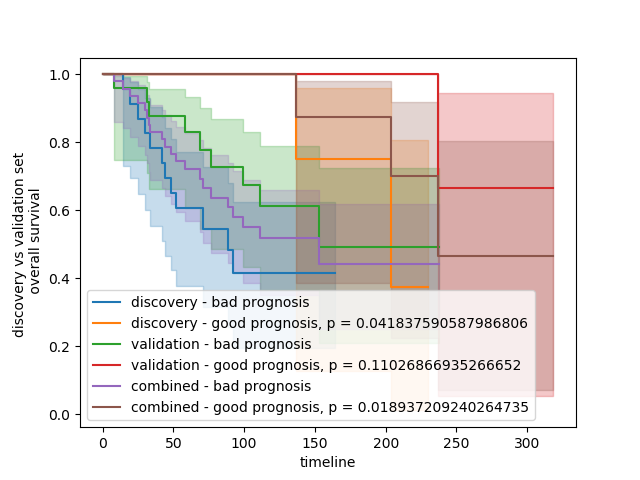

Supplement: Supplementary file 2 — interim classifiers [file 44303_2024_22_MOESM2_ESM.zip › classifier_0054.png]

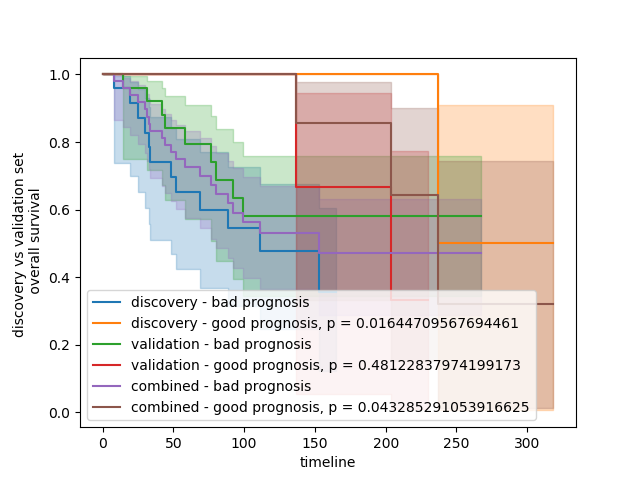

Supplement: Supplementary file 2 — interim classifiers [file 44303_2024_22_MOESM2_ESM.zip › classifier_0055.png]

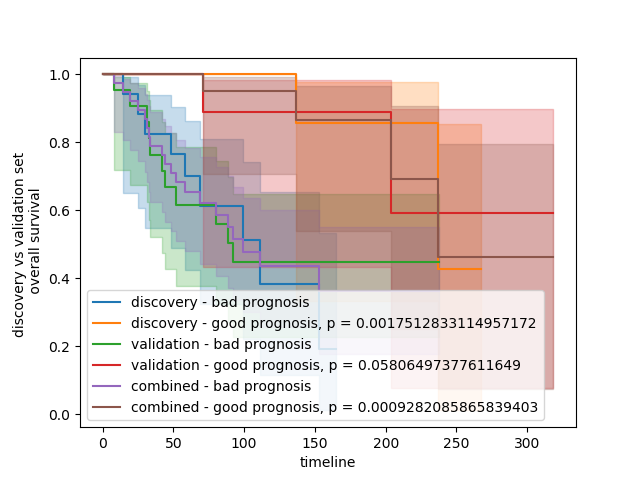

Supplement: Supplementary file 2 — interim classifiers [file 44303_2024_22_MOESM2_ESM.zip › classifier_0056.png]

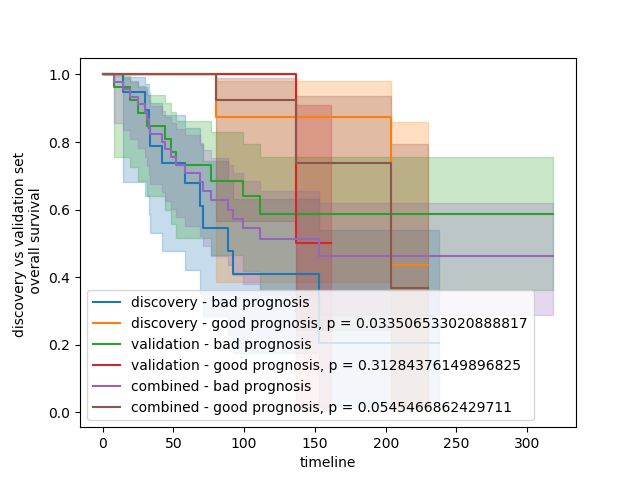

Supplement: Supplementary file 2 — interim classifiers [file 44303_2024_22_MOESM2_ESM.zip › classifier_0057.png]

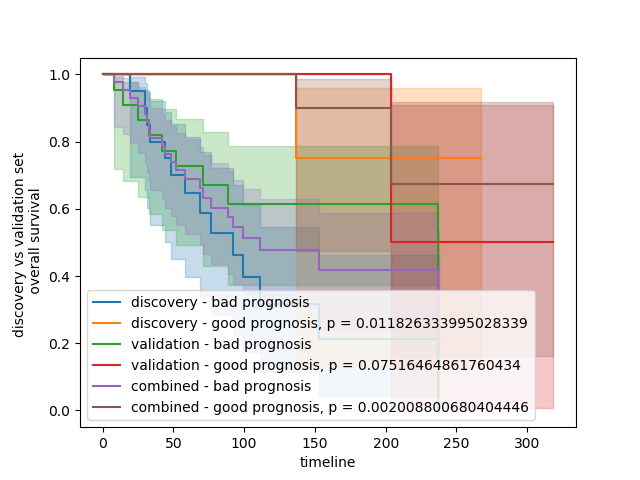

Supplement: Supplementary file 2 — interim classifiers [file 44303_2024_22_MOESM2_ESM.zip › classifier_0058.png]

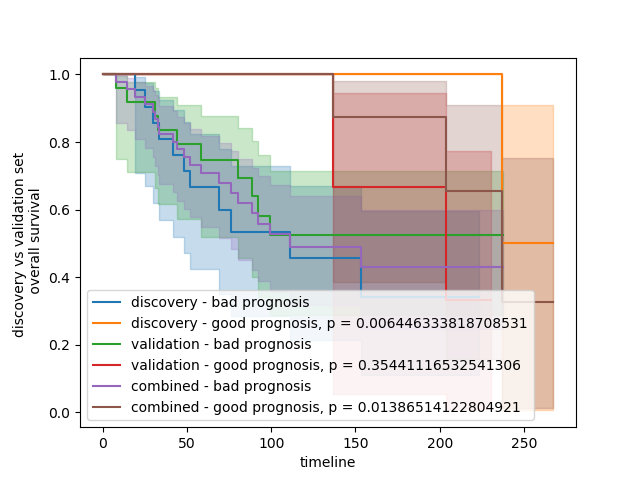

Supplement: Supplementary file 2 — interim classifiers [file 44303_2024_22_MOESM2_ESM.zip › classifier_0059.png]

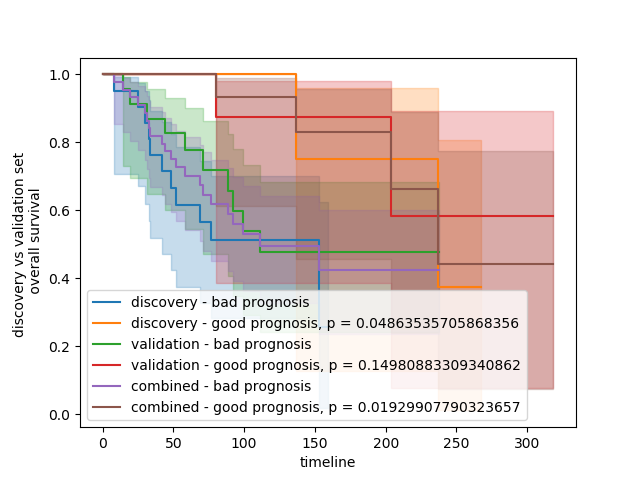

Supplement: Supplementary file 2 — interim classifiers [file 44303_2024_22_MOESM2_ESM.zip › classifier_0060.png]

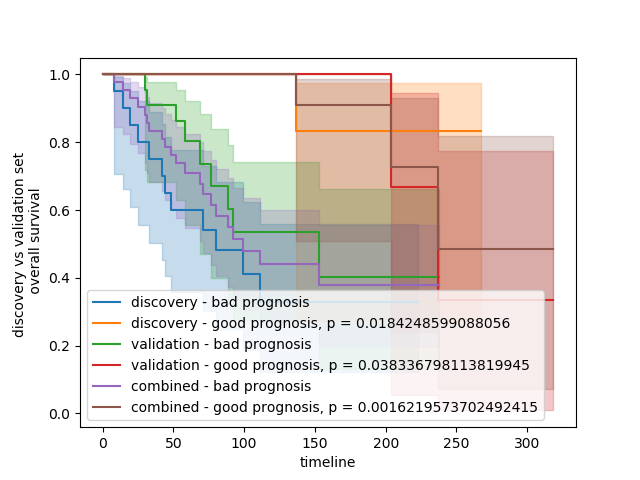

Supplement: Supplementary file 2 — interim classifiers [file 44303_2024_22_MOESM2_ESM.zip › classifier_0061.png]

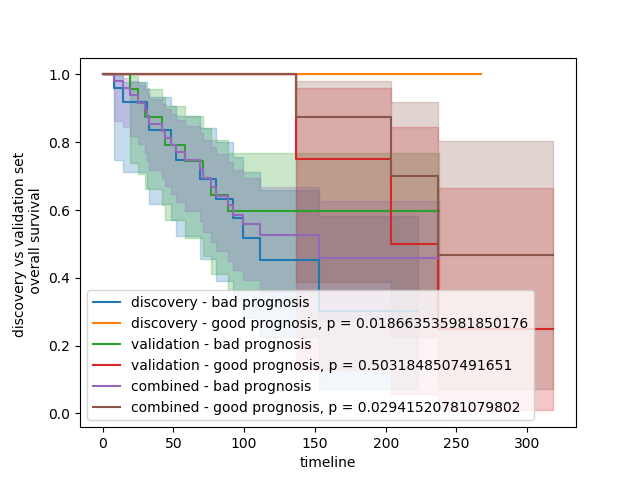

Supplement: Supplementary file 2 — interim classifiers [file 44303_2024_22_MOESM2_ESM.zip › classifier_0062.png]

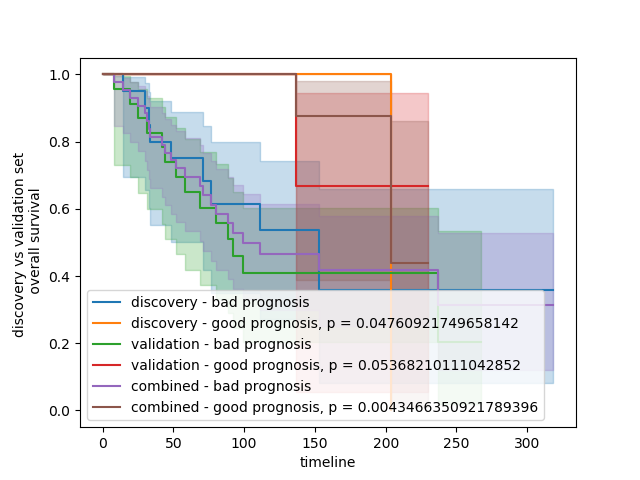

Supplement: Supplementary file 2 — interim classifiers [file 44303_2024_22_MOESM2_ESM.zip › classifier_0063.png]

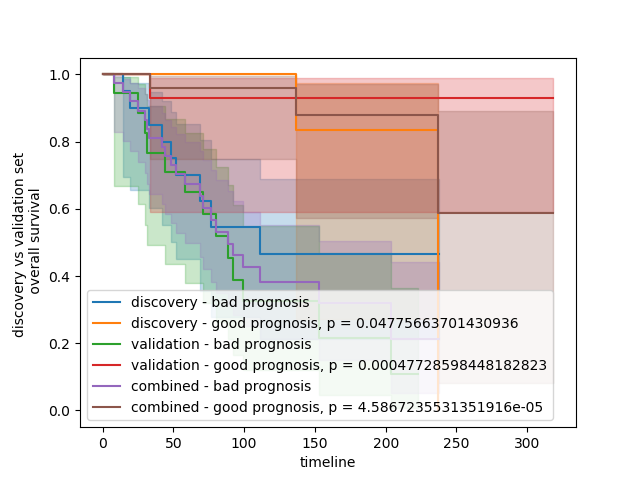

Supplement: Supplementary file 2 — interim classifiers [file 44303_2024_22_MOESM2_ESM.zip › classifier_0064.png]

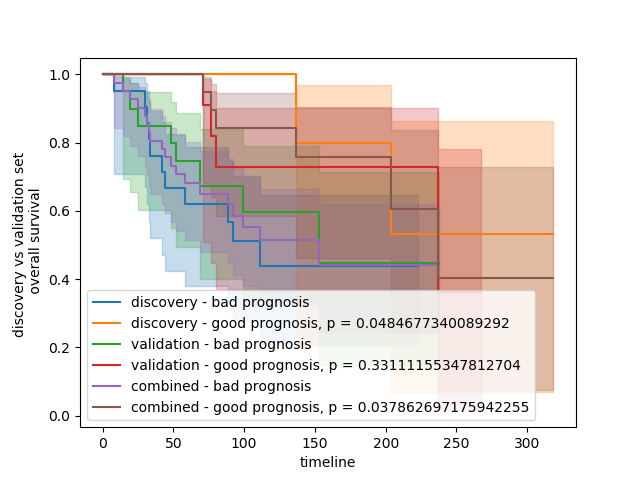

Supplement: Supplementary file 2 — interim classifiers [file 44303_2024_22_MOESM2_ESM.zip › classifier_0065.png]

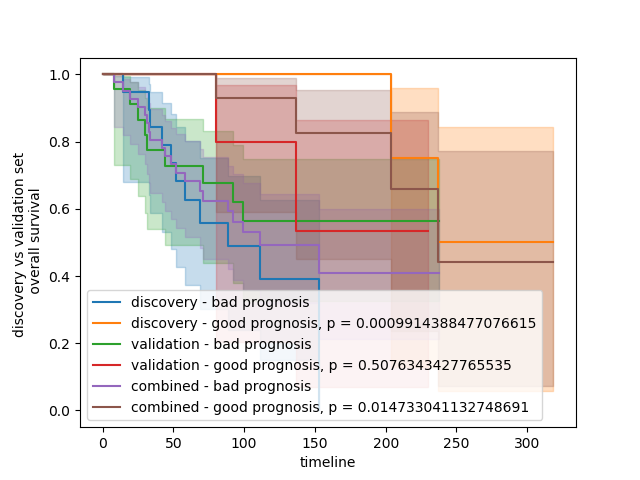

Supplement: Supplementary file 2 — interim classifiers [file 44303_2024_22_MOESM2_ESM.zip › classifier_0066.png]

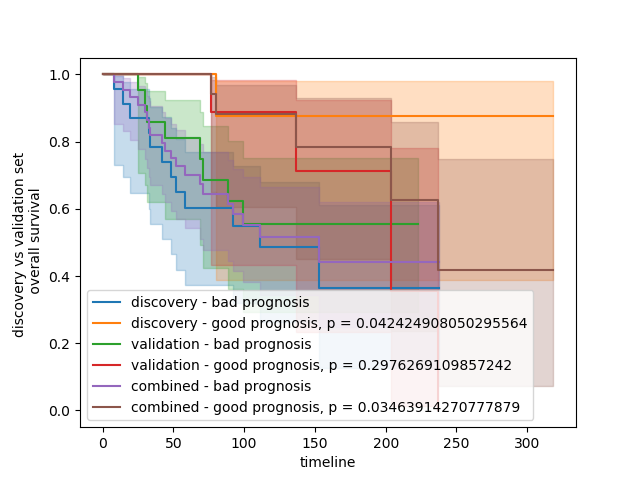

Supplement: Supplementary file 2 — interim classifiers [file 44303_2024_22_MOESM2_ESM.zip › classifier_0067.png]

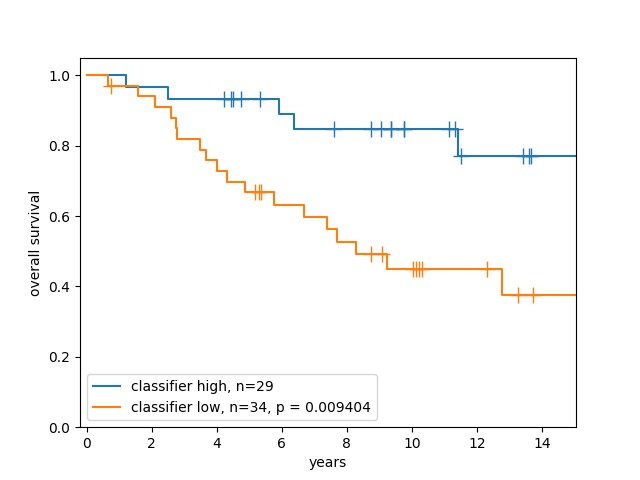

Supplement: Supplementary file 10 — varying parameter classifier performance [file 44303_2024_22_MOESM10_ESM.zip › top_1_keys_discovery.png]

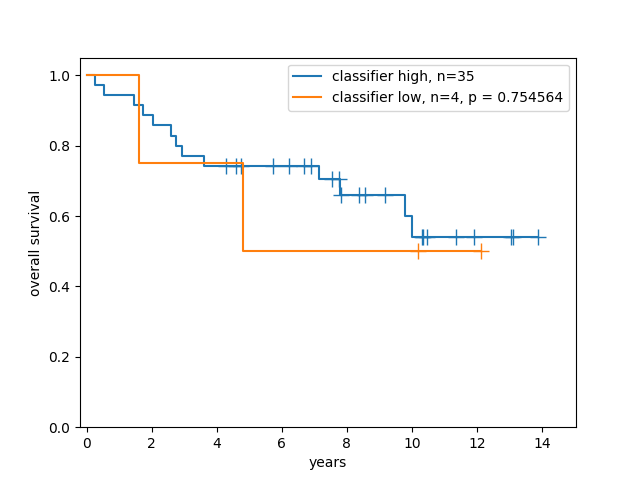

Supplement: Supplementary file 10 — varying parameter classifier performance [file 44303_2024_22_MOESM10_ESM.zip › top_1_keys_validation.png]

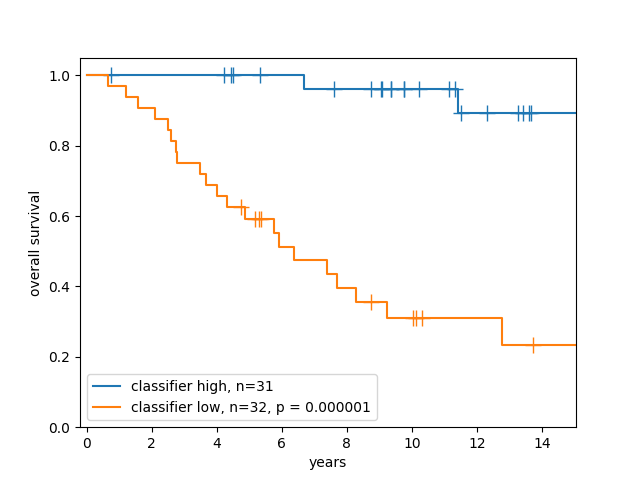

Supplement: Supplementary file 10 — varying parameter classifier performance [file 44303_2024_22_MOESM10_ESM.zip › top_10_keys_discovery.png]

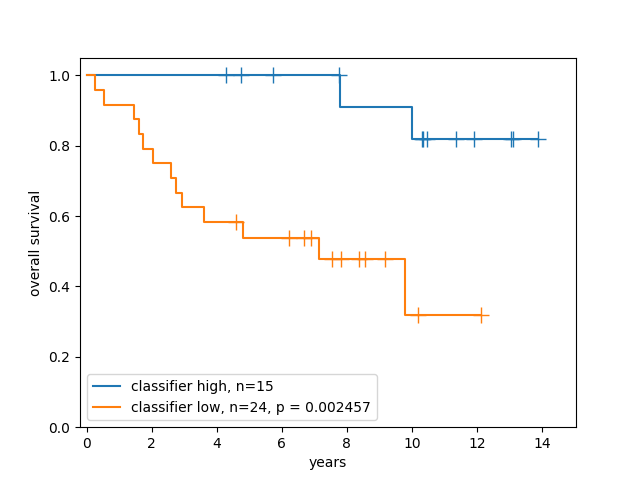

Supplement: Supplementary file 10 — varying parameter classifier performance [file 44303_2024_22_MOESM10_ESM.zip › top_10_keys_validation.png]

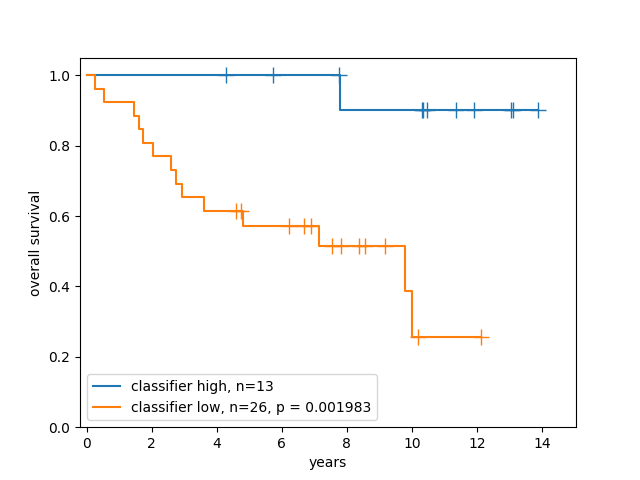

Supplement: Supplementary file 10 — varying parameter classifier performance [file 44303_2024_22_MOESM10_ESM.zip › top_11_keys_validation.png]

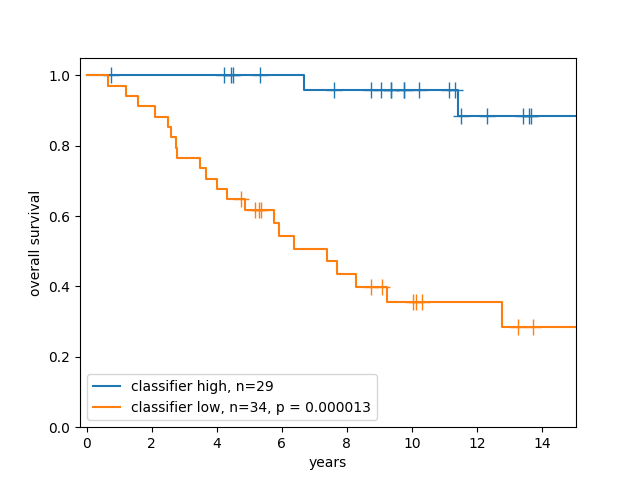

Supplement: Supplementary file 10 — varying parameter classifier performance [file 44303_2024_22_MOESM10_ESM.zip › top_12_keys_discovery.png]

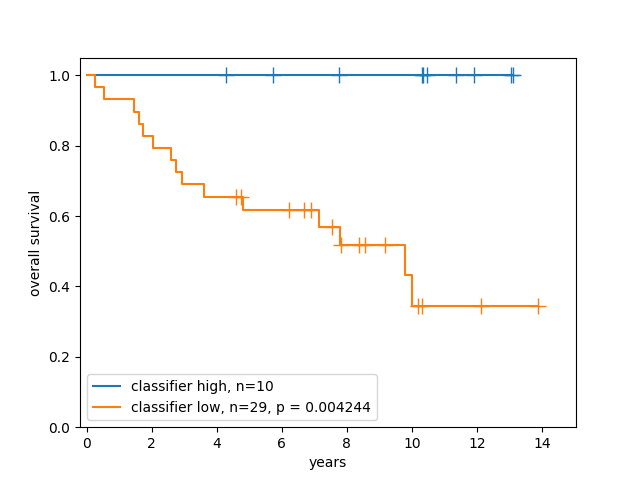

Supplement: Supplementary file 10 — varying parameter classifier performance [file 44303_2024_22_MOESM10_ESM.zip › top_12_keys_validation.png]

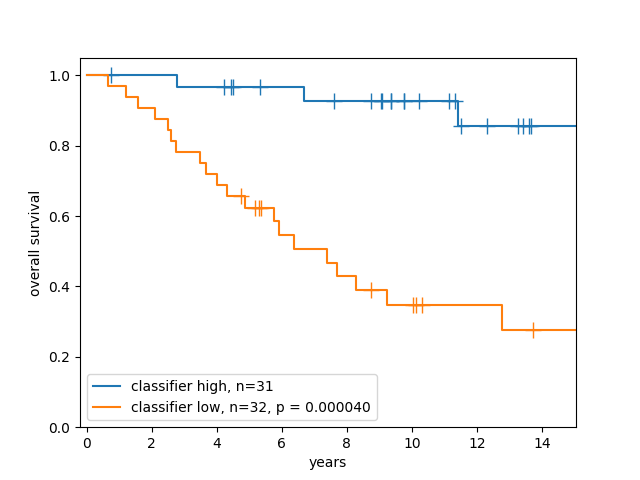

Supplement: Supplementary file 10 — varying parameter classifier performance [file 44303_2024_22_MOESM10_ESM.zip › top_13_keys_discovery.png]

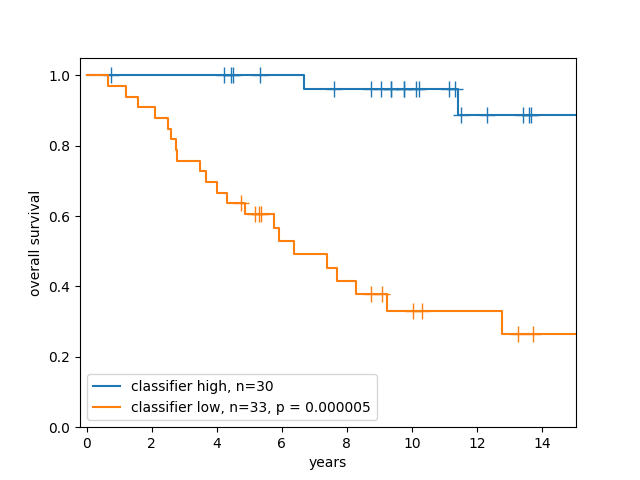

Supplement: Supplementary file 10 — varying parameter classifier performance [file 44303_2024_22_MOESM10_ESM.zip › top_14_keys_discovery.png]

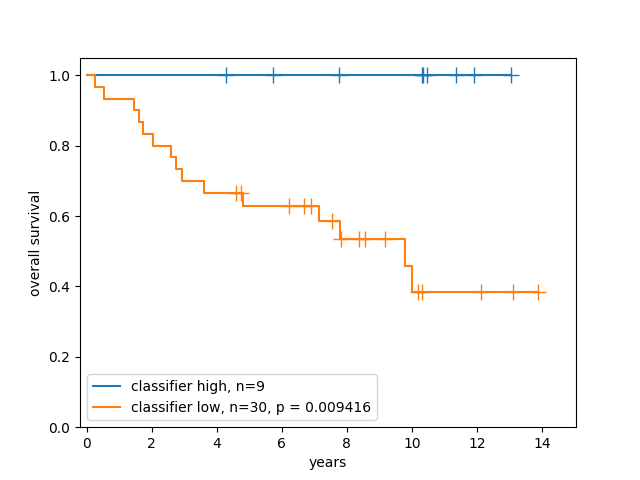

Supplement: Supplementary file 10 — varying parameter classifier performance [file 44303_2024_22_MOESM10_ESM.zip › top_14_keys_validation.png]

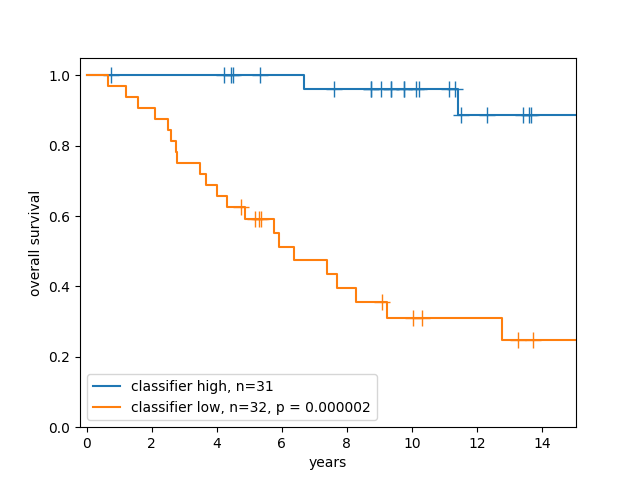

Supplement: Supplementary file 10 — varying parameter classifier performance [file 44303_2024_22_MOESM10_ESM.zip › top_15_keys_discovery.png]

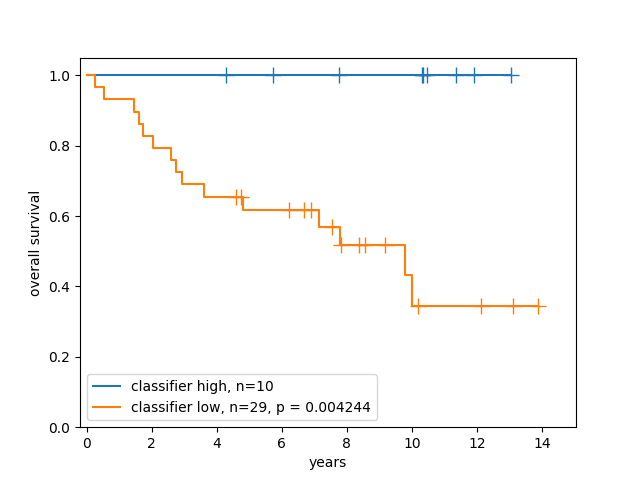

Supplement: Supplementary file 10 — varying parameter classifier performance [file 44303_2024_22_MOESM10_ESM.zip › top_15_keys_validation.png]

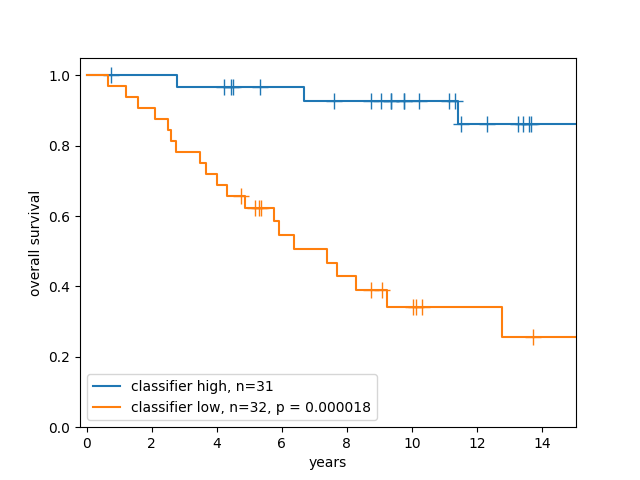

Supplement: Supplementary file 10 — varying parameter classifier performance [file 44303_2024_22_MOESM10_ESM.zip › top_16_keys_discovery.png]

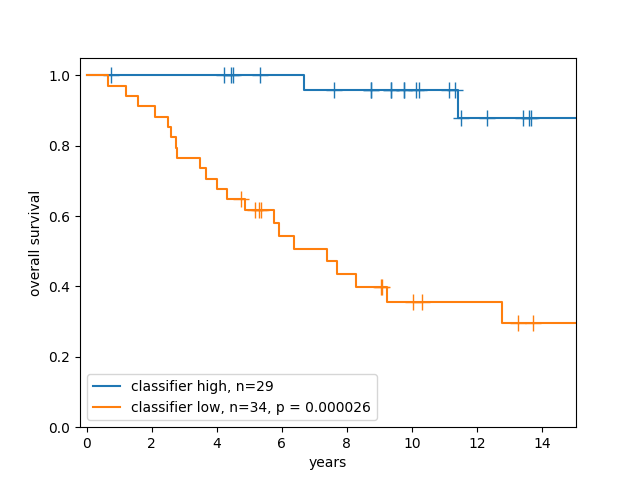

Supplement: Supplementary file 10 — varying parameter classifier performance [file 44303_2024_22_MOESM10_ESM.zip › top_17_keys_discovery.png]

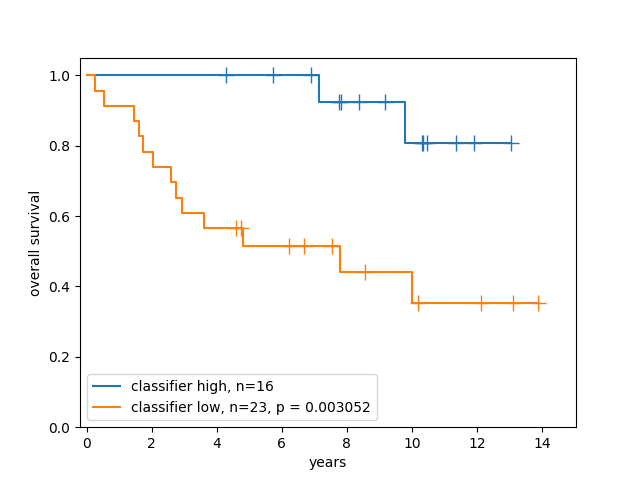

Supplement: Supplementary file 10 — varying parameter classifier performance [file 44303_2024_22_MOESM10_ESM.zip › top_17_keys_validation.png]

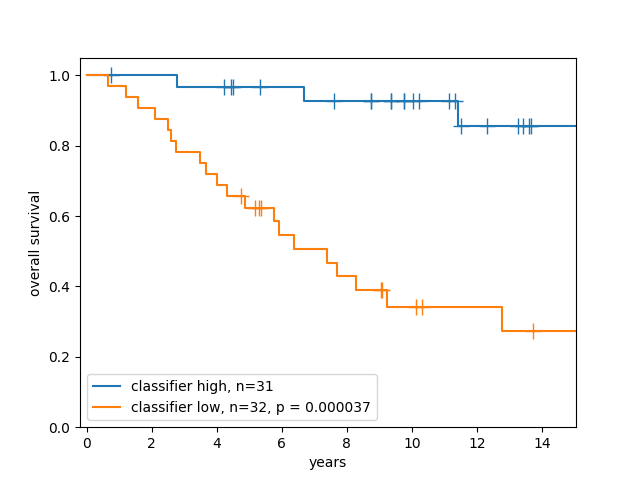

Supplement: Supplementary file 10 — varying parameter classifier performance [file 44303_2024_22_MOESM10_ESM.zip › top_18_keys_discovery.png]

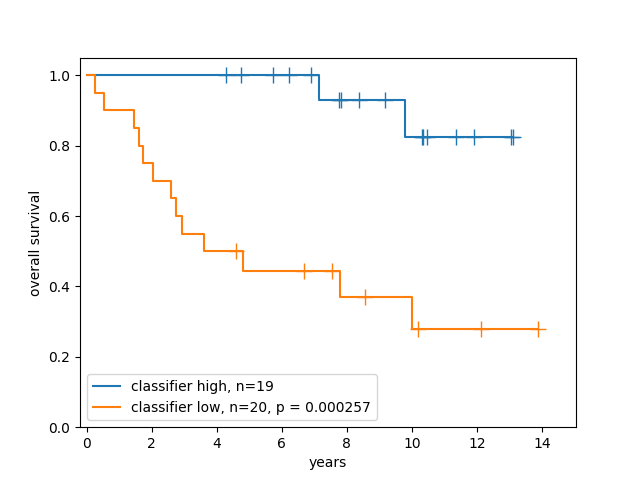

Supplement: Supplementary file 10 — varying parameter classifier performance [file 44303_2024_22_MOESM10_ESM.zip › top_19_keys_validation.png]

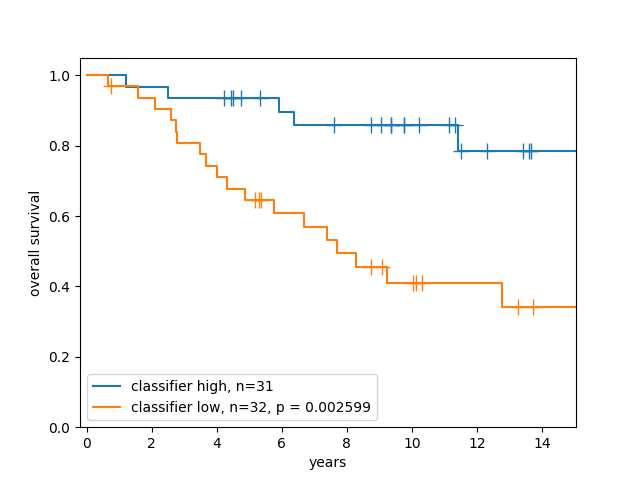

Supplement: Supplementary file 10 — varying parameter classifier performance [file 44303_2024_22_MOESM10_ESM.zip › top_2_keys_discovery.png]

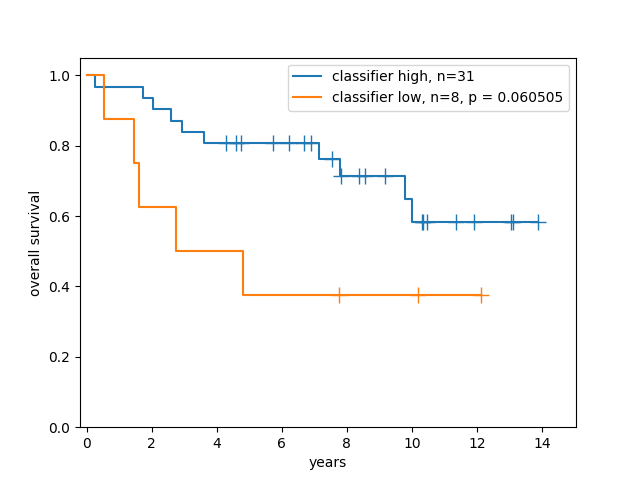

Supplement: Supplementary file 10 — varying parameter classifier performance [file 44303_2024_22_MOESM10_ESM.zip › top_2_keys_validation.png]

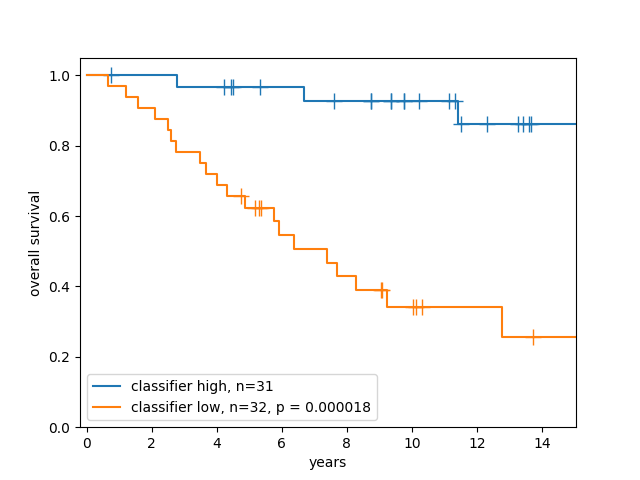

Supplement: Supplementary file 10 — varying parameter classifier performance [file 44303_2024_22_MOESM10_ESM.zip › top_20_keys_discovery.png]

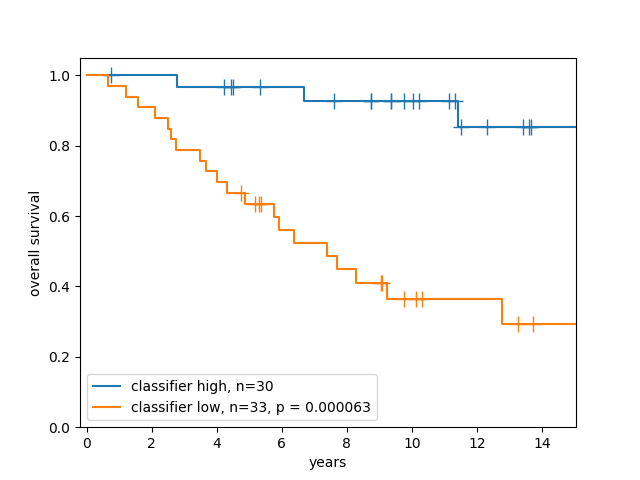

Supplement: Supplementary file 10 — varying parameter classifier performance [file 44303_2024_22_MOESM10_ESM.zip › top_21_keys_discovery.png]

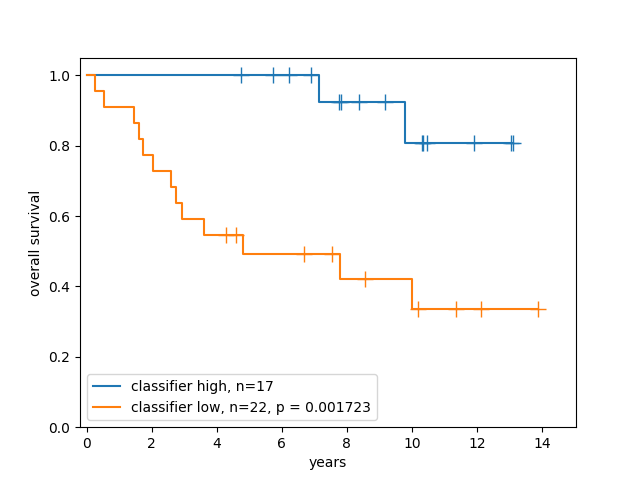

Supplement: Supplementary file 10 — varying parameter classifier performance [file 44303_2024_22_MOESM10_ESM.zip › top_21_keys_validation.png]

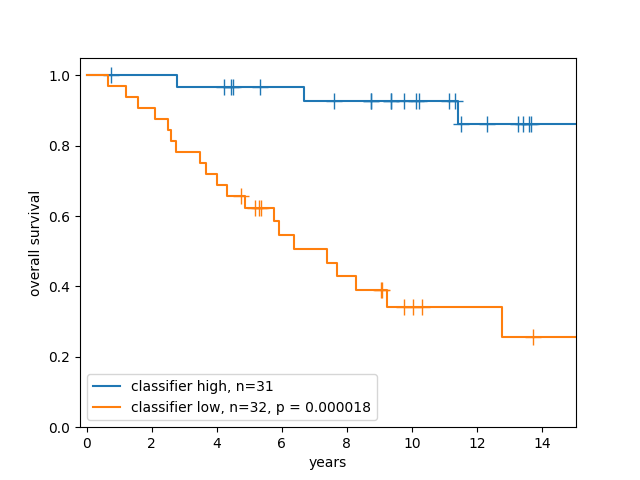

Supplement: Supplementary file 10 — varying parameter classifier performance [file 44303_2024_22_MOESM10_ESM.zip › top_22_keys_discovery.png]

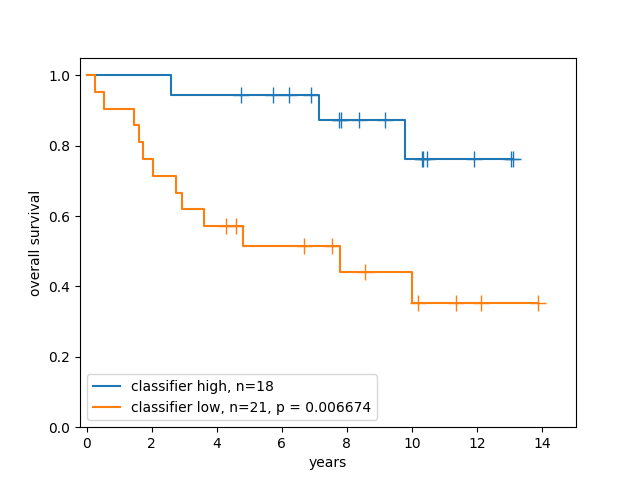

Supplement: Supplementary file 10 — varying parameter classifier performance [file 44303_2024_22_MOESM10_ESM.zip › top_22_keys_validation.png]

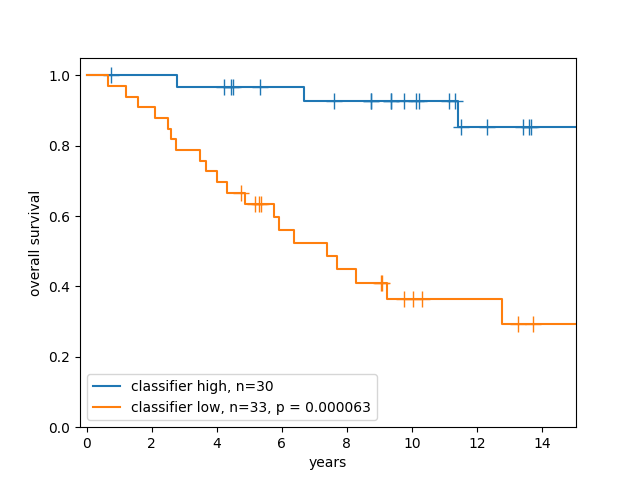

Supplement: Supplementary file 10 — varying parameter classifier performance [file 44303_2024_22_MOESM10_ESM.zip › top_23_keys_discovery.png]

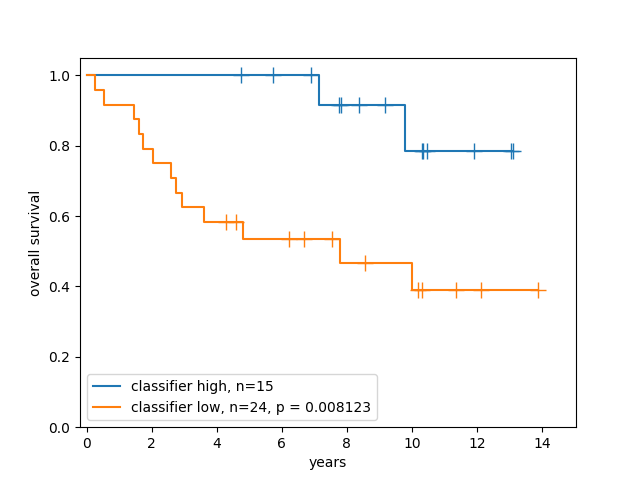

Supplement: Supplementary file 10 — varying parameter classifier performance [file 44303_2024_22_MOESM10_ESM.zip › top_23_keys_validation.png]

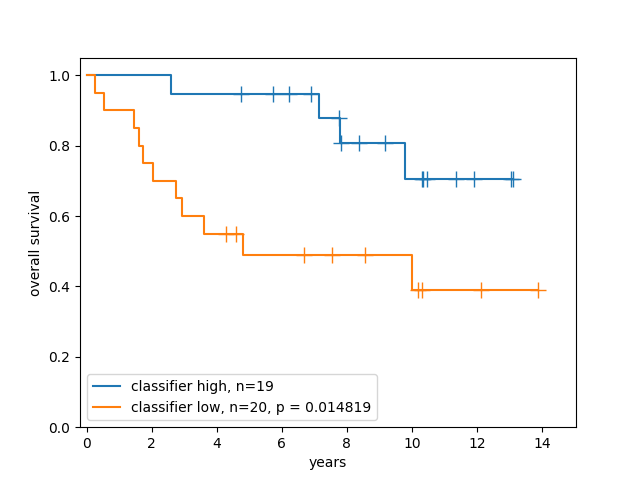

Supplement: Supplementary file 10 — varying parameter classifier performance [file 44303_2024_22_MOESM10_ESM.zip › top_24_keys_validation.png]

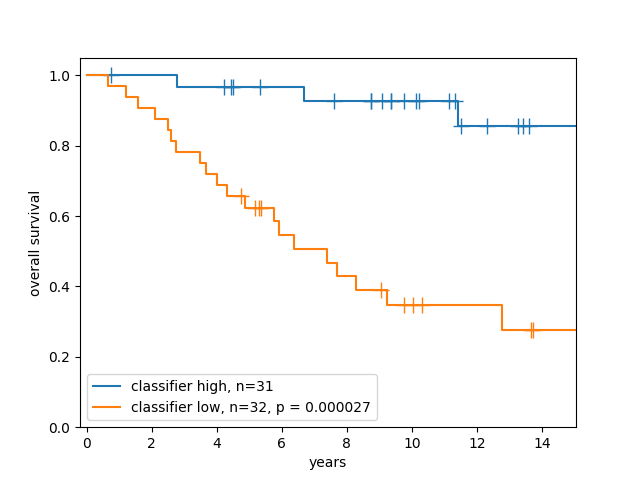

Supplement: Supplementary file 10 — varying parameter classifier performance [file 44303_2024_22_MOESM10_ESM.zip › top_25_keys_discovery.png]

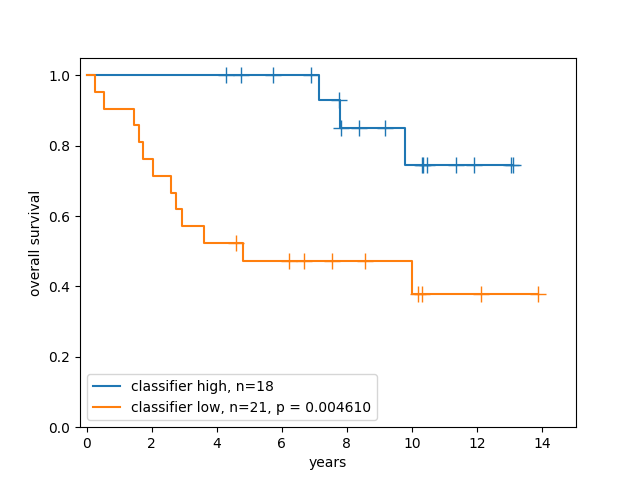

Supplement: Supplementary file 10 — varying parameter classifier performance [file 44303_2024_22_MOESM10_ESM.zip › top_25_keys_validation.png]

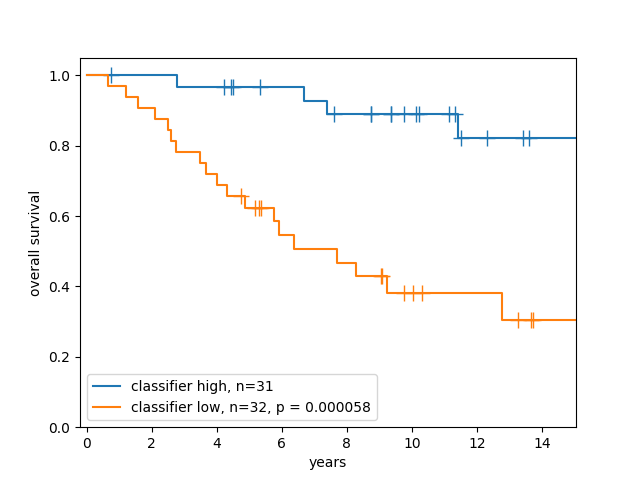

Supplement: Supplementary file 10 — varying parameter classifier performance [file 44303_2024_22_MOESM10_ESM.zip › top_26_keys_discovery.png]

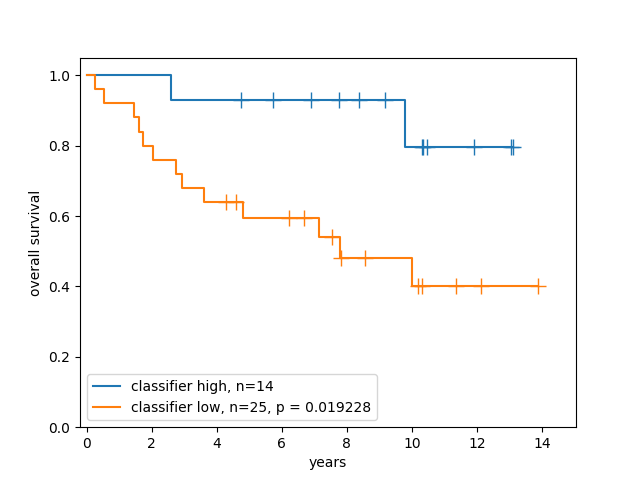

Supplement: Supplementary file 10 — varying parameter classifier performance [file 44303_2024_22_MOESM10_ESM.zip › top_26_keys_validation.png]

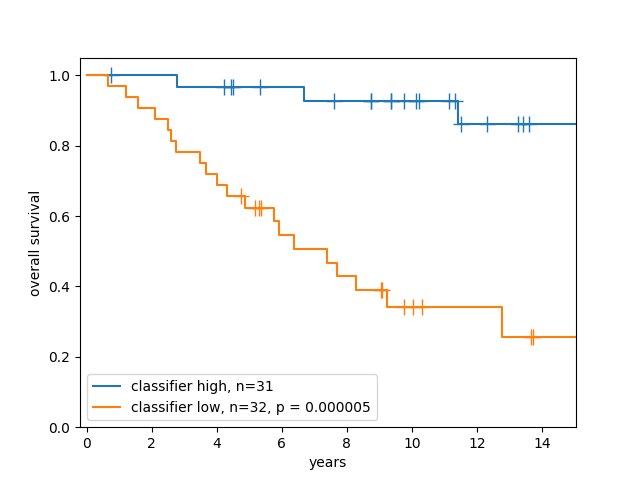

Supplement: Supplementary file 10 — varying parameter classifier performance [file 44303_2024_22_MOESM10_ESM.zip › top_27_keys_discovery.png]

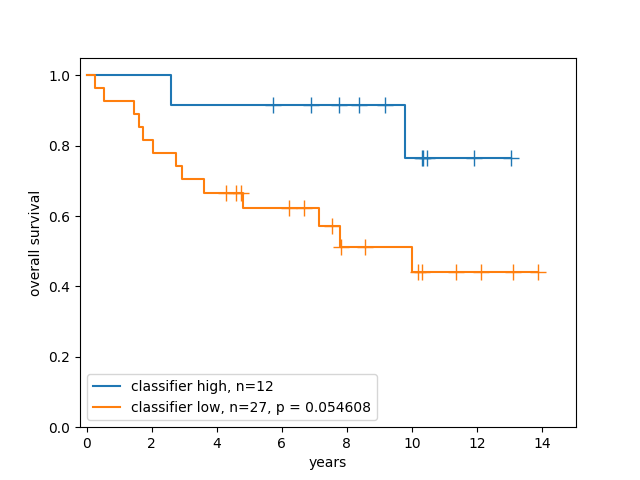

Supplement: Supplementary file 10 — varying parameter classifier performance [file 44303_2024_22_MOESM10_ESM.zip › top_27_keys_validation.png]
