# Supplementary material for: TME-analyzer: a new interactive and dynamic image analysis tool that identified immune cell distances as predictors for survival of triple negative breast cancer patients
Source: Npj Imaging. 2024 Jul 25;2:21. doi: 10.1038/s44303-024-00022-6 (PMC12118654; doi:10.1038/s44303-024-00022-6)
Supplement: Supplementary file 3 — Sample images, analysis tutorial and a sample analysis [file 44303_2024_22_MOESM3_ESM.zip › Sample images, analysis tutorial and a sample analysis/Image analysis tutorial.pdf]

Image analysis tutorial

Channel setup

By default, TME-A names image channels as C1, C2, etc. when you open an image with it. The channel setup should be input to the software before loading of images in order to have the proper naming of the channels, together with the display colors. This is achieved by going to `Loading -> Change Channel Setup`, which opens a new window. Here you should see a dropdown that is set to Default, with no other options present. By pressing `Add Channels Setup`, you can setup your own channel names, which will appear in this dropdown. For the sample analysis, press `Add Channel` 7 times (so the total number of channels are 8). And name them in the order of "DAPI", "CD8", "CD20", "CD3", "CD68", "CD56", "CK" and "background". The corresponding colors used in the manuscript, which can be selected from the dropdown, are blue, red, yellow, green, orange, magenta, cyan and black.

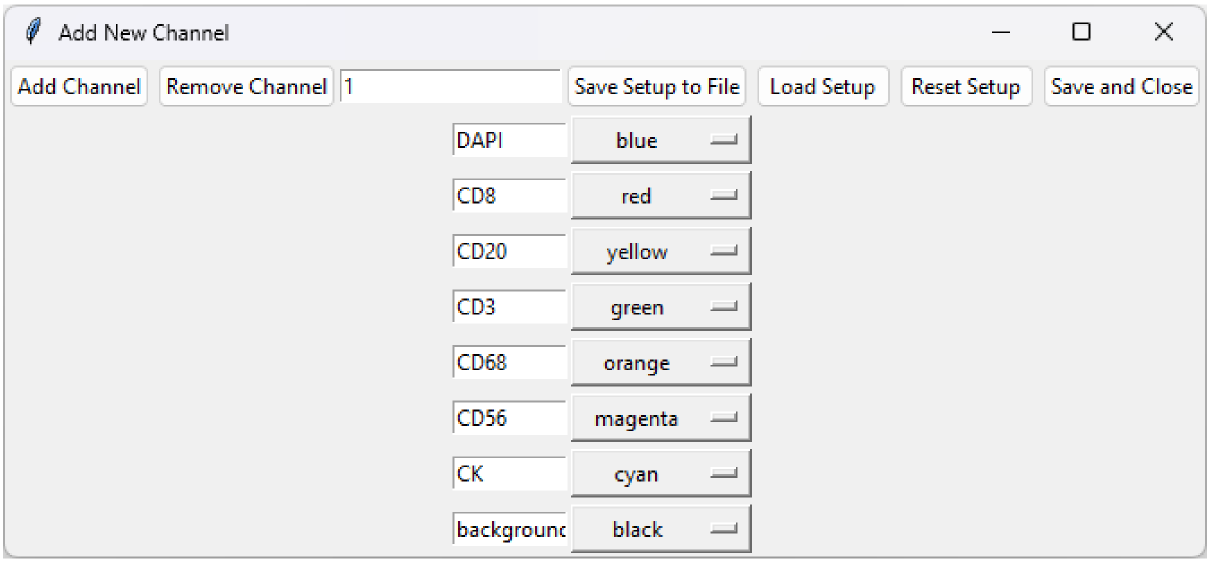

You can save this channel setup by clicking `Save Setup to File`, or load saved setup by clicking `Load Setup`. Once done, press `Save and Close`.

Now this setup should appear in the dropdown as: "DAPI:blue, CD8:red, CD20:yellow, CD3:green, CD68:orange, CD56:magenta, CK:cyan, background black"

Alternatively, if you press `Load default channels`, again a new dropdown option will appear: "TNBC: DAPI:blue, CD8:red, CD20:yellow, CD3:green, CD68:orange, CD56:magenta, CK:cyan"

Select the channel setup of preference, and press confirm.

Image loading

You can open image loading window by going through `Loading -> Load Image` or directly pressing the `Load Image` in the toolbar. This allows you to load multiple tiff images.

Go ahead and load the provided image, "sample image (1).tif". This should now provide you with a list of channels and colors at the right, with `Display` button at the bottom. Press `Display` to see the image you just loaded. By clicking/unclicking the checkboxes you can quickly turn individual channels on/off. Here, under the `Display` button, you also find a dropdown menu, where currently the option is "sample image (1).tif". As you load more images they are appended to this dropdown menu, and here you can change the image you are currently working on.

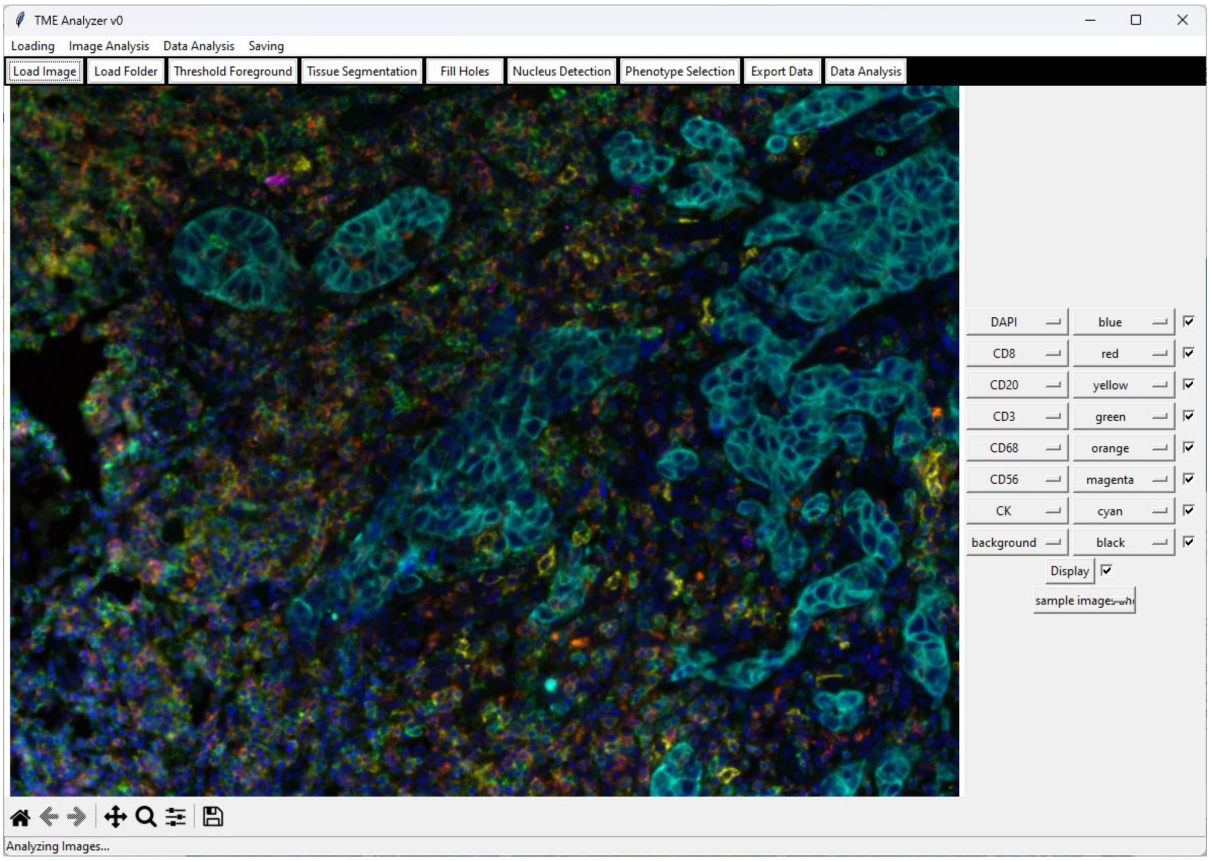

Load Folder

`Load Folder` is specifically designed for loading outputs of Vectra imaging, where images with "_component_data.tif" ending are loaded, with the options of combining images, stitching them (based on their coordinates outputted from the Vectra imaging platform) and downscaling.

Threshold Foreground

The first step of the image analysis is to discriminate the tissue from non tissue. You can reach the foreground thresholding through `Image Analysis -> Threshold` Foreground, or by clicking `Threshold Foreground` in the toolbar.

This will open a new window with the name Foreground Selection, where on the left you see the image, and the right a histogram, belonging to the first channel, and under it a number of options and levels. The histogram is clickable and allows you to update the thresholds for foreground. At the top in the toolbar, you also see a `Adaptive Threshold` preceded by an unclicked checkbox and a default value of 100. Now change this value to 1000 and click the

checkbox. This should change the histogram displayed at the right, but not affect the image. What this option does is it subtracts the original image uniform filtered by the value displayed here from the original image. This is the background correction that is used through our analysis. Whenever you change the size of the `Adaptive Threshold` you need to unclick and click the checkbox again to activate the new size.

Now that you know how to generate histograms of background corrected image, let's assign some thresholds. When you hover in the histogram area, you can see the location of your cursor at the top of the histogram. The x-values will be the thresholds selected. You assign the thresholds by click-and-drag on the histogram. When you do so, you will realize that the image will change, where the non-tissue will be displayed grayish, and tissue display will remain the same. Once you are satisfied with the foreground of a selected channel, you can press `Add Mask` at the right, and this will then show the range of the histogram you have selected. Note that if the upper boundary of the range you selected is higher than the maximum value in the image, infinity will be assigned to the upper boundary instead. If you are not happy with the channel selected, you can always press `Remove Mask`, which will remove the current channel. You can switch between channels by the dropdown menu under the histogram. Note that when the channel is changes, it will change also the `Adaptive Threshold` to the saved settings for individual channels. In case there was no prior saving, this means the `Adaptive Threshold` will be set to 100 and turned off.

TIP: If you want to assign a minimal value for the threshold, without assigning a maxima, after click-and-drag, double click with the mouse. Now when you press `Add Mask`, you will see that the maximum value does not change. Here, if you already have a range selected, the maximum again does not change and remains the previous assigned value. If you want the upper boundary to be infinity instead, you will first have to click `Remove Mask` before the click-drag-double click sequence.

Reproduction of the analysis in the manuscript

1. Change the channel to "DAPI".
2. Change the `Adaptive Threshold` to 1000.
3. Click on the checkbox next to `Adaptive Threshold` (or unclick and click).
4. click-and-drag the mouse at the histogram, aiming for a value around 0.1, and release, double-click. You will see the image change.
5. Press `Add Mask` to add the mask corresponding to DAPI to the Foreground. You will see the numbers next to the blue-DAPI change from "0.0 - inf" to the selected threshold values.

Repeat steps 1-5 for CD8, CD20, CD3, CD68, CD56 and CK. If the values you observe next to a channel differentiate significantly from "0.1 - inf", select this channel, press `Remove Mask` and repeat steps 1-5 for this channel. When all channels are assigned, press Show Joint Mask to see your foreground mask. This is then all the masks added together. So signals in the background corrected image of any channel higher than the assigned threshold value (which should be around 0.1) is now a part of the foreground mask

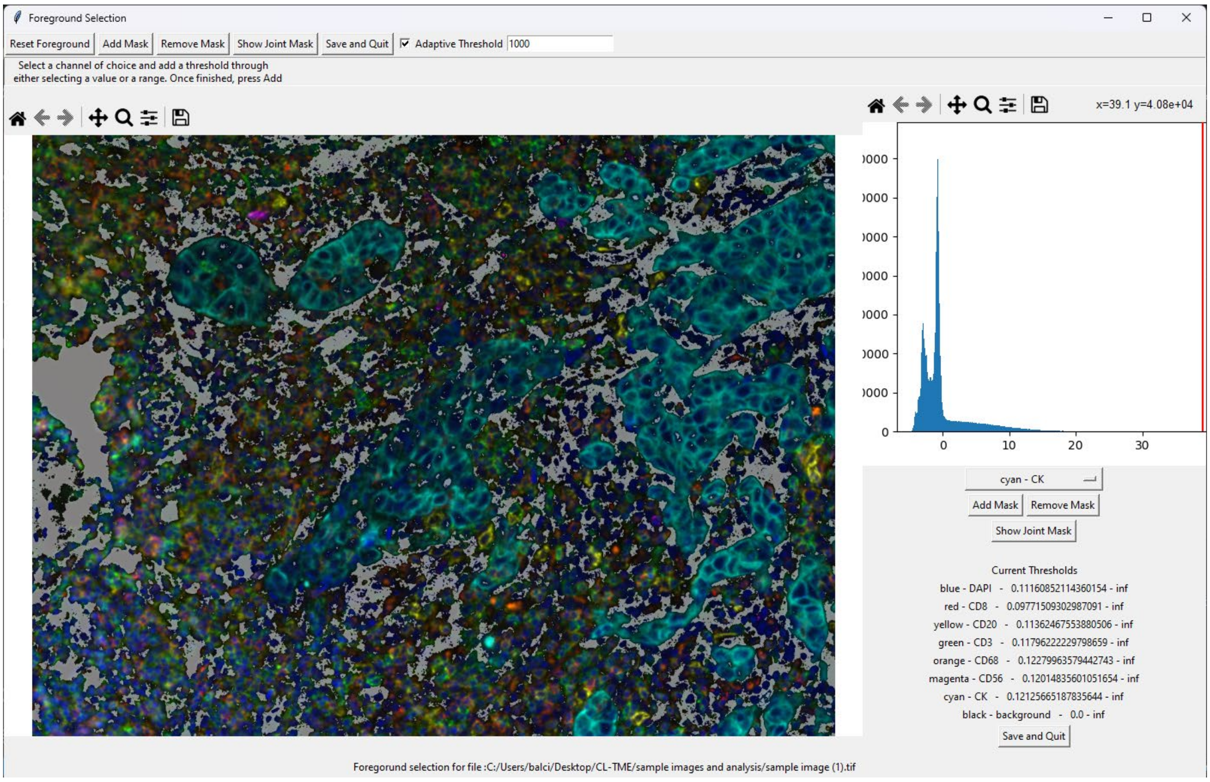

When satisfied, press `Save and Quit` to save and close the Threshold Foreground, and return to the main image analysis window. With a new "channel" with the name Foreground. Here you can visualize the selected foreground.

Foreground gap-filling

In the foreground image you just created, you will realize a lot of small regions assigned to non-foreground. You will now correct this by "Filling Holes". You can reach this option through `Image Analysis -> Fill Holes` or by clicking `Fill Holes` in the toolbar. This will now open the new window titled "Fill Images" with a yellow (foreground) - purple (background) image in the left with a dropdown at top right displaying "Foreground" and two histograms at the right with titles "Foreground Area Limits" at top and "Background Area Limits" at the bottom, with the ranges indicated. Here, on both the histograms, click-and-drag to select the range from around 5000 until the upper range of the histogram. You will realize the image, the histograms and the range at the histogram title changing when you do so. Again note that if the upper limit of your selected range is larger than the image size, then the upper limit is set to the default value of infinity. When you are satisfied with the result, click `Register Filled Image` at the left top to overwrite your Foreground image.

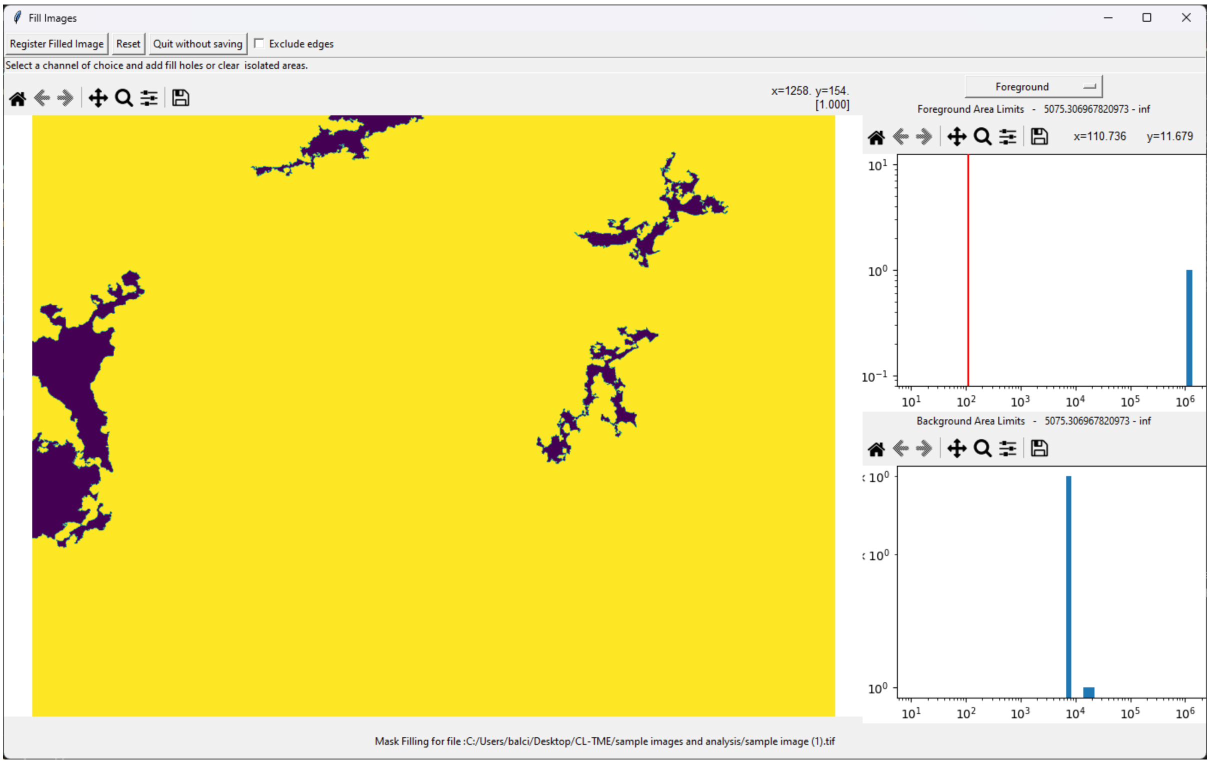

Image filtering

Go to `Image Analysis -> Filter Image` to open the image filtering module. Here you can observe how different filters and analytical/logical operations change the image. You can add the filtered image as a new channel through `Add Image`, overwrite the current channel through `Overwrite Image`, use the image only for filtration by clicking `Use for Phenotyping`, or remove a previous filter through `Remove Image`.

Gaussian filtering of CK signal

Prior to tissue segmentation, we should register (i.e., `Add Image`) the gaussian filtered CK channel for tumor segmentation. First change the channel to CK by changing the second from right dropdown at the top from "DAPI" to "CK". To facilitate the observation in real-time the effect of this filtration, best to also change the top right dropdown from "Composite" to "white" or to a color of choice.

Leave the first dropdown at "Gaussian filter", change the number from 3 to 10, change the operation from "-" to "and-", the second filter from "Gaussian filter" to "Minimum" and the second number from 30 to 1000. Here the operation "and-" performs the second filter on the image that has already been processed with the first filter and then performs the subtraction of the second filtered image from the first; whereas the "-" operation performs both filters on the original image and then performs the subtraction. At this point, the image has not changed yet. You need to activate the individual filters by clicking the checkboxes. If the checkboxes are clicked while changing the filter type or size, the image is updated in real-time. Here, leave the checkbox after "Normalize" unclicked. This function normalizes the range of the filtered image so the minimal signal is 0 and the maximum is 1, which is not what we want for these images.

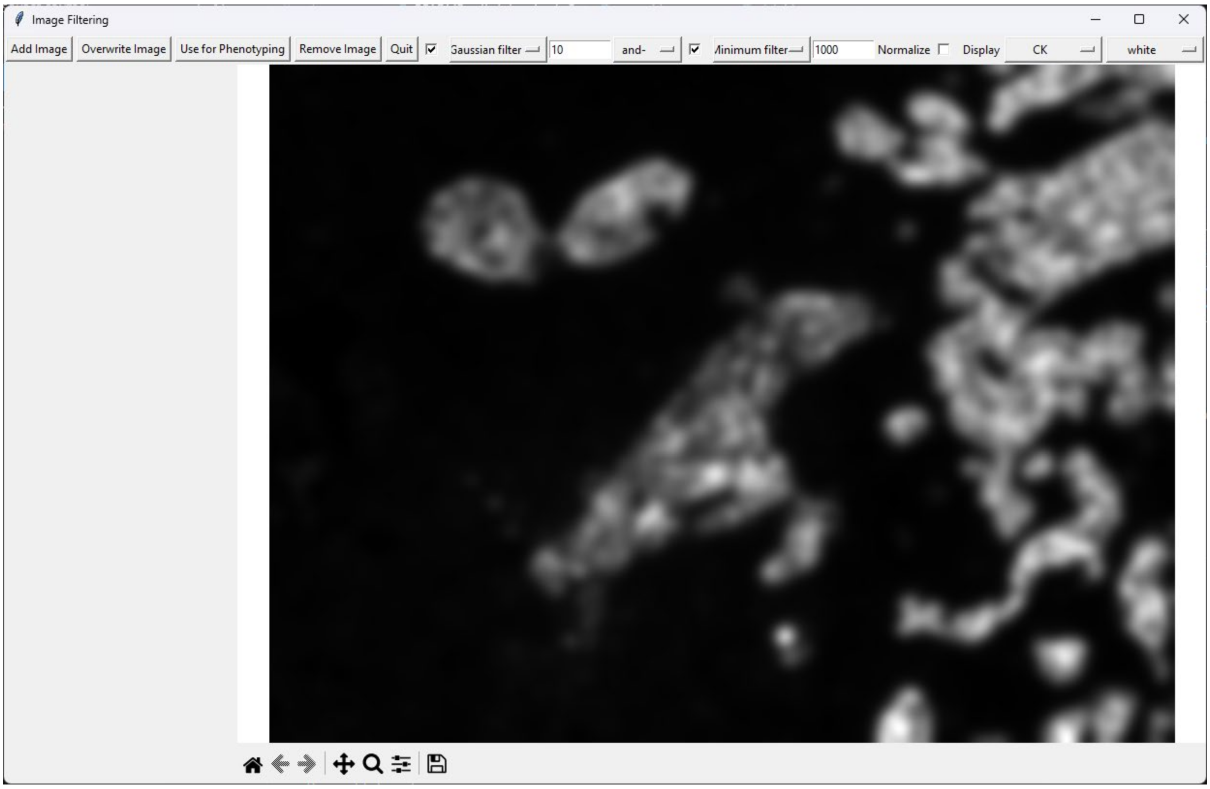

Once you are satisfied with the gaussian blurred CK signal for the tissue segmentation, press `Add Image` and press `Okay`. If you now look at the main image analysis window, you will see the new channel of CK_filtered appearing on the list of channels at the right.

Uniform background correction for phenotyping

While we are still at the filtering window, we can also register the background corrected images for phenotyping: Go through the channels of phenotyping (i.e., CD8, CD20, CD3, CD68, CD56 and CK).

1. Set the first filter to "Gaussian filter" with size 0.
2. Set the operator to "-"
3. Set the second filter to "Uniform filter" with size 1000.
4. Make sure the checkboxes next to the filter types are checked.
5. Make sure the checkbox next to "Normalize" is not checked.
6. Click `Use for Phenotyping` and press `Okay`

Note that once the parameters are setup for one channel, they do not need to be readjusted after changing the channel. You can directly change channel and press `Use for Phenotyping` and go through the channels.

Close the Image Filtering window

Tissue segmentation

You can open the Tissue Segmentation window either through `Image Analysis -> Tissue Segmentation` or by clicking `Tissue Segmentation` at the toolbar. You see a similar

window to the that of the Foreground Selection. Under the histogram, change the selected channel to the CK_filtered that we created in the previous step. You will then see a very clear separation of positive and negative signals in the histogram. Click-and-drag to make a selection from around 1 to he maximum of the x-axis and press `Add Channel`. You should see the range next to the CK_filtered change to 1 to infinity. Keep in mind that for upper limit of the range to go to infinity, the upper limit of your range selection has to higher than the highest value on the histogram.

Once satisfied, click `Add Segment`. Here in the new window titled "Add Segment" there is a dropdown menu with the names "DAPI", "Tumor", "Stroma" and "other". Select "Tumor" and press `Okay`.

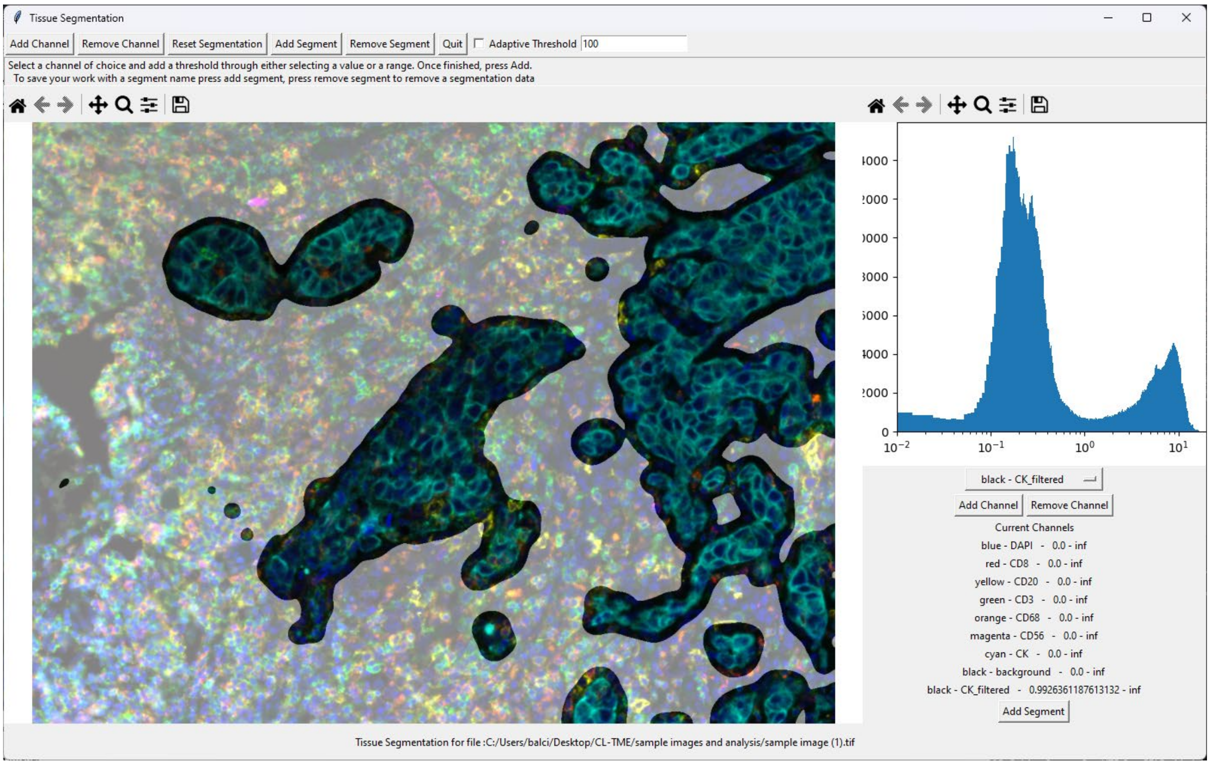

You may now close the segmentation window.

Additional notes on tissue segmentation

Here from the default names, "DAPI" mask generated is assumed to be nucleus signal for further analysis; whereas assigning a signal to "Tumor", if "Stroma" is left unassigned, generates a "Stroma" mask from Foreground mask that is not assigned to "Tumor" so that "Tumor" and "Stroma" masks are mutually exclusive and when combined would result in the Foreground mask. The inverse also happens if "Stroma" is assigned and "Tumor" is not.

Note: If range is selected in multiple channels, unlike the Foreground Selection where the signals are added resulting in a logical "OR" operation, here masks are multiplied resulting in an "AND" operation, so only the tissue that is positive for BOTH signals is assigned in the segmentation.

Tissue gap-filling

You can again reach the gap filling window through `Image Analysis -> Fill Holes` or by clicking `Fill Holes` in the toolbar. This will open the new window titled "Fill Images" with a yellow (foreground) - purple (background) image in the left with a dropdown at top right displaying "Foreground" and two histograms at the right with titles "Foreground Area Limits" at top and "Background Area Limits" at the bottom, with the ranges indicated. Change the image from "Foreground" to the newly assigned "Tumor" from the dropdown at the right top. Here, on both the histograms, click-and-drag to select the range from around 5000 until the upper range of the histogram. You will realize the image, the histograms and the range at the histogram title changing when you do so. Again note that if the upper limit of your selected range is larger than the image size, then the upper limit is set to the default value of infinity. When you are satisfied with the result, click `Register Filled Image` at the left top to overwrite your Tumor image.

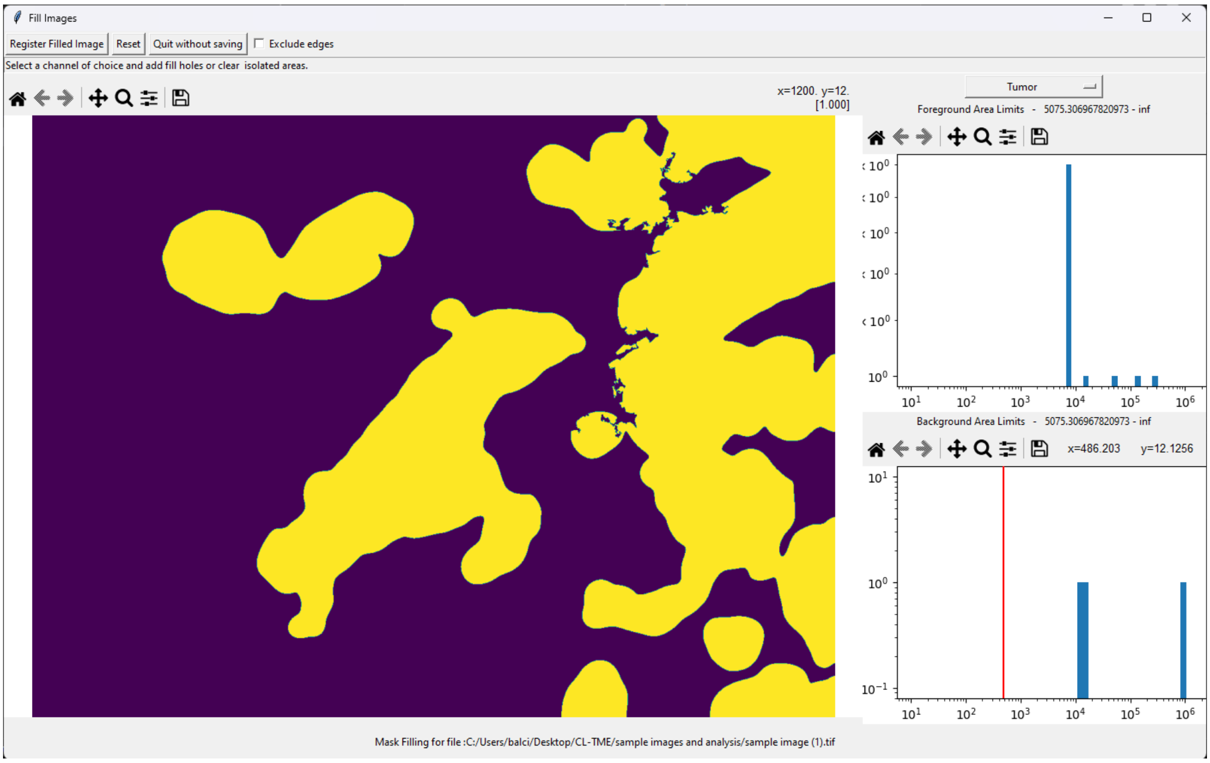

Cell segmentation

For cell segmentation, we will be using the stardist algorithm (https://github.com/stardist/stardist). You can access this algorithm in TME-A through `Image Analysis -> Neural Networks Segmentation`. This segmentation model is presented in a ready-to-apply mode, and you only need to press `Perform`. After this step, new images with the name DAPI, Nuclei and Cells should appear in the main window. Feel free to look through these images.

Notes on stardist segmentation

Relating to the options in the Neural Networks Segmentation

- If you unclick the checkbox next to Nucleus segmentation, you can choose the name your segmentation masks as you wish.

- Changing the Threshold would change the amount of signal assigned to nucleus, whereas non-maximum suppression would change the amount of segmentation. Further details can be found in the stardist website (https://github.com/stardist/stardist).
- "Extend Cell Area To:" assigns regions to cells, starting from the nucleus, within the foreground until either the value of the cell area reaches the indicated value, or the boundary of the cell reaches the boundary of the adjacent cell.
- If you change the "Extend Cell Area To:" dropdown to "Extend Cells:" assigns regions to cells, starting from the nucleus, within the foreground until either the distance from the nucleus boundary to the cell boundary reaches the indicated value, or the boundary of the cell reaches the boundary of the adjacent cell.
- Pressing `Preview` would show the segmentation in the main image window without saving it.

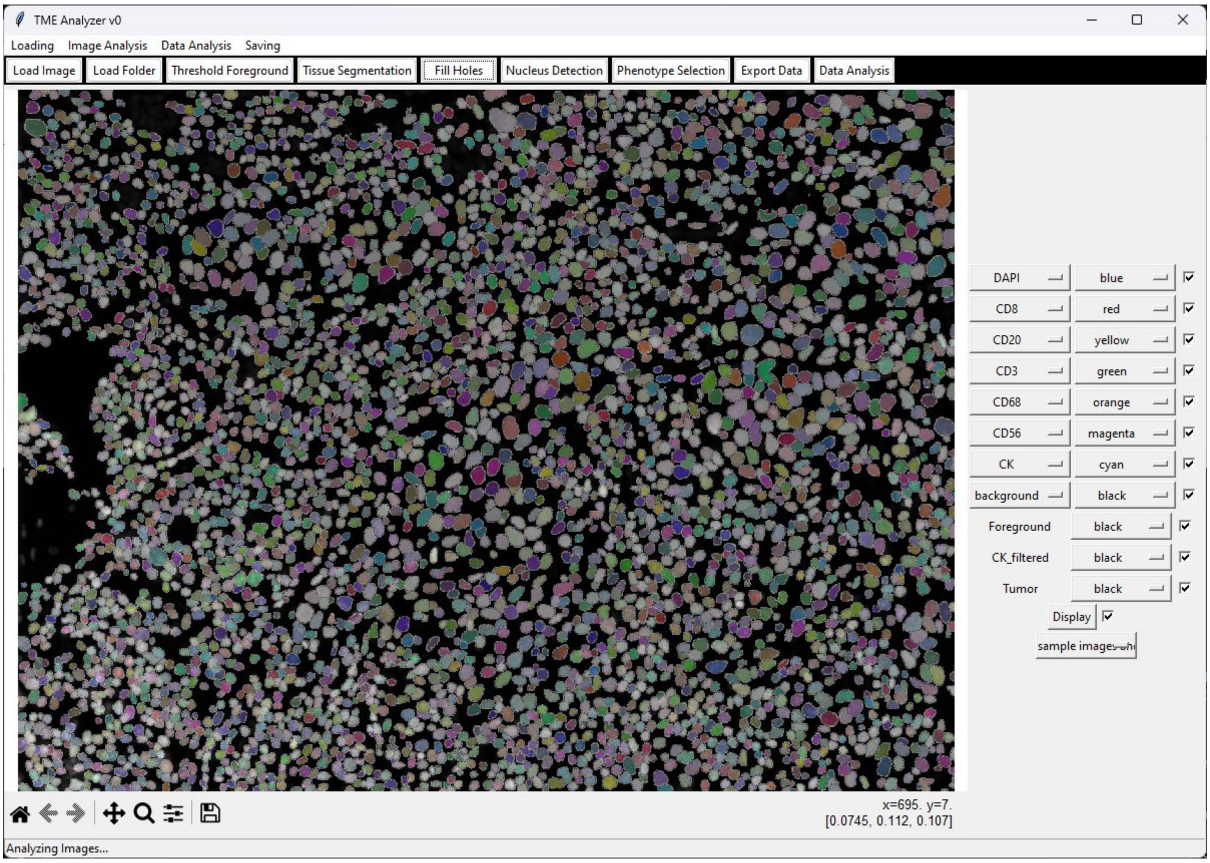

Alternative segmentation

Similar to assigning a Tumor mask, a DAPI mask can also be defined in the `Tissue Segmentation` window. Be warned that the stardist segmentation gives a more accurate segmentation than the manual segmentation. If you however want to explore this option, an adaptive threshold of 100, and minimum threshold of 1 works decent for manual DAPI mask. After setting up these values for the DAPI channel and pressing `Add Channel`, press `Add Segment` select "DAPI" and press `Okay`. At this step you can close the "Tissue Segmentation" window. If you now click the `Nucleus Detection` you can perform watershed segmentation based nucleus and cell segmentation. You can select the range of acceptable nucleus size from either of the histograms on the right. The top histogram displays the size of the DAPI mask before segmentation, and bottom is after. Note that the DAPI mask below the lower

range is just ignored. Here again the "Extend Cell Area To:" and "Extend Cells:" options are available at the right top. Additionally, there is a `Combine cells <` button at the toolbar. This button makes the software combine the cells of area smaller than the area indicated in the box (default set to 100). Again it should be noted that the stardist segmentation works better than the manual nucleus segmentation for this dataset.

Cell phenotyping

After segmentation of nuclei and cells, we can go ahead with phenotying the cells. If you press `Phenotype Selection` in the toolbar, which can also be found at `Data Analysis -> Phenotype Selection`, the cell intensity profiles, using the filtered images from image filtering above, will be calculated. In the new window, these values will be displayed as a 2D scatter plot in the center axis, the histogram of the x-axis parameter is shown at the top axis, whereas the histogram of the y-axis parameter is shown at the right axis. Here also note that in the original window now there is a new "All Cells" image, which corresponds to the nucleus centers of all cells. To set the values presented in the manuscript;

1. Change the second dropdown on the y-axis from "Nucleus" to "Cell"
2. Change the third dropdown on the y-axis from "Geometry" to "CD8" The first and last dropdowns are to be "Linear" and "Mean Intensity", which are also the default settings
3. Press the `Add Phenotype` button at the right, which activates the cursor to selection mode.
4. Drag the range at the right axis to get values higher than 0.4. NOTE: At this point you can turn on the `Show pop` option to see on the image in the main window the selected population (shown in red) and the non-selected population (shown in blue). For clear visualization, in the main window, you can unclick the check-box next to display to turn off all channels and click the check-box next to CD8 to only show this channel. These steps will overwrite the `Show pop` option, you can reactivate it by clicking it again in the "Phenotype Selection" window. Here you can also change the size of the cell location indicators by dragging the "Point Size" slider at the top.

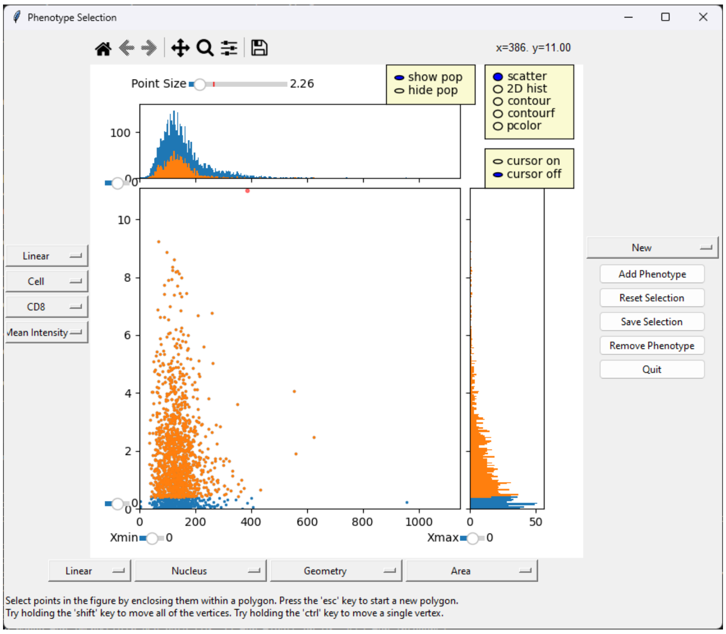

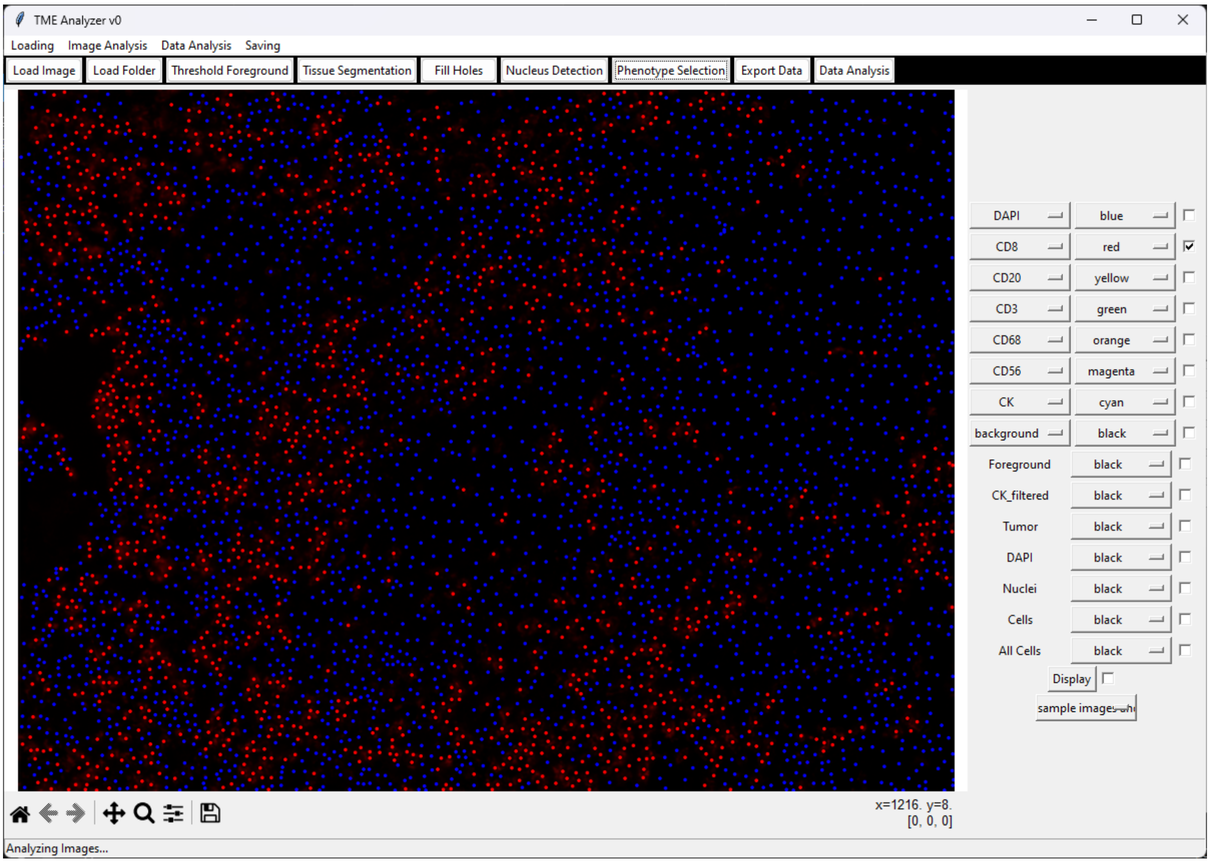

5. Once you are satisfied, press `Save Selection` on the right of the "Phenotype Selection" window.
6. Name your selection as "CD8+" and press `Okay`

Here note that, in the main window, in addition to the new CD8+ phenotype, also two phenotypes, namely "Tumor+" and "Stroma+" are defined. These are based on the tissue segmentation and segregates the cells, based on the location of the center of their nuclei, into tumor compartment and stroma compartment cells, the latter being defined as the non-tumor compartment cells.

Repeat steps 1-6 for CD20 (threshold of 0.7), CD3 (0.3), CD68 (1), CD56 (0.7) and CK (0.1), naming them CD20+, CD3+, CD68+, CD56+ and CK+ respectively.

At any point you can look at a particular phenotyping by selecting it at the dropdown menu at the right hand side of the "Phenotype Selection" window.

Once finished, close the phenotyping window. Now the image analysis is finished and your main window with the analysis images should like the following:

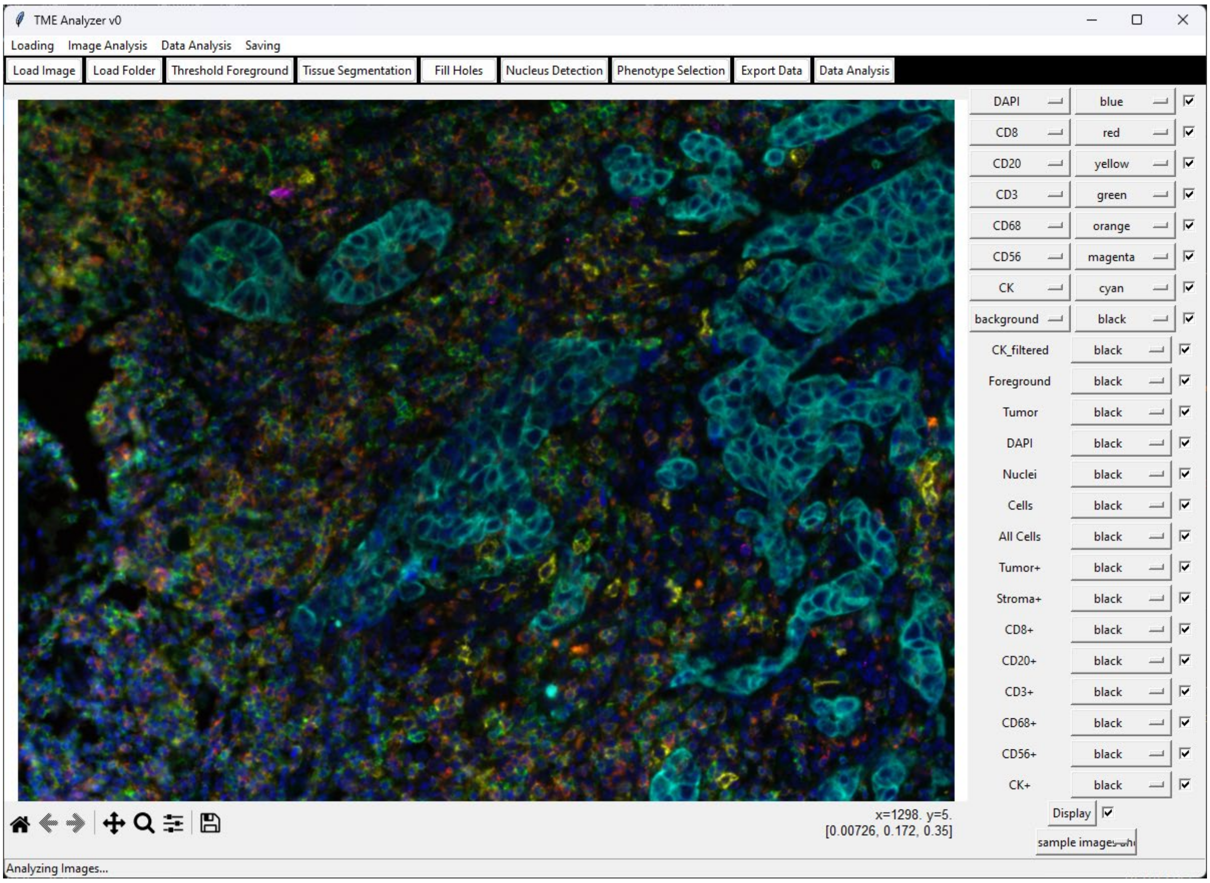

Cell phenotyping visualizations

This window is to facilitate the phenotype selection so feel free to play around with all the different buttons and drag controllers in the axis display to explore the possibilities.

Downstream analysis

It is always a good practice to save your analysis when finished, and reapply it. So let's look at these two options first

Saving analysis

You can save your image, analysis parameters and the analysis results by clicking `Saving -> Save Workplace`. This exports the image and analysis as a ".picle" file.

Loading analysis

You can load saved analysis through `Load -> Load Workspace`.

Reanalysis

You can see the analysis parameters that are used to copy an analysis from one image to another in the "Log". If you go to `Data Analysis -> Display Log` you can see the output from the python console being displayed here. This is also the place to look if software

freezes, or does not give the expected outcome, to see what step caused the error. In order to display the analysis parameters in this window, you just need to press the `Print params` button. The contents of the log remain even after closing and reopening it, therefore it continuously captures the console. You can however always press `Clear` to clear the contents of the log.

Reanalysis on same image

You just have to click `Data Analysis -> Redo Analysis` to perform the analysis with the saved setting fresh on the same image.

Applying current analysis to all open images (including the same image)

Here, you just have to click `Data Analysis -> Apply Analysis to All` to perform the analysis with the saved setting on all the open images.

Using a prior analysis to analyze current image

You can do this by pressing `Data Analysis -> Reanalyze Image Like ...`, which opens a new popup, allowing you to select which image analysis you want to copy (this analysis should be already loaded into TME-A. See Loading analysis). Here, you can also select the same image, which is the same as `Data Analysis -> Redo Analysis`.

Export Data

You can export the cell data from the software by pressing `Export Data`

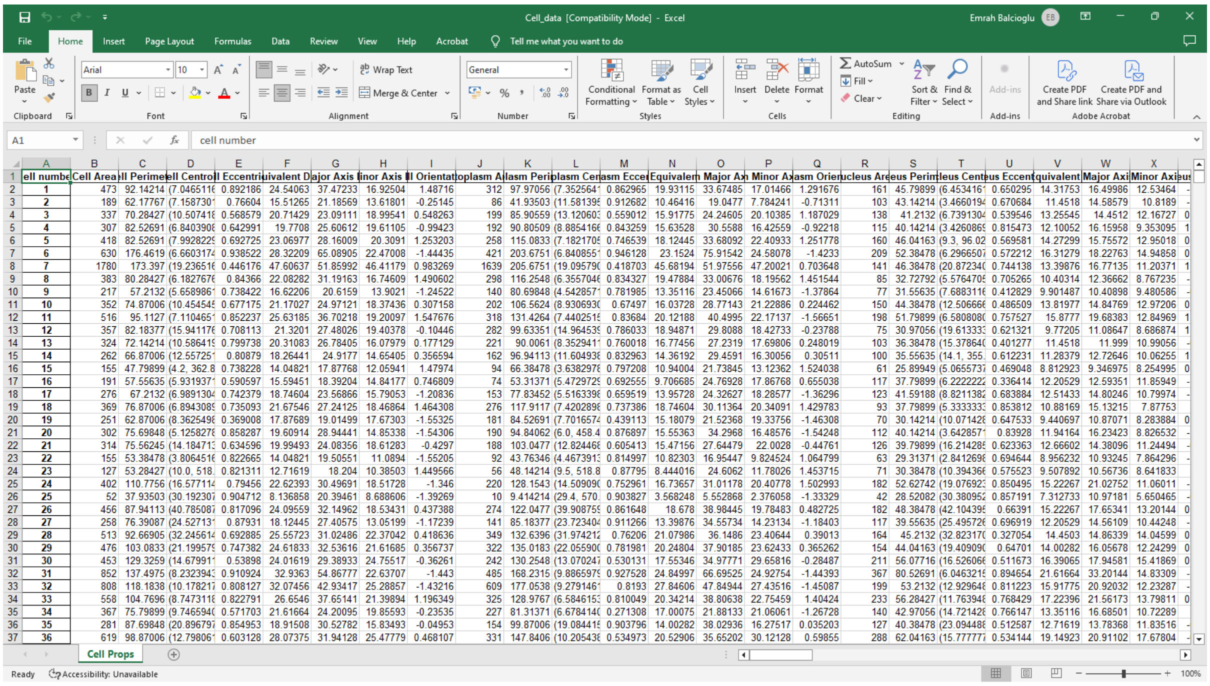

Data Analysis

Pressing the `Data Analysis` opens a new window, where you can directly quantify cell types, and export them, from TME-A. An example can be seen below:

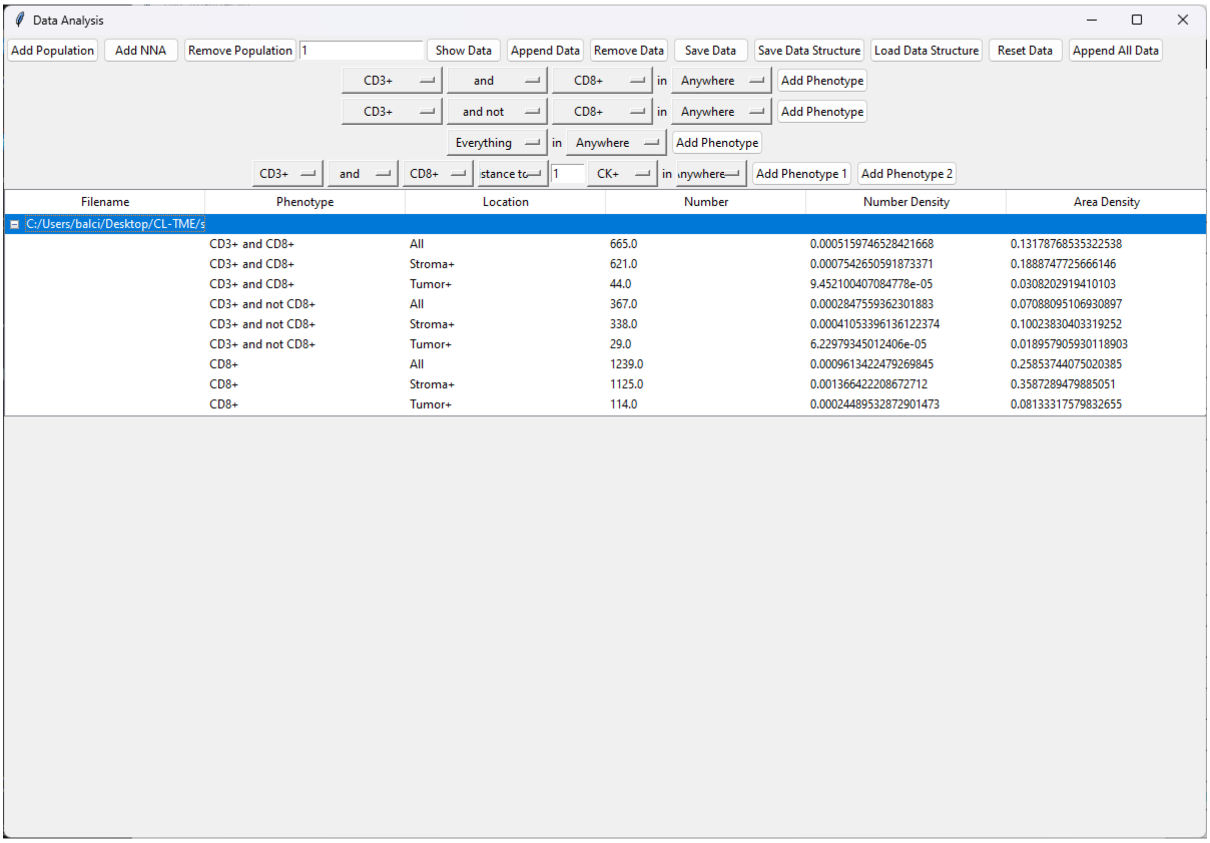

Comparing your analysis to published results

Click `Load Image`, and reload the image "sample image (1).tif". Note that if you have restarted the software, you would have to redo "Channel Setup". Click `Data Analysis -> Reanalyze Using default settings` You should end up more or less with the same analysis you have performed above. You can of course compare:

- The individual images, by going through images in the main window
- The cellular data by adding quantification from both analysis at the `Data Analysis` module (Note that here the data is displayed PER IMAGE, so if you have two different analysis on the same image open, only the latest analysis will be displayed. You can see analysis from both images by renaming the image before loading it)
- The analysis parameters through the `Data Analysis -> Display Log`, and pressing `Print params`. You would then have to change your analysis to the other (main window, dropdown at right bottom) and press `Print params` again.

Final points

Hope you made it here and are excited about TME-A. This tutorial does not cover all the aspects of the TME-A. So feel free to explore and contact us with any questions, feedback, amendments you see necessary and bug reports.
