# Supplementary figures and images for: TME-analyzer: a new interactive and dynamic image analysis tool that identified immune cell distances as predictors for survival of triple negative breast cancer patients (part 2 of 2)
Source: Npj Imaging. 2024 Jul 25;2:21. doi: 10.1038/s44303-024-00022-6 (PMC12118654; doi:10.1038/s44303-024-00022-6)

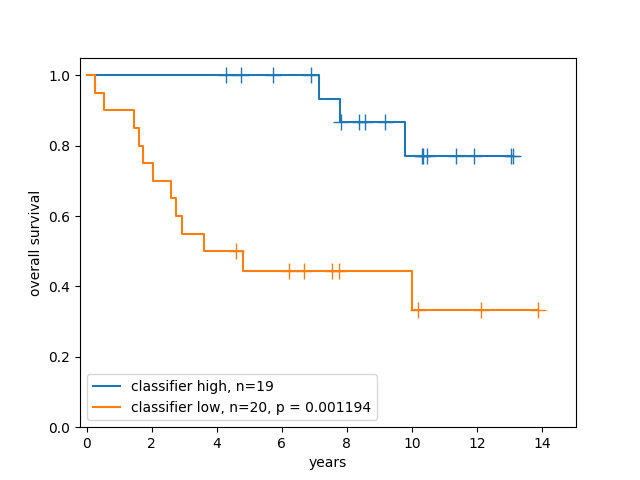

Supplement: Supplementary file 10 — varying parameter classifier performance [file 44303_2024_22_MOESM10_ESM.zip › top_28_keys_validation.png]

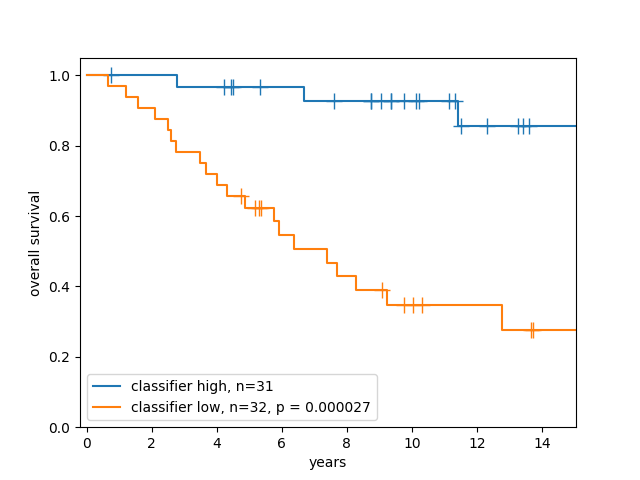

Supplement: Supplementary file 10 — varying parameter classifier performance [file 44303_2024_22_MOESM10_ESM.zip › top_29_keys_discovery.png]

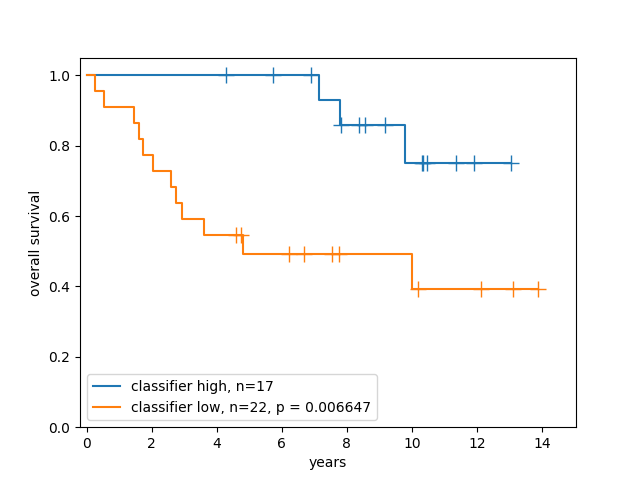

Supplement: Supplementary file 10 — varying parameter classifier performance [file 44303_2024_22_MOESM10_ESM.zip › top_29_keys_validation.png]

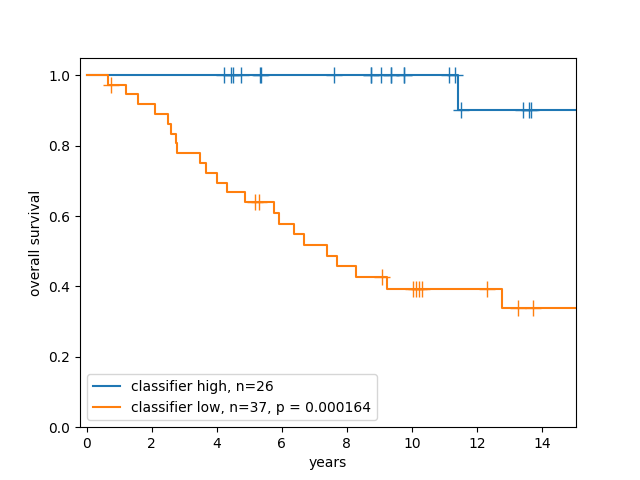

Supplement: Supplementary file 10 — varying parameter classifier performance [file 44303_2024_22_MOESM10_ESM.zip › top_3_keys_discovery.png]

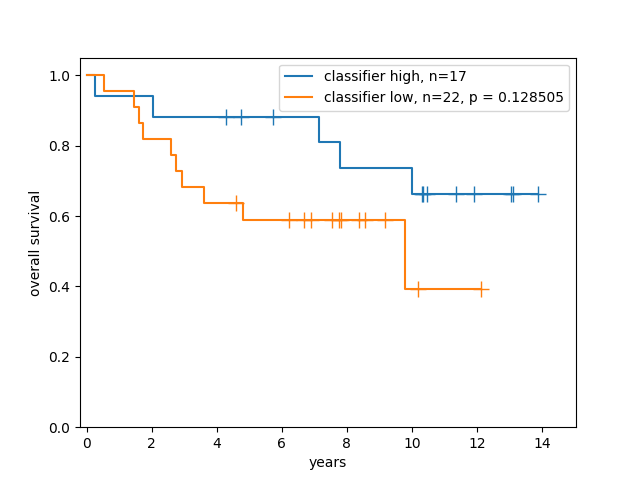

Supplement: Supplementary file 10 — varying parameter classifier performance [file 44303_2024_22_MOESM10_ESM.zip › top_3_keys_validation.png]

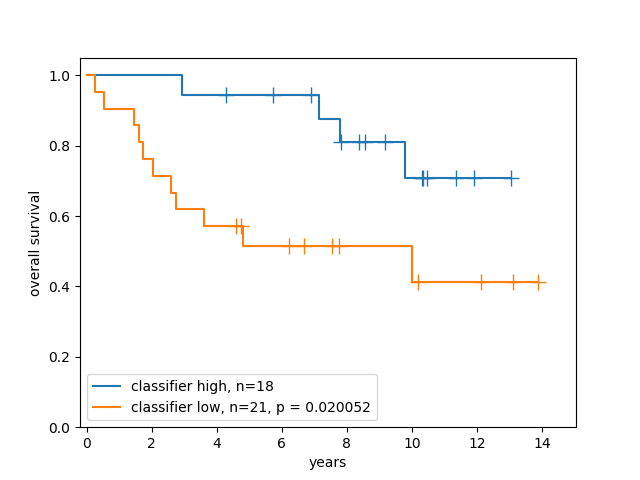

Supplement: Supplementary file 10 — varying parameter classifier performance [file 44303_2024_22_MOESM10_ESM.zip › top_31_keys_validation.png]

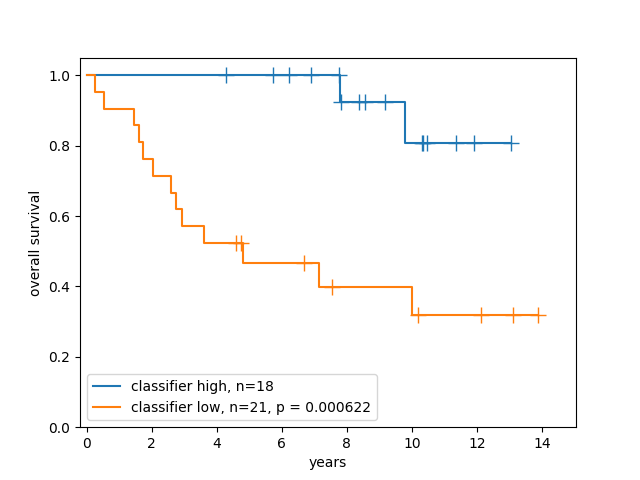

Supplement: Supplementary file 10 — varying parameter classifier performance [file 44303_2024_22_MOESM10_ESM.zip › top_33_keys_validation.png]

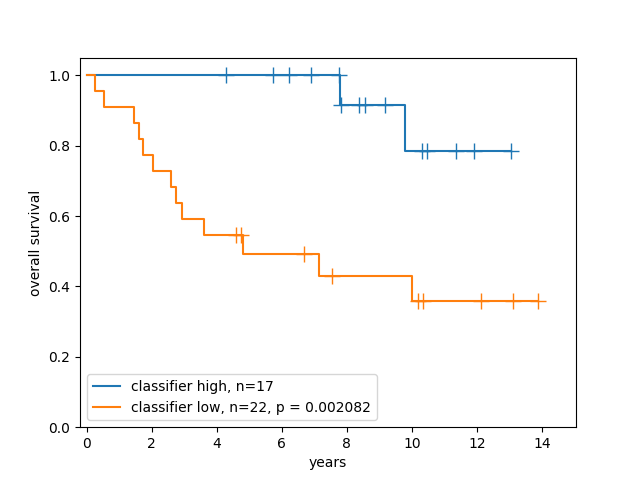

Supplement: Supplementary file 10 — varying parameter classifier performance [file 44303_2024_22_MOESM10_ESM.zip › top_34_keys_validation.png]

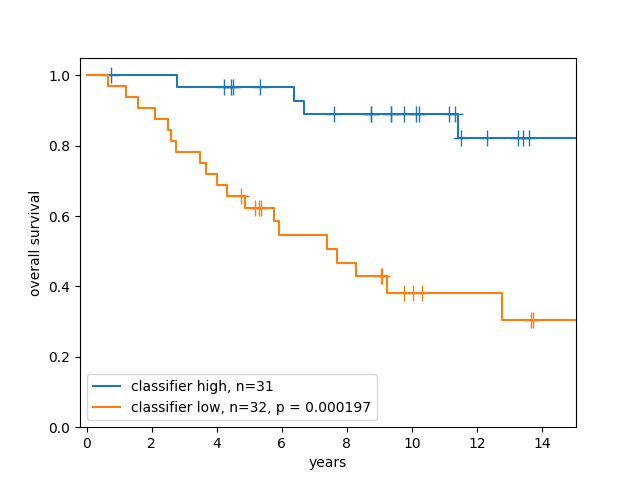

Supplement: Supplementary file 10 — varying parameter classifier performance [file 44303_2024_22_MOESM10_ESM.zip › top_35_keys_discovery.png]

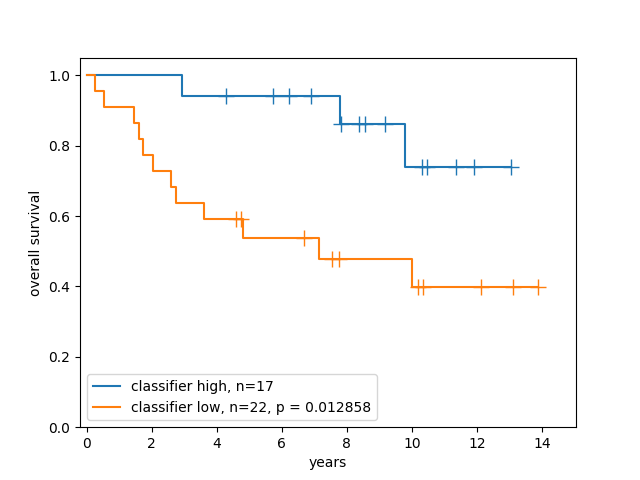

Supplement: Supplementary file 10 — varying parameter classifier performance [file 44303_2024_22_MOESM10_ESM.zip › top_36_keys_validation.png]

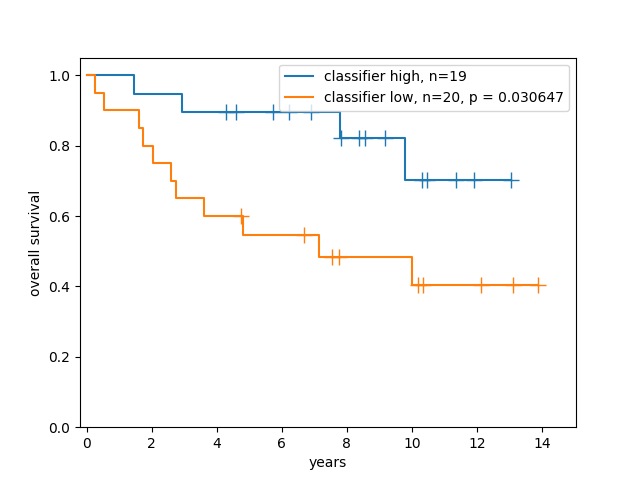

Supplement: Supplementary file 10 — varying parameter classifier performance [file 44303_2024_22_MOESM10_ESM.zip › top_37_keys_validation.png]

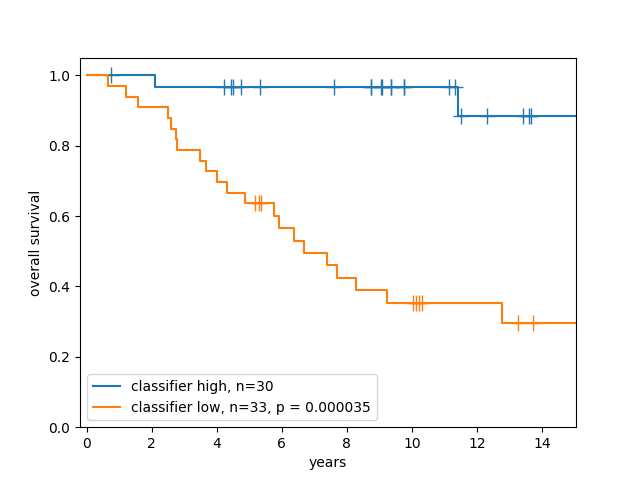

Supplement: Supplementary file 10 — varying parameter classifier performance [file 44303_2024_22_MOESM10_ESM.zip › top_4_keys_discovery.png]

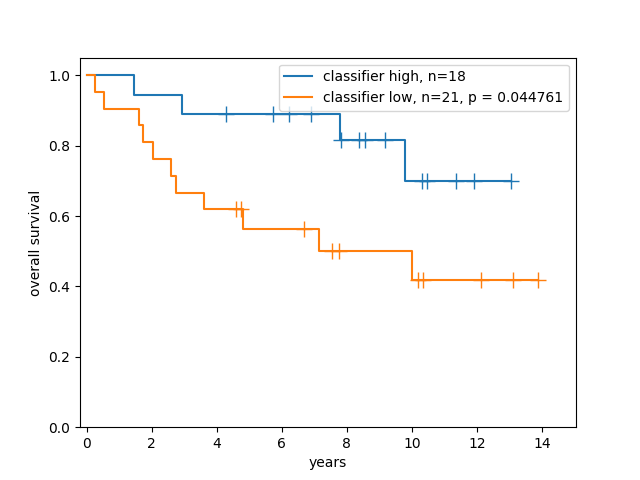

Supplement: Supplementary file 10 — varying parameter classifier performance [file 44303_2024_22_MOESM10_ESM.zip › top_40_keys_validation.png]

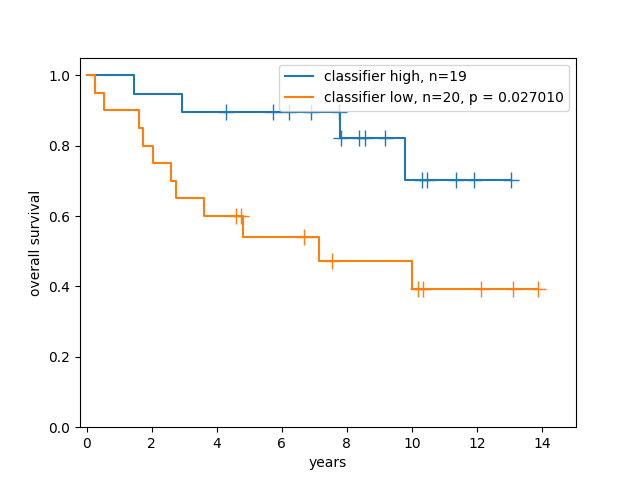

Supplement: Supplementary file 10 — varying parameter classifier performance [file 44303_2024_22_MOESM10_ESM.zip › top_42_keys_validation.png]

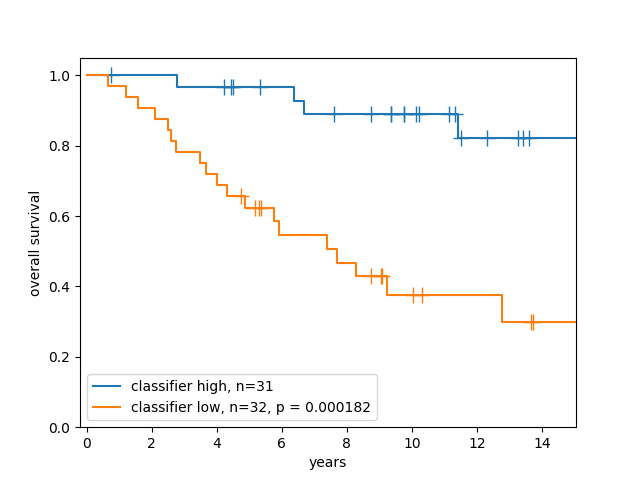

Supplement: Supplementary file 10 — varying parameter classifier performance [file 44303_2024_22_MOESM10_ESM.zip › top_44_keys_discovery.png]

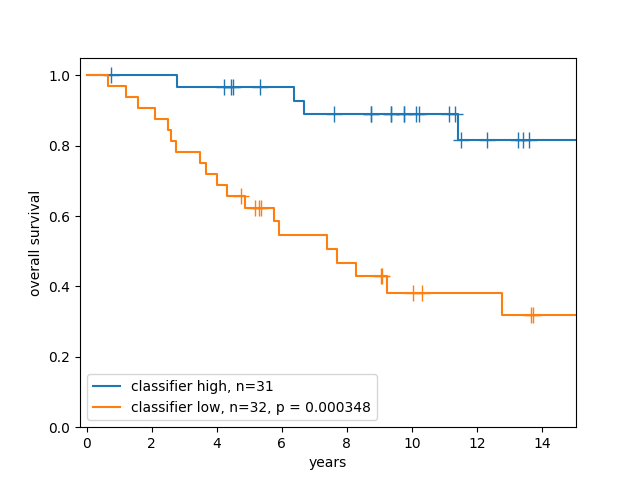

Supplement: Supplementary file 10 — varying parameter classifier performance [file 44303_2024_22_MOESM10_ESM.zip › top_45_keys_discovery.png]

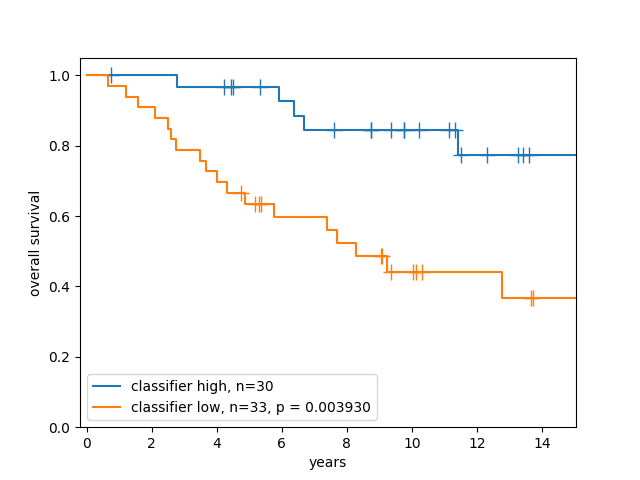

Supplement: Supplementary file 10 — varying parameter classifier performance [file 44303_2024_22_MOESM10_ESM.zip › top_46_keys_discovery.png]

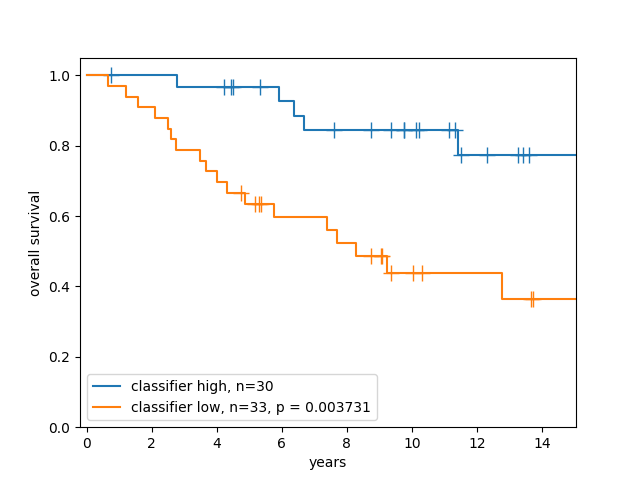

Supplement: Supplementary file 10 — varying parameter classifier performance [file 44303_2024_22_MOESM10_ESM.zip › top_47_keys_discovery.png]

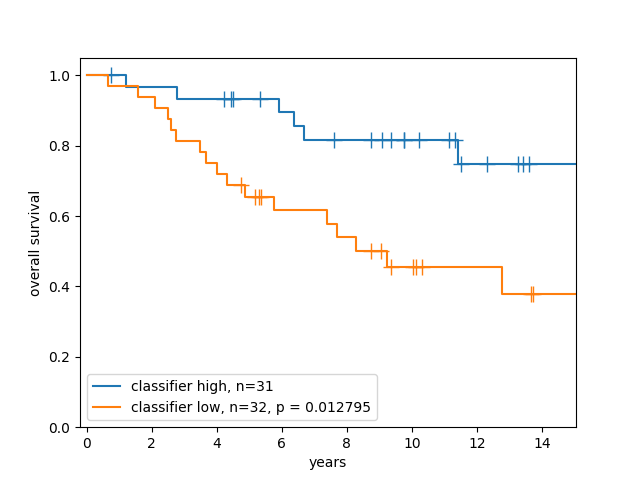

Supplement: Supplementary file 10 — varying parameter classifier performance [file 44303_2024_22_MOESM10_ESM.zip › top_48_keys_discovery.png]

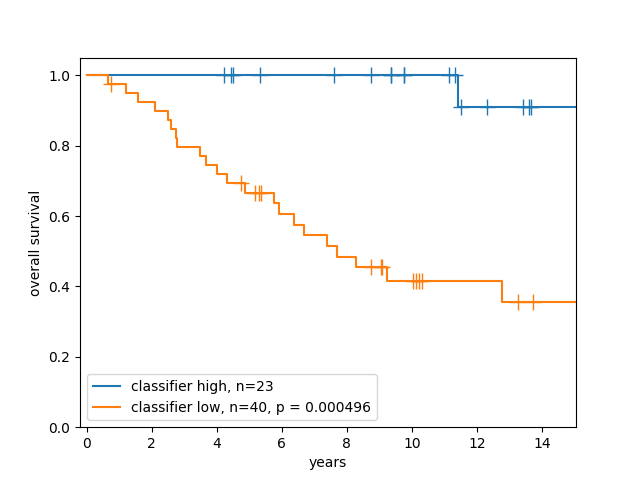

Supplement: Supplementary file 10 — varying parameter classifier performance [file 44303_2024_22_MOESM10_ESM.zip › top_5_keys_discovery.png]

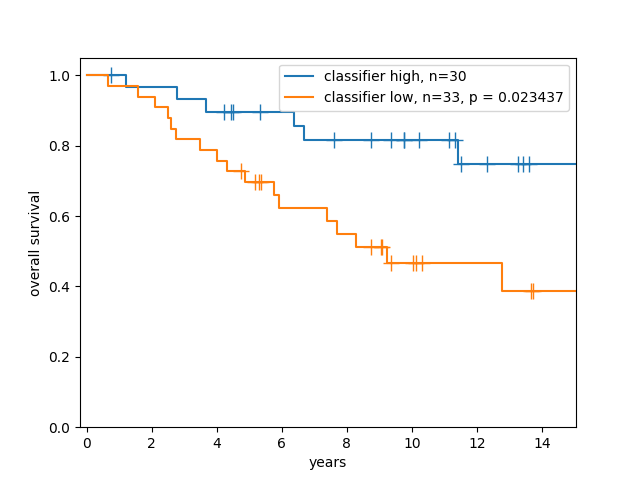

Supplement: Supplementary file 10 — varying parameter classifier performance [file 44303_2024_22_MOESM10_ESM.zip › top_50_keys_discovery.png]

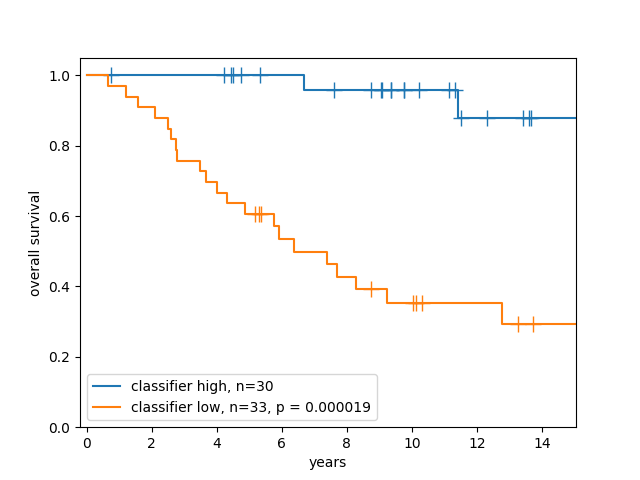

Supplement: Supplementary file 10 — varying parameter classifier performance [file 44303_2024_22_MOESM10_ESM.zip › top_6_keys_discovery.png]

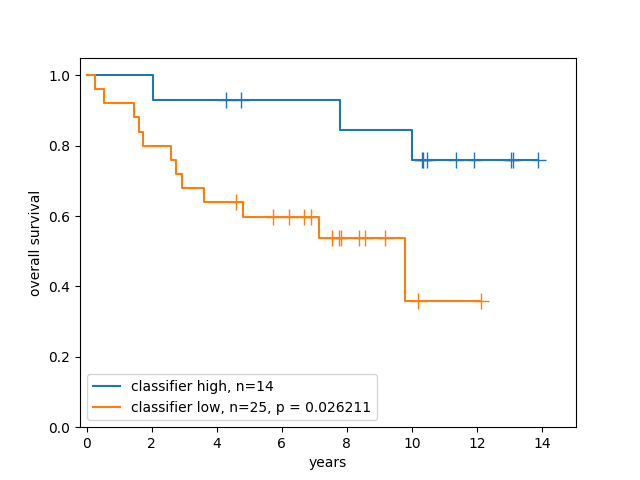

Supplement: Supplementary file 10 — varying parameter classifier performance [file 44303_2024_22_MOESM10_ESM.zip › top_6_keys_validation.png]

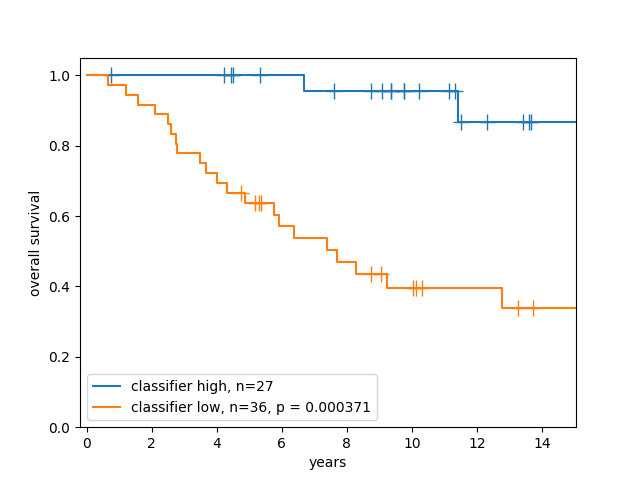

Supplement: Supplementary file 10 — varying parameter classifier performance [file 44303_2024_22_MOESM10_ESM.zip › top_7_keys_discovery.png]

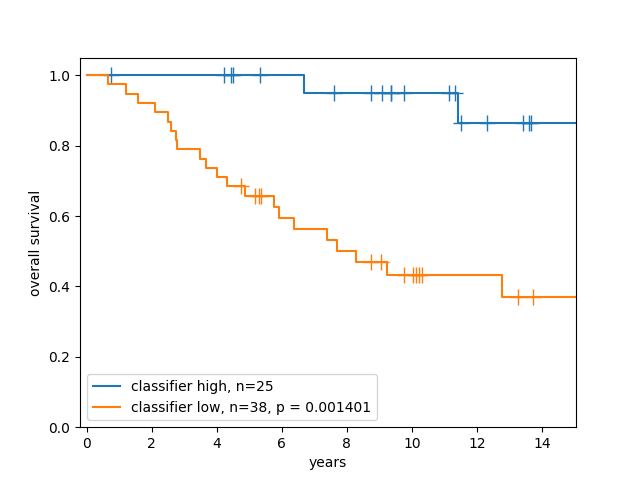

Supplement: Supplementary file 10 — varying parameter classifier performance [file 44303_2024_22_MOESM10_ESM.zip › top_8_keys_discovery.png]

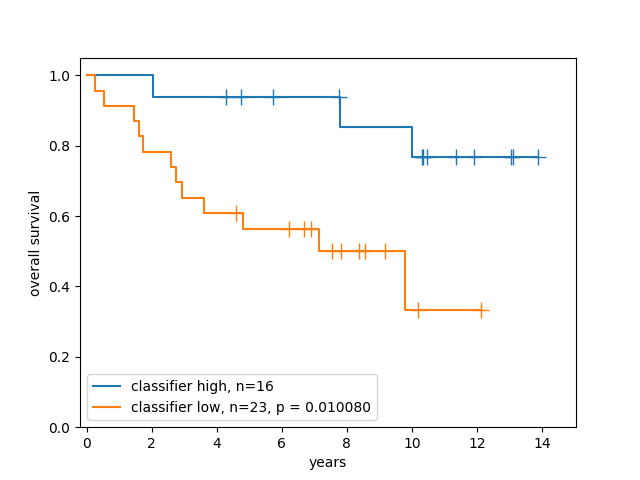

Supplement: Supplementary file 10 — varying parameter classifier performance [file 44303_2024_22_MOESM10_ESM.zip › top_8_keys_validation.png]

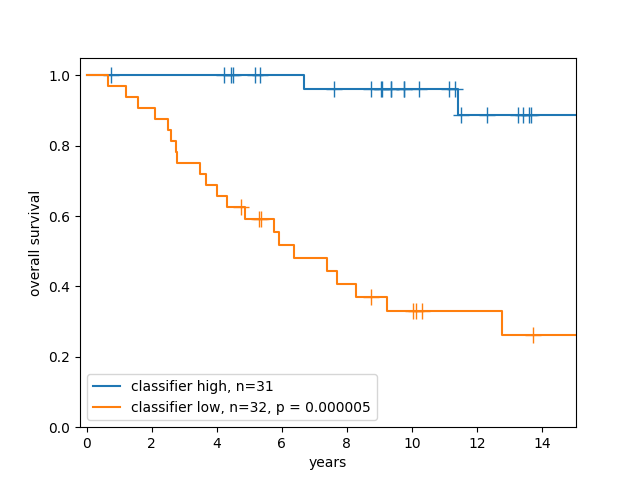

Supplement: Supplementary file 10 — varying parameter classifier performance [file 44303_2024_22_MOESM10_ESM.zip › top_9_keys_discovery.png]
